# Identification and preclinical evaluation of the small molecule, NSC745887, for treating glioblastomas via suppressing DcR3-associated signaling pathways

**DOI:** 10.18632/oncotarget.23714

**Published:** 2017-12-27

**Authors:** Li-Yun Fann, Ying Chen, Da-Chen Chu, Shao-Ju Weng, Heng-Cheng Chu, Alexander T.H. Wu, Jiann-Fong Lee, Ahmed Atef Ahmed Ali, Tsung-Chih Chen, Hsu-Shan Huang, Kuo-Hsing Ma

**Affiliations:** ^1^ Graduate Institute of Medical Sciences, National Defense Medical Center, Taipei, Taiwan, ROC; ^2^ Department of Nursing and Department of Neurosurgery, Taipei City Hospital, Taipei, Taiwan, ROC; ^3^ Department of Biology and Anatomy, National Defense Medical Center, Taipei, Taiwan, ROC; ^4^ Department of Internal Medicine, School of Medicine, College of Medicine, Taipei Medical University, Taipei, Taiwan, ROC; ^5^ The PhD Program for Translational Medicine, College of Medical Science and Technology, Taipei Medical University, Taipei, Taiwan, ROC; ^6^ Graduate Institute for Cancer Biology and Drug Discovery, College of Medical Science and Technology, Taipei Medical University, Taipei, Taiwan, ROC

**Keywords:** Naphtho [2,3-f]quinoxaline-7,12-dione, decoy receptor 3 (DcR3), [^18^F]-FDG, animal-PET

## Abstract

The small-molecule naphtha [2,3-f]quinoxaline-7,12-dione (NSC745887) can effectively inhibit the proliferation of various cancers by trapping DNA-topoisomerase cleavage. The aim of this study was to elucidate cellular responses of NSC745887 in human glioblastoma multiforme (GBM, U118MG and U87MG cells) and investigate the underlying molecular mechanisms. NSC745887 reduced the cell survival rate and increased the sub-G_1_ population in dose- and time-dependent manners in GBM cells. Moreover, NSC745887 increased expression of γH2AX and caused DNA fragmentation leading to DNA damage. Furthermore, Annexin V/propidium iodide and Br-dTP staining showed the apoptotic effect of NSC745887 in GBM cells. DNA repair proteins of ataxia-telangiectasia mutated (ATM), ATM and Rad3-related, and decoy receptor 3 also decreased with NSC745887 treatment. In addition, NSC745887 caused apoptosis by the caspase-8/9-caspase-3-poly(ADP-ribose) polymerase cascade. An *in vivo* study indicated that NSC745887 suppressed the [^18^F]-FDG-specific uptake value in brain tumors. Histological staining also indicated a decrease in Ki-67 and increases in γH2AX and cleaved caspase-3 in the brain tumor area. These data provide preclinical evidence for NSC745887 as a potential new small molecule drug for managing glioblastomas.

## INTRODUCTION

Glioblastoma multiforme (GBM) is a therapeutic challenge because it is a hard-to-treat and aggressive brain tumor and one of the most deadly forms of primary brain neoplasms [1]. A therapeutic objective is sorely needed to target GBM, a notoriously treatment-resistant brain cancer. Furthermore, the central nervous system (CNS) and the pathogenesis of GBM are complex, and much remains to be learned about putative key signaling pathways before they can be therapeutically exploited. An interplay between metabolic and oncogenic processes in brain tumors is driven by several signaling pathways that are differentially activated or silenced with both parallel and converging complex interactions [2]. Most importantly, human malignant glioma cells were engineered to release high amounts of Decoy receptor 3 (DcR3), which is overexpressed in the lungs and gastrointestinal tract [3, 4] and is associated with DcR3 binding to the fatty acid synthetase ligand (FasL) and inhibition of FasL-induced apoptosis [5]. It is noteworthy that DcR3 holds promise as a new target for treating gliomas, but still little is known regarding the molecular mechanisms underlying the small-molecule inhibitor of DcR3.

**Figure 1:**
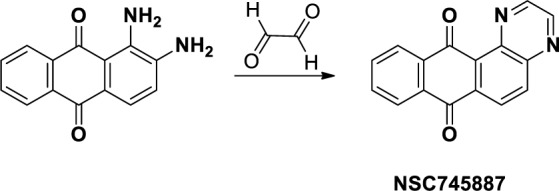
Synthesis and chemical structure of NSC745887

In view of unmet and urgent clinical needs, we were motivated by [our?] recent data from the National Cancer Institute (NCI) indicating that the CNS might respond to GBM as novel anti-glioblastoma therapeutics [6]. Several compounds were selected by the NCI for a one-dose screening program and further studies on NSC745887 where the curves cross these lines represent the interpolated values to cause 50% growth inhibition (GI_50_), total growth inhibition (TGI), and 50% cell killing (LC_50_), respectively (Supplementary Tables 1–3 in Supplementary Information). To date, only five drugs have been approved by the US FDA to treat brain tumors: everolimus, bevacizumab, carmustine (BCNU), lomustine (CCNU), and temozolomide (TMZ) [7]. Thanks to our innovative techniques in drug discovery and preliminary studies [8, 9], we developed a series of tetraheterocyclic homologues that showed exceptional potencies against several types of cancer [9–14]. From this class of compounds, NSC745887 is a naphtho[2,3-f]quinoxaline-7,12-dione (Figure 1) that exhibited a unique multilog differential pattern of activity in our earlier study [9]. To address this efforts were directed toward a synthetic small molecule (NSC745887), which exhibited unprecedented abilities such as cell-cycle regulation, and induction of apoptosis, senescence, and DNA damage in human glioblastoma cells. We also investigated the important molecular mechanisms responsible for the anticancer effects of NSC745887 against human GBM cells *in vitro* and in a xenograft animal model.

All tumors can be detected based on tracer techniques, because [^18^F]-fluorodeoxyglucose ([^18^F]-FDG) is a glucose analogue that is significantly taken up by glioma cells relative to normal cells [15]. With the very commonly used animal positron emission tomography (animal-PET), each nude mouse was subjected to an [^18^F]-FDG scan, and tumor metastasis was monitored with an *in vivo* dynamic imaging system. In this study owing to potential false-positives introduced by possible accumulation of [^18^F]-FDG in tumor cells, PET imaging was applied to improve the accuracy. The continually evolving field of examining the mechanism of GBM inhibition has prompted a more-rational use of targeted small-molecule anti-glioblastoma agents. This study aimed to investigate the toxic effect of the small-molecule, NSC745887, on GBM cell lines and the underlying mechanisms using both bioinformatics and cell-based approaches. NSC745887 exhibited potent cytotoxic and proapoptotic effects on GBM cells in dose- and time-dependent manners. Notably, NSC745887 treatment promoted G_2_/M arrest and induced apoptosis mainly via inducing DNA damage response signaling in human GBM cells. Accordingly, DcR3 in gliomas was significantly upregulated compared to normal brain tissues [5]. However, the effect of the DcR3-specific small molecule on the cell biology of glioma cells remains incompletely understood. More importantly, NSC745887 significantly induced expressions of mitochondrion-mediated proapoptotic proteins via DcR3 suppression which enhanced cell death surface receptor Fas binding to FasL that resulted in apoptotic cell death, as mediated by caspase activation. Most small-molecule anticancer drugs in use today target DNA and are part of the cellular DNA damage response (DDR) network [16]. Small-molecule inducers of the DDR pathway are of great interest, and several are under clinical development. However, the specificity of the targets and the biological roles of the phosphorylation pathway in the DDR and intricate series of interlocking mechanisms induced by NSC745887 are not known. DcR3 and DDR cancer therapy represent very attractive approaches, and potential adjuvants to standard GBM therapy are worth exploring [17–19]. Our current findings demonstrated that NSC745887-mediated GBM inhibitory effects were associated with DcR3 inhibition. More importantly, NSC745887 treatment suppressed GBM tumorigenesis in both p53 wild-type and mutant forms. This advantage may serve a broader spectrum of GBM patients in managing this malignancy in future clinical settings.

## RESULTS

### Cytotoxicity of NSC745887 towards U118MG and U87MG cells

**Figure 2:**
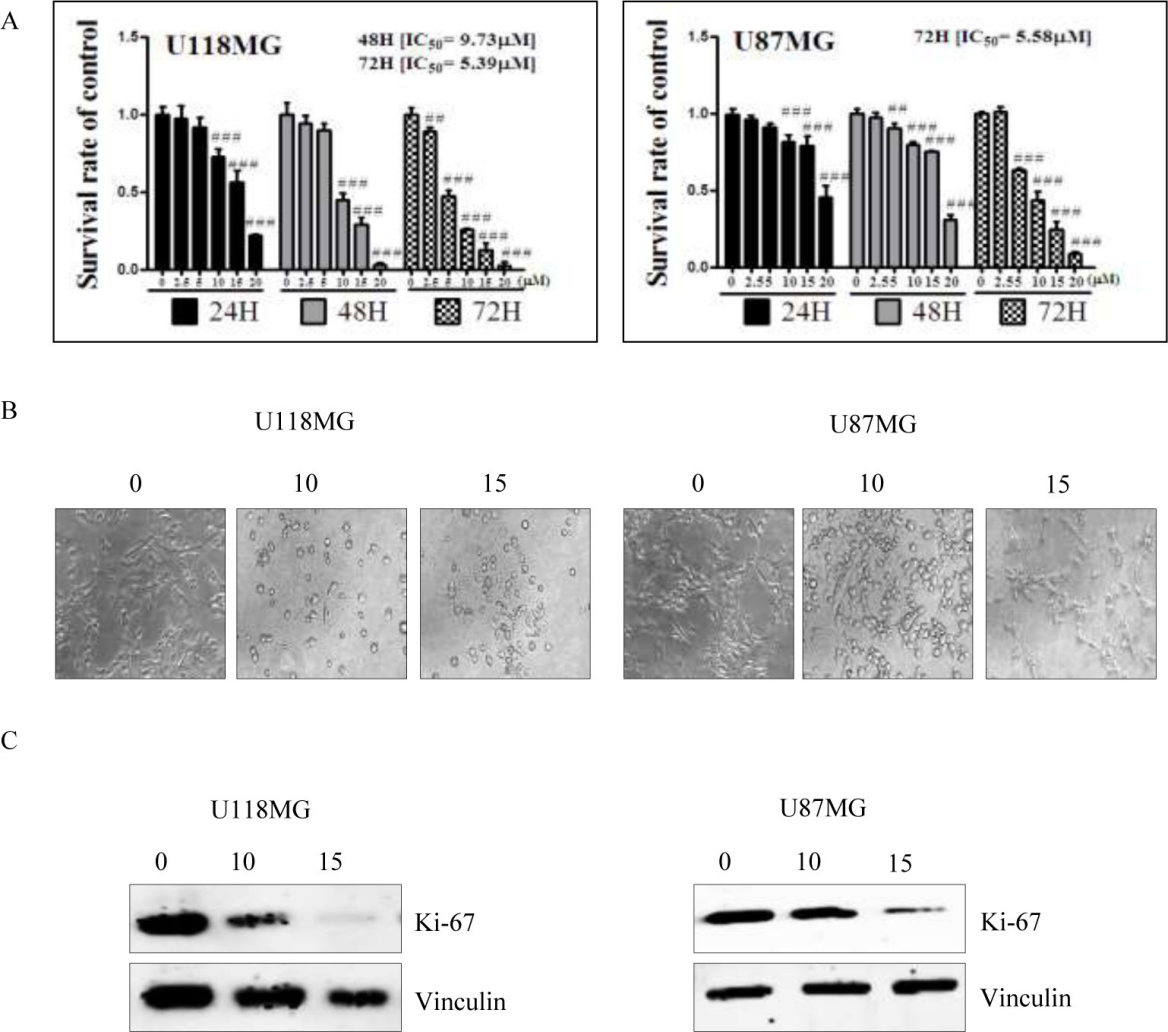
Cell cytotoxicity of NSC745887 upon treatment of U118MG and U87MG cells (**A**) U118MG and U87MG cells were treated with NSC745887 at 0, 2.5, 5, 10, 15, and 20 μM for 24, 48, and 72 h. Cells were subsequently subjected to a time-course assay. Cells were detected every 24 h, and the cell survival rate was analyzed by an MTT assay. U118MG cells were observed to be more sensitive to NSC745887, as the percentage of apoptosis had already increased by 24 h after NSC745887 treatment. U87MG cells responded more slowly, and began to undergo some apoptosis about 24 h after treatment. (**B**) Microscopy also showed that after 24 h, NSC745887-treated cells had shrunken morphologies and lower densities, which are certain markers of cell death. (**C**) Cell proliferation in the two GBM cell lines was determined using a common biomarker and Western blotting. Western blotting of the expression of Ki-67 suggested that NSC745887 decreased cell proliferation. Data are presented as the mean ± SD, with statistically significant values of ^##^*p* < 0.01, ^###^*p* < 0.001 compared to the control group.

NSC745887 was synthesized according to our previous study (Figure 1 please refer to Supplementary Figure 1 for more information on chemical synthesis and analysis) [9]. First, in order to explore the cytotoxicity of NSC745887, human glioblastoma cells (U118MG and U87MG) were treated with NSC745887 for 24, 48, and 72 h, and the cytotoxic effects were evaluated via an MTT assay. Cell morphological changes were observed with a light microscope, and significantly decreased expression of Ki-67 was found using a Western blot analysis. As shown in Figure 2 and Supplementary Figure 2, NSC745887 inhibited the proliferation of both U118MG and U87MG cells, and the cytotoxic effects were specific. To evaluate the dose- and time-dependent effects on cell viability, we performed an MTT assay after exposure of U118MG and U87MG cells to different concentrations of NSC745887 for 24, 48, and 72 h (Figure 2A). U118MG cells began to undergo apoptosis at about 24 h after treatment with 10 μM NSC745887, and more than 80% of cells had undergone apoptosis after 48 h. U87MG cells displayed signs of apoptosis after 24 h at 10 μM, and more than 80% of cells had undergone apoptosis after 72 h. Our data suggested that U118MG and U87MG cells are sensitive to NSC745887. Characteristic morphological features of apoptotic cells included shrinkage of the cell volume and membrane-bound apoptotic bodies that prominently appeared following treatment of cells with NSC745887 (Figure 2B). Next, we observed expressions of Ki-67 in both GBM cell lines using immunoblotting; vinculin was used as a loading control [20, 21]. Even though Ki-67 is strongly associated with tumor cells and is a marker of cell proliferation, we found that the Ki-67 level was strongly suppressed in U118MG cells treated with NSC745887. Similar observations were seen in U87MG cells (Figure 2C). These results are consistent with previous reports and suggest that NSC745887 causes apoptosis in U118MG and U87MG cells.

### NSC745887 induces dose- and time-dependent apoptosis and GBM cell-cycle arrest in the G_2_/M phase

**Figure 3:**
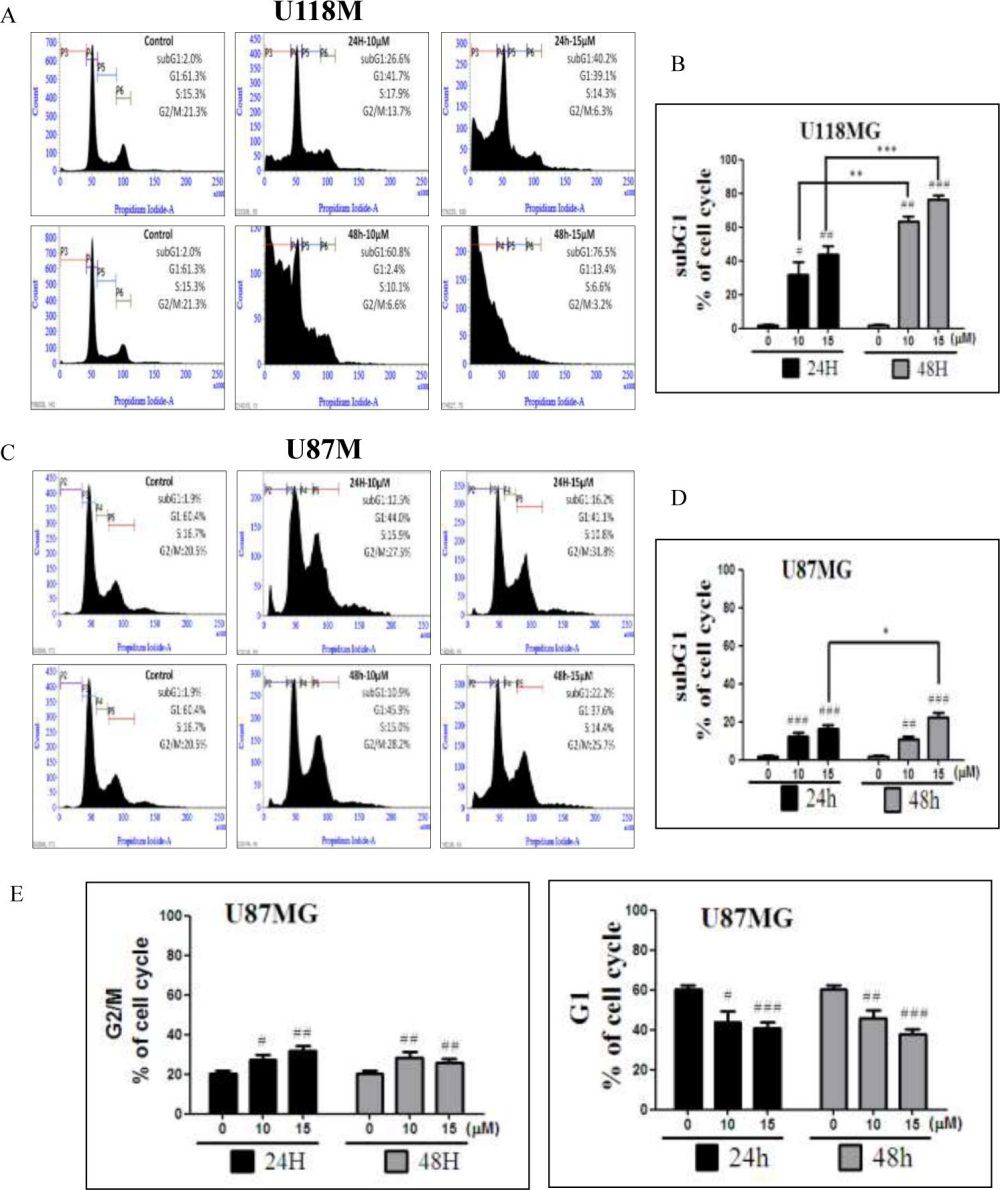
Regulation of the cell cycle by NSC745887 treatment of U118MG and U87MG cells (**A**) Cell-cycle phase distributions were analyzed with increasing doses of NSC745887 (10 and 15 µM) for 24 and 48 h, and representations of the cell-cycle modes of U118MG cells. (**C**) U87MG cells are shown. (**B**, **D**) Quantitative analyses of U118MG and U87MG cell populations in the sub-G_1_ phase using BD FACSuite analytical software. (**E**) Quantitative analyses of U87MG cell populations in the G_2_/M and G_1_ phases. Data are presented as the mean ± SD; statistical significance is indicated by ^#^ *p* < 0.05, ^##^ *p* < 0.01, ^###^ *p* < 0.001 compared to the control group. ^*^ *p* < 0.05, ^**^ *p* <0.01,^***^ *p* < 0.001, compared to the same concentration at 24 and 48 h.

In order to further investigate the underlying mechanisms of NSC745887, cell-cycle patterns of U118MG and U87MG cells subjected to different doses of NSC745887 for 24 and 48 h were scrutinized. We performed a flow cytometric analysis of PI-stained cells to study cell-cycle progression after treatment with NSC745887. Cell-cycle populations of GBM cells were compared at 24 and 48 h after treatment with various concentrations of NSC745887 as shown in Figure 3 and Supplementary Figure 3 in the Supplementary Information. NSC745887 effectively caused increased cell-cycle arrest in the G_2_/M phase with higher concentrations and longer durations, and the proportion of hypodiploid cells increased in dose- and time-dependent manners. More specifically, even though the ratio of cells in the sub-G_1_ phase was obviously higher, accumulation of cells in the G_2_/M phase resulted in apoptosis. In U118MG cells, as illustrated in Figure 3A and 3B, proportions of cells in the sub-G_1_ phase, which had the appearance of apoptosis, had increased to 26.6% and 40.2% at 24 h after treatment with 10 and 15 μM of NSC745887, and were elevated to 69.8% and 76.5% at 48 h after treatment, respectively. U87MG cells also showed similar results at the sub-G_1_ phase (Figure 3C, 3D). Moreover, in U87MG cells, NSC745887 increased the percentage of cells in the G_2_/M phase while decreasing the G_1_ fraction (Figure 3E). Our data suggest that NSC745887 induced apoptosis and G_2_/M cell-cycle arrest. Although both cell lines (U118MG and U87MG) responded to NSC745887 treatment, U118MG cells were more sensitive to NSC745887 than were U87MG cells. Proportions of cells with 4N DNA content, which indicates G_2_/M blockage, showed increases of 27.5% and 31.8% in cells respectively treated with 10 and 15 μM of NSC745887 (Figure 3E), suggesting that NSC745887 can cause G_2_/M arrest in GBM cells. These results suggested that NSC745887 caused apoptosis of GBM cells in dose- and time-dependent manners.

### Induction of morphological and biochemical features of apoptosis after NSC745887 treatment

**Figure 4:**
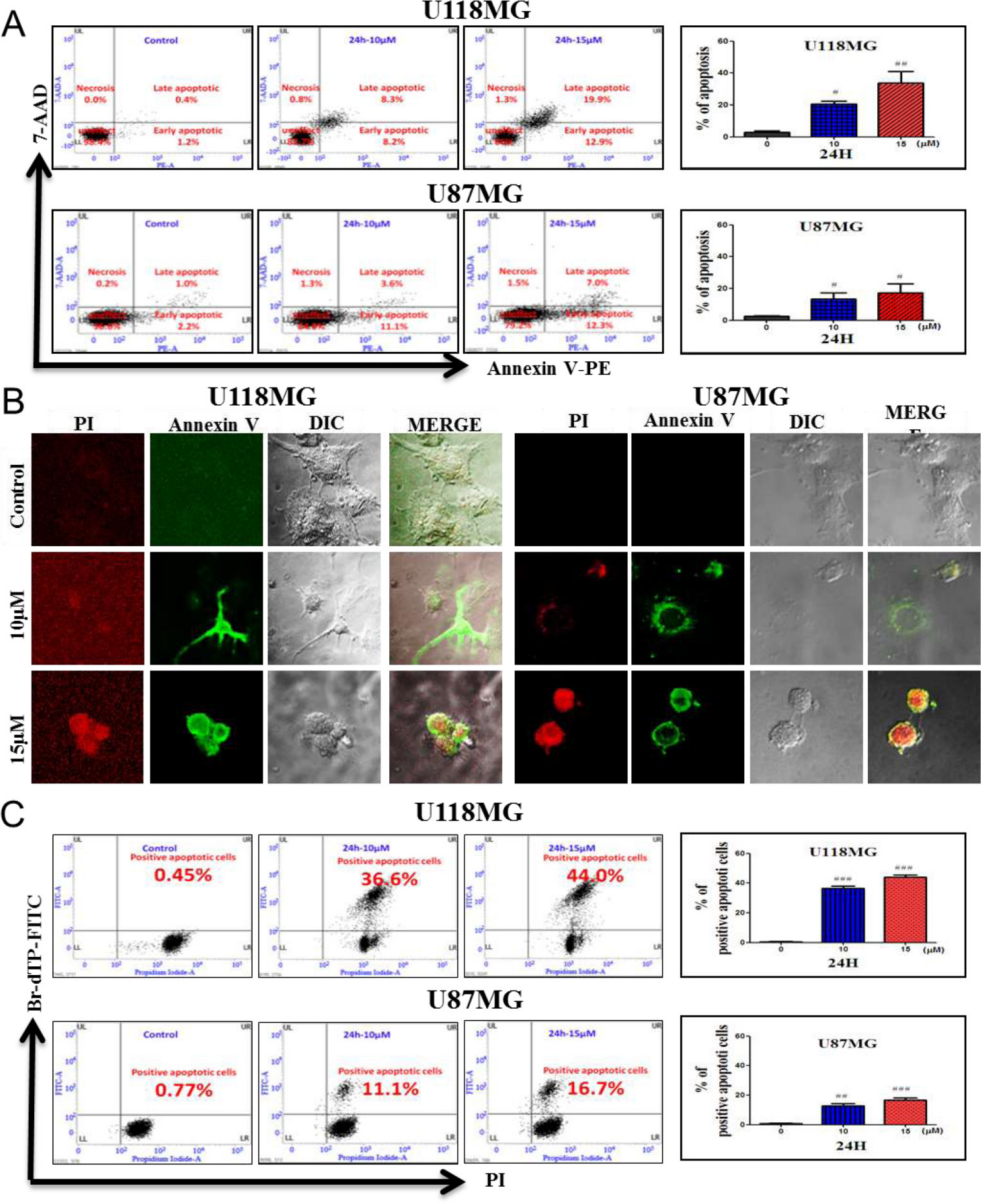
Induction of morphological and biochemical features of apoptosis in human glioblastoma cells Treatments: control (CTL), and incubation with 10 and 15 µM NSC745887 for 24 h. (**A**) Annexin V-PE/7-AAD double-staining and flow cytometric analysis of apoptotic populations after treatment. NSC745887 induced a significant population of cells in the A4 area, indicating induction of a biochemical feature of apoptotic death. (**B**) Confocal microscopic imaging to examine morphological features of apoptosis after cells were stained with Annexin V-FITC and propidium iodide. (**C**) Determination of the percentage of apoptosis based on morphological and biochemical features revealed by a TUNEL assay. A significant difference between the control (CTL) and treatment is indicated by ^*^ *p* < 0.05 or ^**^p < 0.01. Results are representative of three independent experiments.

Biochemical features of apoptosis were examined using a flow cytometric analysis and confocal microscopic imaging (Figure 4, Supplementary Figure 4 in Supplementary Information). Apoptosis was originally defined by structural alterations in cells observable by transmitted light and electron microscopy [19, 22]. Annexin V-conjugated PE- and 7 AAD-stained cells showed features of apoptosis after NSC745887 treatment in dose- and time-dependent manners in both the U118MG and U87MG cell lines (Figure 4A). An increase in populations of Annexin V PE-positive and 7AAD-negative or -positive cells in the A4 area indicated the occurrence of apoptosis, as shown in both U118MG and U87MG cell lines with various doses of NSC745887. Based on the flow cytometric analysis of Annexin V PE-positive cells, percentages of apoptotic cells in the U118MG and U87MG cell lines were determined (right panels of Figure 4A). Apoptosis rates without treatment, and with treatment with 10 or 15 µM NSC745887 for 24 h were 1.6%, 16.5%, and 32.8% in U118MG cells and 3.2% 14.7%, and 19.3% in U87MG cells, respectively. Compared to control cells, 10 µM NSC745887 very significantly increased percentages of Annexin V PE-positive populations in both cell lines. The increase in Annexin V PE-positive cells after NSC745887 treatment indicated a prominent biochemical feature of apoptosis in GBM cells. To verify apoptotic events in NSC745887-treated cells, phosphatidylserine of external membranes and nuclei of cells stained with Annexin V-FITC and PI were imaged by confocal microscopy. As shown in Figure 4B, the apoptotic program was characterized by condensation of the cytoplasm and nuclei in both treated cell lines. We then utilized a TUNEL assay, in which the TdT enzyme catalyzes a template-independent addition of Br-dUTP to the 3′-hydroxyl (OH) termini of double- and single-stranded DNA, to detect DNA damage events. Figure 4C shows results of the flow cytometric analysis of Br-dUTP-FITC/PI-stained U118MG and U87MG cells at 24 h after treatment with various concentrations of NSC745887. The upper right quadrant of the cytograms represents the number of cells exhibiting DNA fragmentation, which was positive for Br-dUTP binding and showed PI uptake. The apoptotic cell population of U118MG cells significantly increased from 0.45% in untreated cells to 36.6% and 44.0% in 10 and 15 μM NSC745887-treated cells at 24 h, respectively; also, proportions of U87MG cells with fragmented DNA content increased from 0.77% to 16.7%. Overall, apoptosis emerged as the major mechanism of cell death promoted by NSC745887 in GBM cells.

### Impacts of ATM and ATR phosphorylation on NSC745887 sensitivity

**Figure 5:**
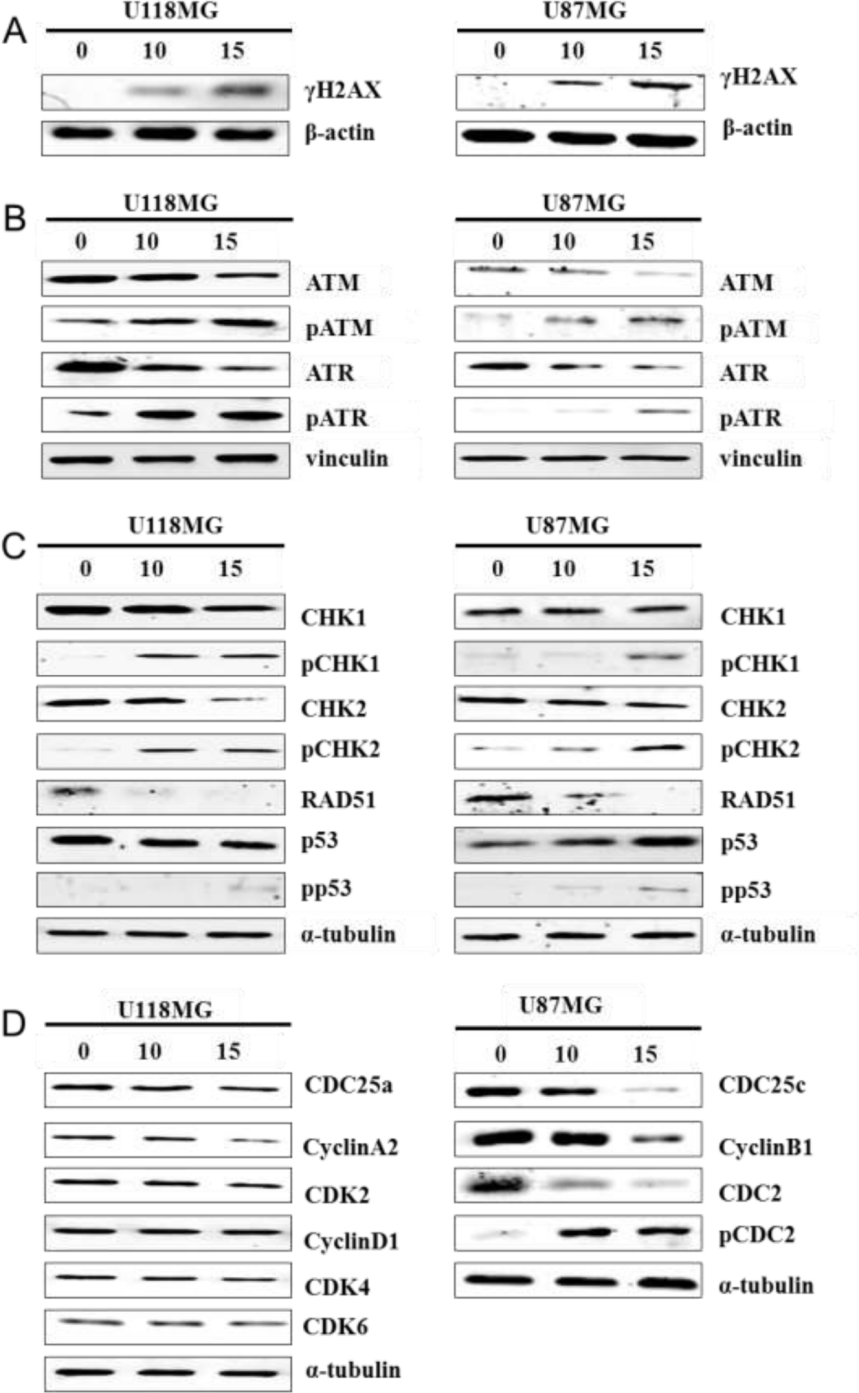
NSC745887 activates ATM and ATR signaling Protein expression levels of (**A**) γH2AX; (**B**) ATM, phosphorylated (p)-ATM, ATR, p-ATR, (**C**) CHK1, p-CHK1, CHK2, p-CHK2, RAD51, p53, p-p53 (**D**) CDC25a, cyclin A2, CDK2, cyclin D1, CDK4/6, CDC25c, cyclin B1, CDC2, and p-CDC2 were detected in cells treated with or without NSC745887 (10 or 15 μM) for 24 h using Western blotting. β-Actin, vinculin, and α-tubulin were used as loading controls. Data are presented as the mean ± SD; statistical significance is indicated by ^#^ *p* < 0.05, ^##^ *p* < 0.01, ^###^ *p* < 0.001 compared to the control.

In our previous study, we reported that NSC745887 induced DNA damage caused by topoisomerase inhibition in HeLa cells [8]. The phosphorylated form of H2AX on serine139 [23], which mediates retention of double-strand DNA break (DSB)-responsive proteins on DSB-associated chromatin [24], is a sensitive marker of detectable reactions to DSBs [25]. Therefore, we determined its effect on DNA damage in U118MG and U87MG cells at 24 h after NSC745887 treatment by a Western blot analysis (Figure 5, Supplementary Figure 5 in Supplementary Information). Expression of phosphorylated H2AX (γH2AX) was detected in a majority of treated cells. As expected, these data indicated that NSC745887 triggered dose-dependent upregulation of H2AX phosphorylation which was correlated with DNA damage. Cells with damaged DNA are hard to restore and may experience apoptosis and cell-cycle arrest; they may also initiate DNA damage responses by a variety of protein kinases. H2AX containing a conserved SQ motif (S139 Q140) is recognized as the core target motif of serine/threonine kinases including ataxia-telangiectasia mutated (ATM) and RAD3-related (ATR), and initiate ATM and ATR phosphorylation following H2AX phosphorylation after DNA damage [24]. In the DNA damage signaling pathway, checkpoint kinase 1 (CHK1), CHK2, RAD51 [26], and p53 [27] are activated by ATM and ATR to regulate the cell cycle [28], initiate apoptosis [29], or repair DNA damage [30]. Thus, we also evaluated levels of phosphorylated and total protein DNA damage-response factors in NSC745887-treated U118MG and U87MG cells. As shown in Figure 5B and 5C, NSC745887 resulted in phosphorylation of ATM/ATR and CHK1/CHK2, while RAD51 expression was significantly suppressed and p53 was upregulated in U87MG cells. As we obtained significant DNA damage-response signaling in GBM cells with NSC745887 treatment, we also examined expressions of cell cycle-associated proteins, such as the phosphatase activity of cell division cyclin 25 (CDC25) which is inactivated by CHK1/CHK2 [31]. The CDC25c protein activates the cyclin B1/CDC2 complex leading to G_2_/M phase arrest [32] as well as CDC25a regulation at the S phase [33]. As shown in Figure 5D, NSC745887 resulted in suppression of CDC25c and cyclin B1 as well as CDC2 phosphorylation in U87MG cells. In U118MG cells, we observed no cell cycle-associated protein changes under NSC745887 treatment. Overall, these results indicated that NSC745887 could induce DNA damage in GBM cells and activate the ATM/ATR and CHK1/CHK2 pathways; these effects may trigger the arrest of cell-cycle progression at the G_2_/M phase and promote apoptosis.

### NSC745887 engages intrinsic and extrinsic apoptotic pathways

**Figure 6:**
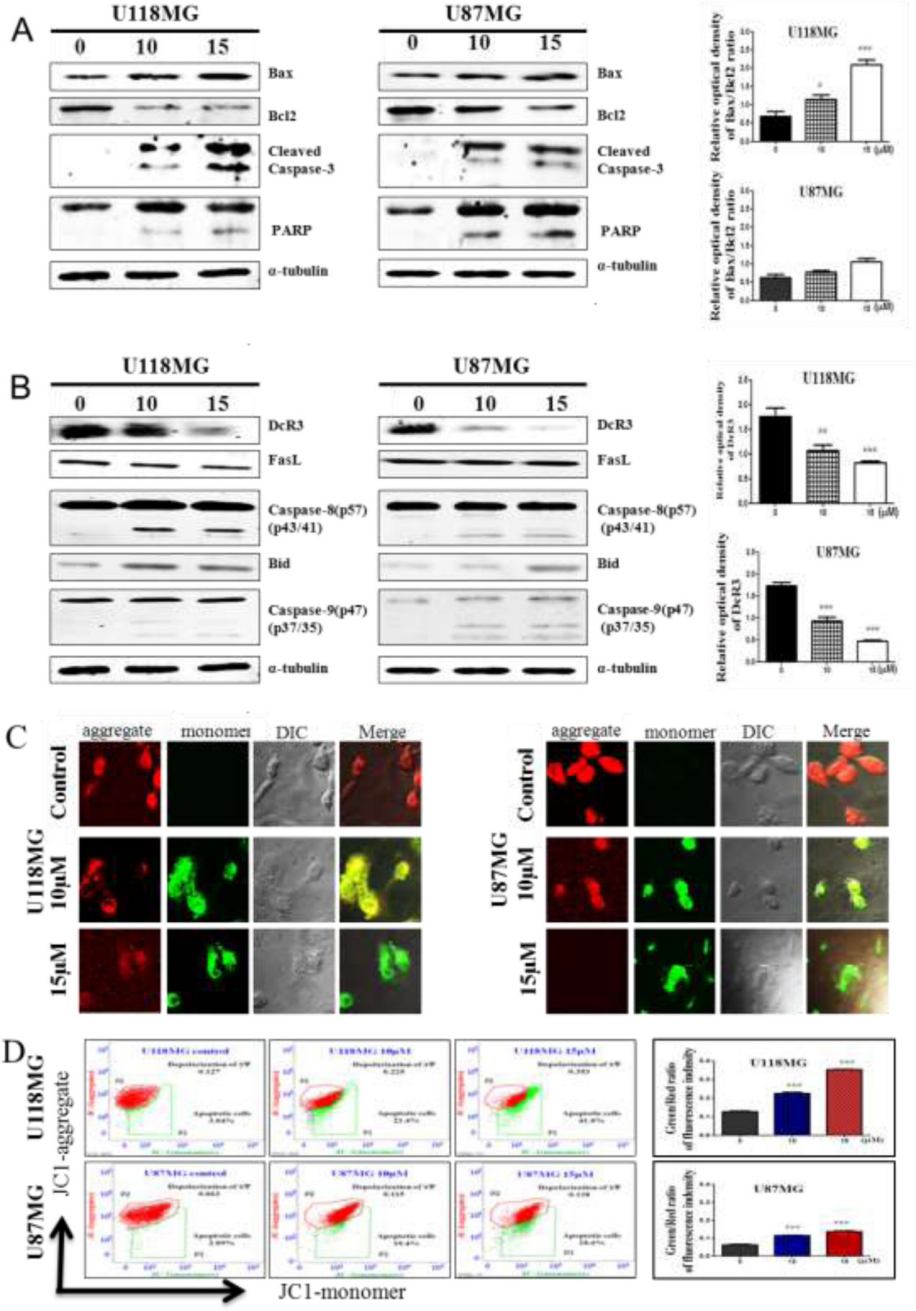
NSC745887 treatment induces the intrinsic and extrinsic apoptotic pathways in GBM cell lines Protein expression levels of (**A**) Bax, Bcl2, cleaved caspase-3, and poly(ADP ribose) polymerase (PARP); (**B**) DcR3, FasL, cleaved caspase-8, Bid, and cleaved caspase-9 were detected in cells treated with or without NSC745887 (10 or 15 μM ) for 24 h by Western blotting. α-Tubulin was used as a loading control. (**C**) Fluorescence staining of JC-1, which shows the mitochondrial membrane potential change, was analyzed by confocal microscopy to show the cell morphology and then (**D**) was detected by a flow cytometric analysis. Data are presented as the mean ± SD; statistical significance is indicated by ^#^ *p* < 0.05, ^##^ *p* < 0.01, ^###^ *p* < 0.001 compared to the control.

We next studied the action of the intrinsic apoptotic pathway through the DDR, which increases proapoptotic cysteinyl aspartic acid-protease-3 (caspase-3) and poly(ADP-ribose) polymerase (PARP) expressions and downregulates B-cell lymphoma protein 2 (Bcl2)-associated X protein (Bax) heterodimer formation, through which Bax promotes cell death by competing with Bcl2 to adjust mitochondrial dynamics during the apoptotic process [27, 34]. Following mitochondrial membrane depolarization, initiation of the assembly of the apoptosome results in activation of the initiator, caspase-9, and the downstream effector, caspase-3, and ultimately cell death [35]. DcR3 expression is elevated in tumor cells and is also associated with autoimmune and inflammatory diseases [36]. However, further studies on the regulation of DcR3 expression in gliomas by NSC745887 are needed to understand this remarkable expression pattern. To study the mechanism of action, efforts were directed toward how DcR3 competes with Fas in binding to FasL and inhibits FasL-induced apoptosis, which involves extrinsic signaling pathways, initiating apoptosis through transmembrane receptor-mediated interactions, and targeting effecters such as caspase-8, Bid, and Bcl2 [37]. Evaluation of the overexpression of DcR3 in GBM [38] led us to investigate its involvement in triggering apoptosis. U118MG and U87MG cells were treated with NSC745887 for 24 h and analyzed by Western blotting. As shown in Figure 6A (Figure 6, Supplementary Figure 6 in Supplementary Information), the ratio of Bax-Bcl2 was significantly upregulated, and caspase-3 and PARP were cleaved. DcR3 was also overexpressed in untreated cells and was downregulated in NSC745887-treated cells, while the affecter proteins of caspase-8 and caspase-9 were activated by the cleaved form, increasing the Bid protein level (Figure 6B). We then assessed mitochondrial function following NSC745887 treatment by first characterizing the mitochondrial membrane potential (MMP) in U118MG and U87MG cells using JC-1 staining. As shown in Figure 6C, red fluorescence was seen in control cells, indicating the presence of JC-1 dye in the aggregated form and revealing the polarization potential of mitochondrial membranes. Although the mechanism is not fully understood, NSC745887-treated cells showed enhanced green fluorescence, indicating the presence of JC-1 monomers and a depolarized MMP. JC-1 staining was further verified to be due to mitochondrial membrane depolarization using a quantitative flow cytometric analysis. It showed reduced JC-1 aggregates and amplified JC-1 monomers in NSC745887-treated cells compared to untreated cells (Figure 6D). Our results suggest the involvement of caspase-8, -9, and -3 activation and PARP division in cell death through intrinsic and extrinsic apoptotic pathways.

### [^18^F]-PET in an experimental animal model

**Figure 7:**
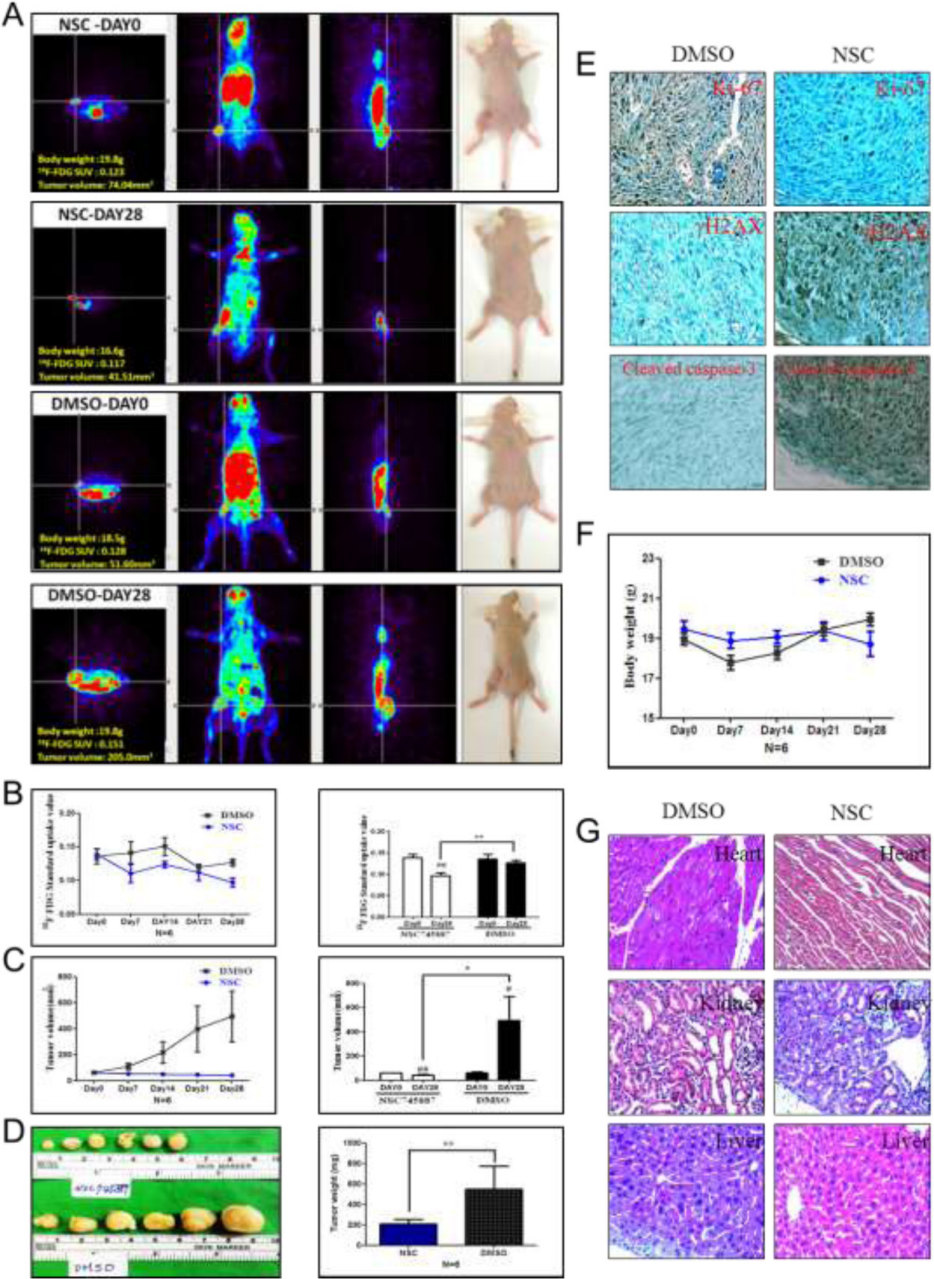
NSC745887 promotes growth inhibition in xenografts *In vivo* PET imaging data were analyzed in a NSC745887-treated group and a DMSO group using an animal-PET system. (**A**) [^18^F]-FDG PET images from 15 to 35 min in U118MG expressing xenograft-bearing mice after intraperitoneal administration of radiotracers. (**B**) Quantitative analyses of specific [^18^F]-FDG uptake values and (**C**) tumor volumes. (**D**) The tumor weight was measured at the endpoint. (**E**) Representative images of IHC staining of xenograft tumors. Protein levels of Ki-67, γH2AX, and cleaved caspase-3. (**F**) Body weights were measured during treatment. (**G**) Representative image of H&E staining of the heart, liver, and kidneys in xenograft mice. ^*^ *p* < 0.05, ^**^ *p* < 0.01 comparing days 0 and 28. ^#^ *p* < 0.05, ^##^ *p* < 0.01 comparing the NSC745887 and DMSO groups.

[^18^F]-PET is used in the clinic for staging a range of cancers and has been widely used to investigate cancers [39, 40]. The glucose analogue, 2-[^18^F]-FDG, is one of the most commonly used PET radiotracers. While clinical PET imaging has significantly expanded over the past decade, [^18^F]-FDG PET imaging is still very commonly used and is widely available. To further evaluate the efficiency of NSC745887 for treating GBM *in vivo*, we subcutaneously injected BALB/c nude xenograft mice with 10^6^ U118MG cells. Mice were treated with NSC745887 (5 mg/kg) or DMSO (control group) via an i.p. injection every day. Animal-PET scan resolution of the tumor progress showed significant differences between the control and treatment groups (Figure 7A, Supplementary Figure 7 in Supplementary Information). The mean specific uptake value of [^18^F]-FDG in the NSC745887 group (0.139 ± 0.02, *n* = 6) was consistent with that of the DMSO group (0.136 ± 0.0, *n* = 6) (*p* > 0.05) on day 0, while that of the NSC745887 group was significantly lower than that of the DMSO group on day 28 (0.097 ± 0.02 vs. 0.138 ± 0.01, respectively, *p* < 0.01) (Figure 7B). The tumor volume of the NSC745887 group (61.15 ± 6.89 mm^3^) was consistent with that of the DMSO group (64.01 ± 14.08 mm^3^) (*p* > 0.05) on day 0, while that of the NSC745887 group was significantly smaller than that of the DMSO group on day 28 (44 ± 12 vs. 496 ± 480 mm^3^, respectively, *p* < 0.05) (Figure 7C). Mice were euthanized at the endpoint of the experiment (on day 29), and tumor sizes were measured (Figure 7D). The tumor weight of the NSC745887 group (210 ± 103 mg) was significantly smaller compared to the DMSO group (548 ± 554 mg) (*p* < 0.01). An IHC analysis of tumor tissues showed that the Ki-67 level was downregulated, and γH2AX and cleaved caspase-3 levels were upregulated in NSC745887-treated mice (Figure 7E). To explore the toxicity of NSC745887, we monitored body weights of the mice. Body weights of mice in neither group greatly changed during the experiment. On day 0, the weight was 19.5 ± 0.9 mg in the treatment group and 19.01 ± 0.7 mg in the DMSO group, (*p* > 0.05), and on day 28, they were 18.7 ± 1.5 and 19.9 ± 0.8 mg, respectively, (*p* > 0.05) (Figure 7F). No damage was found in tissues of the heart, kidneys, or liver during the histopathological analysis of either group (Figure 7G). This toxicity evaluation showed that NSC745887 had no toxic effects on either group as assessed by the body weight and vital organ function in mice, which suggests that NSC745887 is safe. In conclusion, our *in vitro* studies provide a basis for screening tests to select suitable cell lines for the development of human tumor xenograft models for animal-PET imaging.

## DISCUSSION

In this study, we established a molecular basis for the efficacy of a novel small molecule and its selective and tumor-suppressive effects on human glioblastoma cells (p53 wild-type and mutated-type) *in vitro* and *in vivo*. Several discrete mechanisms of anticancer activity were proposed for NSC745887 herein, including NSC745887 induction of DNA damage and apoptosis. In addition, NSC745887 induced DNMT3a gene expression in HeLa cells [8]. On the other hand, the effect of NSC745887 on protein stability, including p53, might compensate for the low affinity of topoisomerase IIA, as demonstrated by our previous docking mode analysis [8]. NSC745887 was designed following intensive research on the biology of G-quadruplex stabilizers [9]. The design rationale comprised certain structural features shared by known quadruplex-binding small molecules, with particular emphasis on an electron-rich aromatic surface, the potential for a flat conformation, and the ability to participate in hydrogen bonding [8, 41]. We further found that NSC745887 is readily accessible in only one synthesis step that is easily scalable and amenable to molecular diversity [9]. To complement the chemically induced synthetic lethality, small-molecule inhibitors of DNA repair pathways are being intensively investigated as chemotherapeutic strategies [42, 43]. This approach analyzes DNA fragmentation, cell-cycle arrest, MMP changes and apoptosis-mediated signaling pathways and provides an opportunity to identify novel small molecules in the DDR through follow-up target identification studies. We also examined the uptake kinetics of NSC745887 in both p53 wild-type and p53-mutant GBM cell lines. These data will guide the selection of tumor types for animal studies and translational development, which are ongoing in our laboratories.

A previously established cytotoxic anticancer drug achieved its efficacy via promoting the formation of DNA DSBs and DDRs [44]. Among the many different DNA lesions, DNA DSBs are the most deleterious and are part of the cellular DDR network [45]. Our drug design strategy was to exclude false positives and select compounds with the potential for targeting DDR pathways. Based on this design, NSC745887 was synthesized and shown to promote apoptosis in GBM cells in dose- and time-dependent manners. Dissociation of the complex formed was analyzed by flow cytometry, and cell-cycle arrest was evaluated in the presence of increasing amounts of the small molecule. Small-molecule inhibitors induced DNA damage and protein expressions of Ki-67 and γH2AX, and cleaved caspase-3 by inducing cell-cycle arrest. Activation of the DDR machinery, which if it does not repair RAD51-driven homologous recombination (HR), will trigger cell-cycle arrest, senescence, and apoptosis [46]. For example, breast cancer cells carrying mutations of the BRCA2 gene are deficient in the HR repair pathway and are consequently particularly sensitive to chemical inhibitors of alternative DNA repair pathways [47].

DNA DSBs are among the most toxic DNA lesions and can be generated by cancer chemotherapy [48]. Cellular responses to DNA damage upon DSB induction include activation of two protein kinase signaling pathways, ATM-CHK2 and ATR-CHK1 [49]. This process, is accompanied by p53-deficient cell progression through the S phase and is arrested by a DNA damage checkpoint in the G_2_ phase [50]. Interestingly, phosphorylation and activation of p53 following activation of the ATM/ATR induces G_2_/M arrest; specifically, p53 restrains CDC25c, a phosphatase that promotes mitosis, mainly by blocking activity of the cyclin B1/CDC2 complex [51, 52]. Upregulation of Bax protein levels results in formation of a heterodimer with an oncogene-derived protein (Bcl-2), thus increasing the opening of the mitochondrial voltage-dependent anion channel, which leads to loss of the membrane potential, induced by p53, which is further evidence of p53-mediated apoptosis [53, 54]. To identify the mechanisms, we sought out potential targets of this process in these cells. Our finding that CDC25c and cyclin B1/CDC2 were decreased in NSC745887-treated cells is in agreement with earlier results, in which DNA repair or cell-cycle arrest and apoptosis are responses after DNA damage. In contrast, our finding that CDC25a, cyclin A2/CDK2, and cyclin D1/CDK4/6 remained at functional levels after NSC745887 treatment demonstrates progression in the transition to the G_1_-S phase. Furthermore, Fas is a homotrimeric type II transmembrane protein present on cytotoxic T lymphocytes. It acts via trimerization of Fas receptors, which cross the membrane of the objective cell, and are essential for downstream events that disseminate the apoptotic signal [55]. DcR3 can be defined as an immunomodulator which is reported to interact with the FasL and is overexpressed in some malignant tumors [56]. Most importantly, the apoptotic effect of FasL/Fas signaling is obstructed by DcR3, a distinct secreted member of the tumor necrosis factor receptor superfamily that functions to prevent FasL/Fas interactions by competitively binding to the membrane-bound Fas and rendering them inactive by native glycosylation, which results in a reduced tendency to couple [56–58]. These data provided solid evidence that Fas forms a death-inducing signaling complex (DISC) upon ligand binding. This causes complete apoptosis and subsequent caspase-8 activation, which catalyzes the cleavage of the proapoptotic BH3-only protein, Bid, and discourages the Bcl-2 family from allowing Bax to be translocated to the outer mitochondrial membrane, thus permeabilizing it and facilitating release of proapoptotic proteins such as cytochrome c. These eventually lead to DNA degradation, membrane blebbing, and other hallmarks of apoptosis [59]. These data challenged the idea that DcR3 is extremely elevated in most patients with GBM and contributes to tumor cell evasion of host immune surveillance [38]. We found that DcR3 is expressed in untreated U118MG and U87MG cells, and was suppressed after NSC745887 treatment based on unchanged FasL levels. To our knowledge, this is the first direct comparison of a DcR3-expressing ensemble and mechanism of action of a small molecule to incorporate protein flexibility in structure-based drug design. Subsequently, these data argued in favor of activated caspase-8 promoting Bid upregulation and increasing the Bax/Bcl2 ratio, thus resulting in mitochondrial membrane depolarization, which is in agreement with FasL/Fas signaling. The data presented above conclusively point to the involvement of DDRs and apoptosis as important pathways to remediate DNA damage induced by NSC745887, so that it either directly alters DNA sequences or causes mutations. Further studies will examine our novel small-molecule inhibitor to delineate the structural requirements to further optimize its structure and control its polypharmacology.

## MATERIALS AND METHODS

### Cell lines, reagents, and test compounds

The synthesis of NSC745887 was described in our earlier study [9]. Chemicals used in this study were mostly purchased from Sigma-Aldrich (St. Louis, MO, USA). The human U87MG glioblastoma cell line was purchased from the Bioresource Collection Research Center (Taipei, Taiwan). U118MG cells were obtained from Dr. Dueng-Yuan Hueng (National Defense Medical Center, NDMC, Taipei, Taiwan). U118MG and U87MG cells were maintained in Dulbecco’s modified Eagle’s medium (DMEM) supplemented with 10% fetal bovine serum (FBS), 1% penicillin, and 1% streptomycin (Gibco/BRL, Grand Island, NY, USA). The medium was replaced with fresh complete medium 24 h before further experiments. All cell lines were maintained in a fully humidified incubator containing 5% CO_2_ at 37°C. The media and FBS were purchased from Mediatech (Atlanta Biologicals, Atlanta, GA, USA).

### Assay protocol

Cell viability was evaluated using a 3-(4,5-dimethylthiazol-2-yl)-2,5diphenyltetrazoliumbromide (MTT; Sigma, St. Louis, MO, USA) assay. Cells (4 × 10^4^) were calculated with a cell counter (Bio-Rad Laboratories, Hercules, CA, USA) plated in 24-well plates and incubated at 37°C for 24 h. Later, cells were grown with or without different concentrations of NSC745887, and cells were cultured to the indicated time points. Following this, cells in each well were treated with 500 µL of an MTT solution (5 mg/mL in phosphate-buffered saline (PBS)) and incubated for 4 h. Formazan crystals were solubilized in 500 µL DMSO, and optical densities were detected at a wavelength of 570 nm by a Synergy HT multi-detection microplate reader (Awareness Technology, Palm City, FL, USA). The relative survival rate was normalized to the untreated group and summarized for five independent experiments.

### Western blot analysis

After various treatments, glioma cells were lysed in ice-cold RIPA buffer (25 mM Tris-HCl at pH 7.6, 150 mM NaCl, 1.0% TritonX-100, 1.0% sodium deoxycholate, and 1% sodium dodecylsulfate (SDS)) containing protease and phosphatase inhibitors (GeneTex). Protein samples (100 µg per lane) were electrophoresed on 5% (for >300 kDa), 10% (for 40∼300 kDa), or 12% SDS polyacrylamide gels (for <40 kDa) and transferred to a 0.45-µM filter pore size hydrophobic Immobilon-P polyvinylidene fluoride (PVDF) membrane (Millipore). Strips from the membrane were blocked with blocking buffer (Genestar) at room temperature for 5 min and incubated overnight at 4°C with a 1:1000 dilution of rabbit antibodies against Ki-67, γH2AX, ATM, phosphorylated (p)-ATM, ATR, p-ATR, CHK1, p-CHK1, CHK2, p-CHK2, RAD51, p53, p-p53, CDC25a, CDC25c, cyclin A2, cyclin B1, cyclin D1, CDK2, CDK4, CDK6, CDC2, p-CDC2, Bcl2, Bax, DcR3, FasL, Bid, PARP, cleaved caspase-3, cleaved caspase-8, cleaved caspase-9, vinculin, β-actin, and α-tubulin. After washing, strips were incubated with a 1:10^4^ dilution of infrared (IR) dye-conjugated anti-rabbit immunoglobulin G (IgG) antibodies (LI-COR, Bioscience) in a dark room for 1 h. Then, the fluorescence density of the bands on the PVDF membrane was quantified by densitometry using Odyssey^®^ CLx Infrared Imaging System (LI-COR), taking the density of the control sample as 100% and expressing the density of the test sample relative to the expression of the internal control as a relative value.

### Flow cytometry-based apoptosis detection

A flow cytometric analysis was utilized to measure cell-cycle dynamics in different cell phases. In total, 2 × 10^5^ cells/well were seeded in six-well plates and incubated for 24 h. After application of NSC745887 for 24 or 48 h, cells were digested with 0.05% trypsin and gathered, and the prepared cell suspension was fixed with 75% ethanol at –20°C overnight. The cell suspension was washed with PBS and stained with 500 μL propidium iodide (PI)/RNase staining solution (BD Biosciences, Franklin Lakes, NJ, USA) for 15 min at room temperature in the dark. In total, 10^4^ stained cells were analyzed using the FACSVerse™ laser flow cytometric analysis system (BD Biosciences) for each sample. At least four independent experiments were conducted. Apoptosis assays were prepared by seeding 2 × 10^5^ U118MG or U87MG cells in six-well plates overnight, and growth medium either with or without different concentrations of NSC745887 was added for 24 or 48 h. An Annexin V-PE Dead Cell Apoptosis kit (BD Biosciences) was utilized for the apoptotic cell death analysis following the manufacturer’s protocol to prepare samples. Cells were trypsinized and washed twice with PBS, and pellets were resuspended in 100 μL of binding buffer, 5 µL Annexin V-PE, and 10 µL 7-AAD, mixed, and incubated for 15 min in the dark at room temperature. Next, 400 µL of binding buffer was added to cells, and 10^4^ events were acquired for each sample. 7-AAD was analyzed in a flow cytometer (FACSVerse™; BD Biosciences) at 488 nm, and Annexin V-PE fluorescence was detected at 617 nm. An APO-DIRECTTM Kit (BD Biosciences) was utilized for the DNA damage analysis, and a Flow Cytometry Mitochondrial Membrane Potential Detection Kit (BD Biosciences) was used to detect a normal Δψ in healthy mitochondria or a decreased Δψ in mitochondria of apoptotic cells. Each experiment was conducted at least three times. Following acquisition, data were analyzed using Flow Jo vers. 7.6.5 software (Tree Star, Ashland, OR, USA). In total, 10^4^ cells were analyzed for each sample.

### Mouse xenograft model and positron emission tomographic (PET) scan analysis

All protocols were authorized by the Institutional Animal Care and Use Committee of the NDMC (approval no.: IACUC16-075, Taipei, Taiwan). Female BALB/cAnN.Cg-Foxn1nu/CrlNarl mice (8 weeks old; 20∼22 g) were acquired from the National Laboratory Animal Center (Taipei, Taiwan) and were free from contamination as confirmed by health reports. Following anesthetization with isoflurane, 10^6^ U118MG cells were subcutaneously inoculated, and tumors grew up to 50 mm^3^. Mice bearing gliomas were treated with 5 mg/kg/day NSC745887 via an intraperitoneal (i.p.) injection, and an equal amount of DMSO was administered to the control group. To evaluate the NSC745887 treatment effect on tumors, BALB/c nude mice were monitored using [^18^F]-2-deoxy-2-fluoro-D-glucose ([^18^F]-FDG)/animal-PET) scanning on days 0, 7, 14, 21, and 28 after treatment. The complete imaging procedure was carried out in the Laboratory Animal Center of the NDMC, which is certified by the Association for Assessment and Accreditation of Laboratory Animal Care International (AAALAC 2007). BALB/c nude mice of both groups (*n* = 6) were i.p.-injected with 285∼295 µCi (9.5∼10.5 MBq) of [^18^F]-FDG after overnight starvation. After allowing the [^18^F]-FDG injection to be distributed for 15 min, mice were anesthetized, and were imaged for 20 min using animal-PET statistical scanning with BIOPET105 (Bioscan, Washington DC, USA) with the energy window set to 250∼700 keV. Three-dimensional (3D)-ordered subset expectation maxi-mization (OSEM) was used for image reconstruction and AMIDE software (vers. 1.0.4) for image data analysis. The tumor volume was quantitated by estimating the standard uptake value (SUV) of [^18^F]-FDG, which indicates the level of [^18^F]-FDG in a volume of interest (VOI) (representative of a focal tumor) relative to average [^18^F]-FDG levels in the whole body. The experiment lasted 28 days; then, all of the mice were euthanized and fixed in 4% paraformaldehyde on day 29. Tumor tissues were collected and weighed, and vital organs including the heart, kidneys, and liver were extracted for hematoxylin and eosin (H&E) and immunohistochemical (IHC) staining.

### Histological and IHC evaluations

Xenograft tumors and vital organs were fixed in 4% paraformaldehyde at 4°C for 48 h. Tissues were embedded in a standard tissue-freezing medium (O.C.T. compound) and sliced into 4-μm-thick sections. Standard H&E staining was carried out for a histomorphological evaluation of the heart, kidneys, and liver. Expressions of Ki-67 (#9027, Cell Signaling), γH2AX (#9718, Cell Signaling), and cleaved caspase-3 (#9661, Cell Signaling) in tumors of the mice were detected with an IHC analysis, and were observed in 10 random fields for each group.

### Data analysis

All experiments were performed at least three times, and values are reported as the mean ± standard deviation (SD). Differences between groups were evaluated using the Kruskal-Wallis test followed by post-hoc comparisons with GraphPad Prime 5.0 software. Details of each statistical analysis used are recorded in the figure legends. Statistical significance was set to *p* < 0.05.

## CONCLUSIONS

We provide preclinical evidence for NSC745887, with a tetraheterocyclic motif, as a potential new agent for treating GBM. We showed that NSC745887 treatment induced DDR in GBM cells. This is consistent with an earlier model of p53 in regulating DNA damage caused by NSC745887, which invoked participation of a tetraheterocyclic system in its DNA-damaging effects and exhibited DNA fragmentation, cell cycle arrest, MMP changes, and an apoptosis-mediated signaling pathway. Our data are consistent with findings of the role of DcR3 in glioma progression [5]; it was reported to protect malignant gliomas from their functional and might be an interesting small molecule for DcR3 in drug design. This finding provides an explanation of the anticancer activity of NSC745887, which was hitherto unknown. We envision that the data presented herein can lay the foundation for evaluating NSC745887 as a novel anti-glioblastoma agent. To this end, the assays we report are likely to enable the discovery of novel anti-glioblastoma agents and will help advance the translational development of new biological targets in these clinically important pathways.

## SUPPLEMENTARY MATERIALS FIGURES AND TABLES







## Abbreviations

Animal-PET: animal positron emission tomography
ATM: ataxia-telangiectasia mutated
ATR: ATM and Rad3-related
BCNU: carmustine
CCNU: lomustine
DcR3: decoy receptor 3
DDR: DNA damage response
DMEM: Dulbecco’s modified Eagle’s medium
DMSO: dimethyl sulfoxide
FasL: fatty acid synthetase ligand
FBS: fetal bovine serum
[^18^F]-FDG: [^18^F]-fluorodeoxyglucose
GBM: glioblastoma multiforme
IgG: immunoglobulin G
IHC: immunohistochemical
i.p: intraperitoneal
IR: infrared
MTT: 3-(4,5-dimethylthiazol-2-yl)-2,5 diphenyltetrazoliumbromide
NCI: National Cancer Institute
OSEM: ordered-subset expectation maximization
PARP: poly(ADP-ribose) polymerase
PBS: phosphate-buffered saline
PI: propidium iodide
PVDF: polyvinylidene difluoride
SDS: sodium dodecylsulfate
TEA: Trimethylamine
THF: tetrahydrofuran
TMZ: temozolomide
