# Supplementary material for: Identification and preclinical evaluation of the small molecule, NSC745887, for treating glioblastomas via suppressing DcR3-associated signaling pathways
Source: Oncotarget. 2017 Dec 27;9(15):11922–37. doi: 10.18632/oncotarget.23714 (PMC5844718; doi:10.18632/oncotarget.23714)
Supplement: Supplementary file 1 [file oncotarget-09-11922-s001.pdf]

Identification and preclinical evaluation of the small molecule, NSC745887, for treating glioblastomas via suppressing DcR3-associated signaling pathways

SUPPLEMENTARY MATERIALS

Naphtho[2,3-*f*]quinoxaline-7,12-dione (D-1)

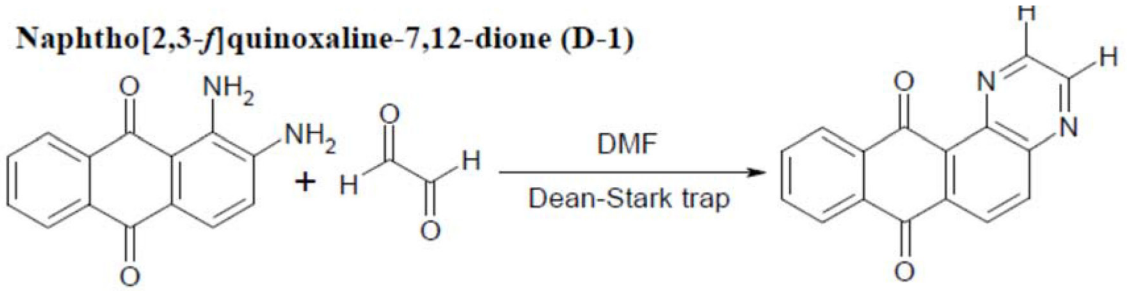

Mol. Wt. : 260.0586 ($C_{16}H_8N_2O_2$)

R_f : 0.45 (ethyl acetate: dichloromethane = 1: 4)

Yield : 23%

mp : 270-272℃ (EtOH)

IR (KBr) cm^{-1} : 3413 (NH), 3365 (NH), 1626 (CO)

EI-MS m/z : 150 (54%), 238 (73%), 260 (M^+, 100%)
HRMS (ESI-TOF) m/z :
calcd for $C_{16}H_9N_2O_2^+$ $[M+H]^+$: 261.0659, found: 261.0663

^{1}H-NMR (300 MHz, $CDCl_3$) δ (ppm) :
7.82-7.87 (2H, m, Ar-$H_{8,11}$), 8.29-8.36 (2H, m, Ar-$H_{9,10}$),
8.48 (1H, d, J=8.7Hz, Ar-H_5), 8.72 (1H, d, J=8.7Hz, Ar-H_6),
8.99 (1H, d, J=1.5Hz, -N=CH-), 9.25 (1H, d, J=1.5Hz,-CH=N-)

^{13}C-NMR (75MHz, $CDCl_3$) δ (ppm) :
126.72, 127.05, 127.46, 130.05, 131.98, 133.77, 134.76, 135.18, 135.88, 135.93, 136.03, 145.42, 146.40, 147.77, 183.21 (CO), 183.61 (CO)

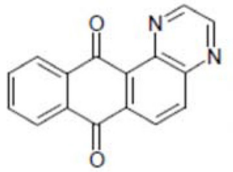

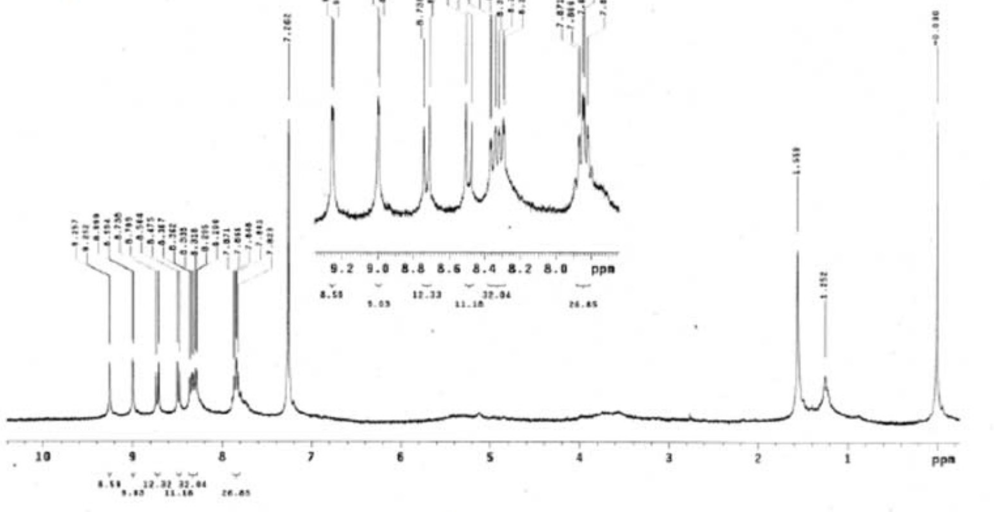

D1 之 ^{1}H-NMR 圖譜

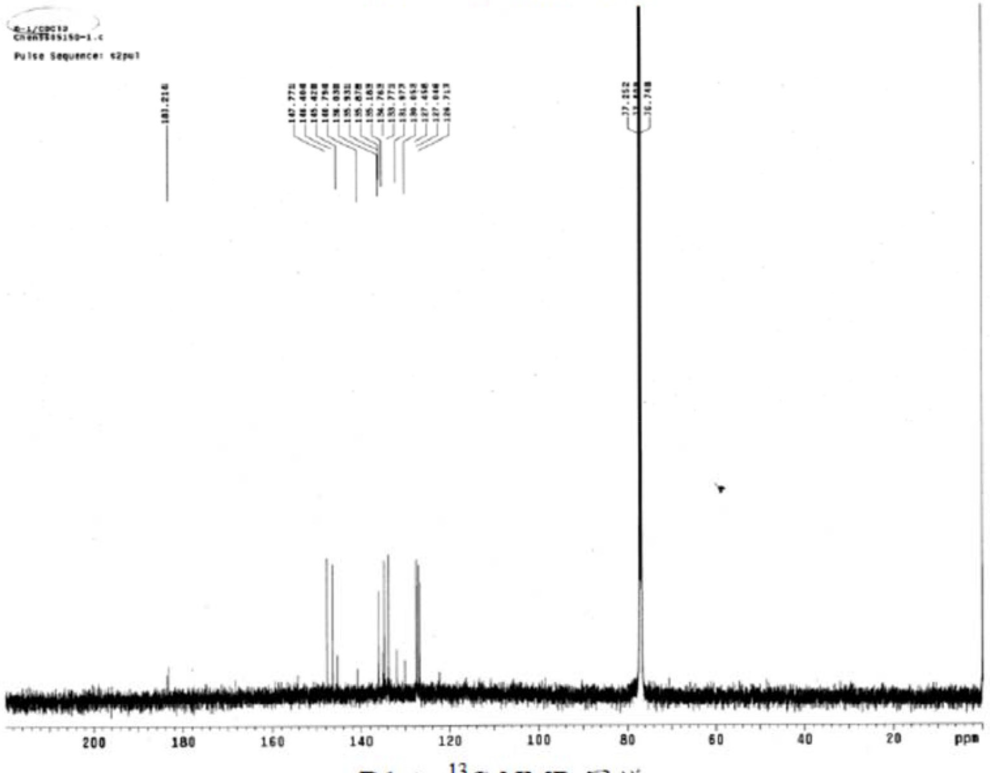

D1 之 ^{13}C-NMR 圖譜

Supplementary Figure 1: Synthesis of NSC745887.

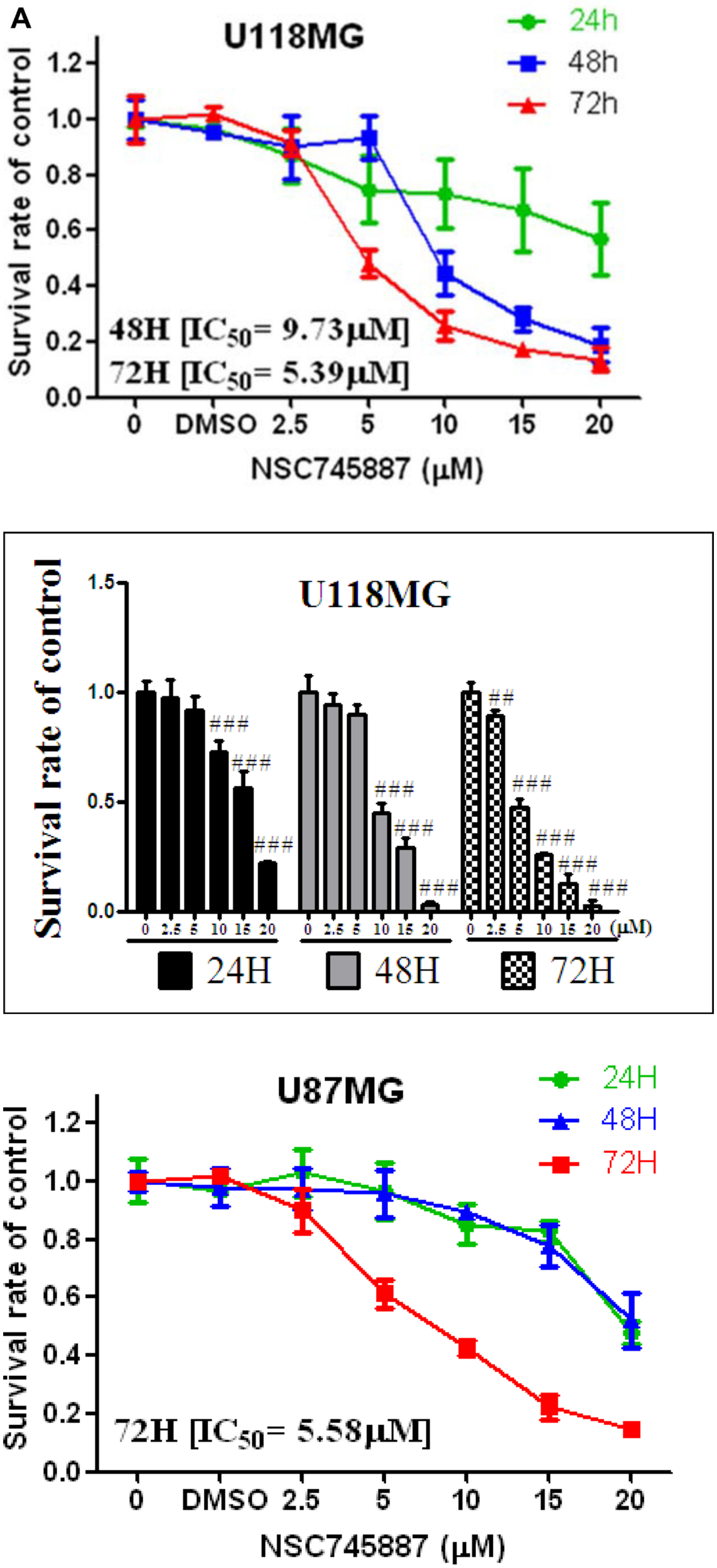

A
U118MG
24h
48h
72h
Survival rate of control
1.2
1.0
0.8
0.6
0.4
0.2
0.0
48H [IC50= 9.73μM]
72H [IC50= 5.39μM]
0
DMSO
2.5
5
10
15
20
NSC745887 (μM)
U118MG
Survival rate of control
1.5
1.0
0.5
0.0
###
##
0 2.5 5 10 15 20 (μM)
24H
48H
72H
U87MG
24H
48H
72H
Survival rate of control
72H [IC50= 5.58μM]
0
DMSO
2.5
5
10
15
20
NSC745887 (μM)

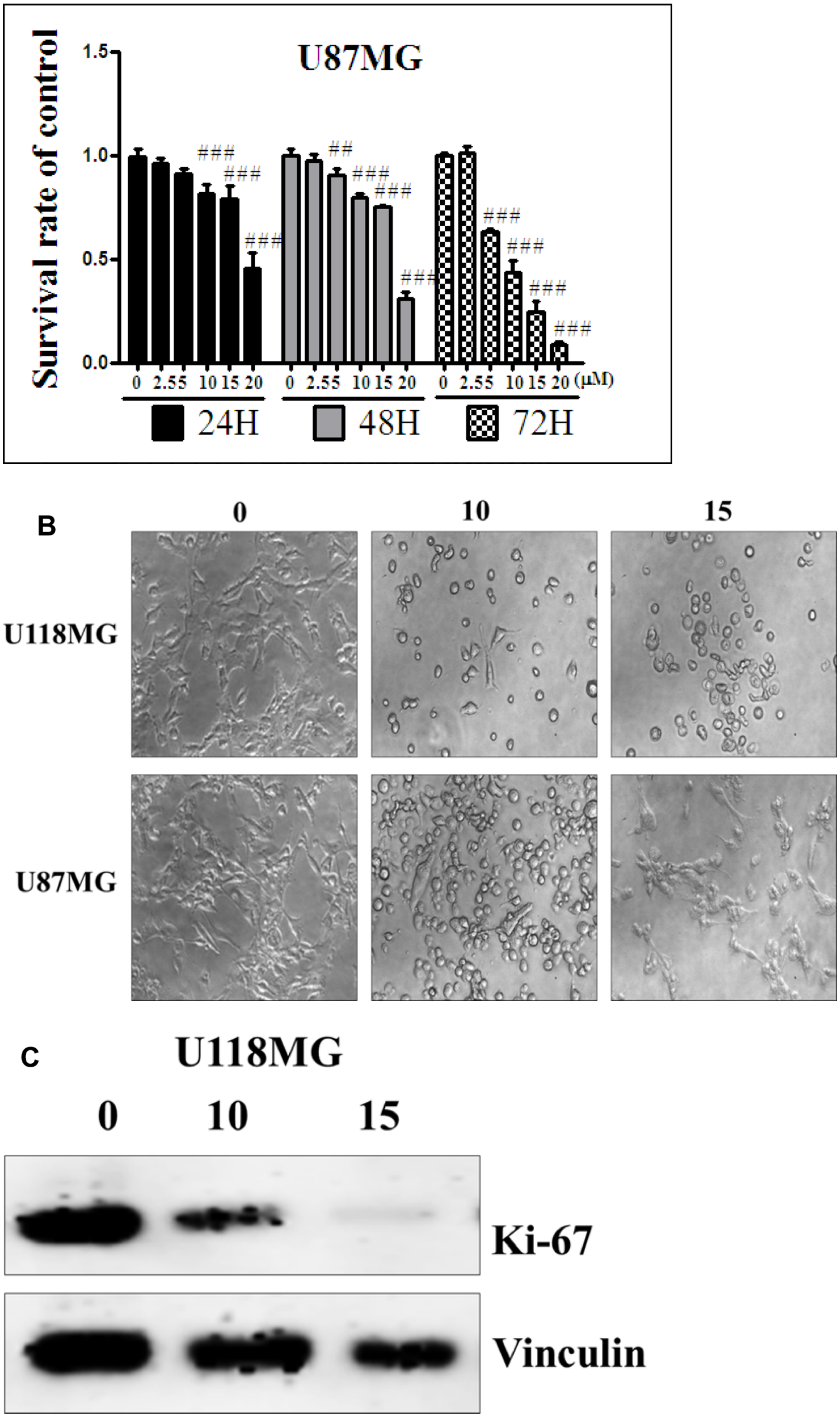
U87MG
Survival rate of control
1.5
1.0
0.5
0.0
###
##
0 2.55 10 15 20
(μM)
24H
48H
72H
B
0
10
15
U118MG
U87MG
C
U118MG
0
10
15
Ki-67
Vinculin

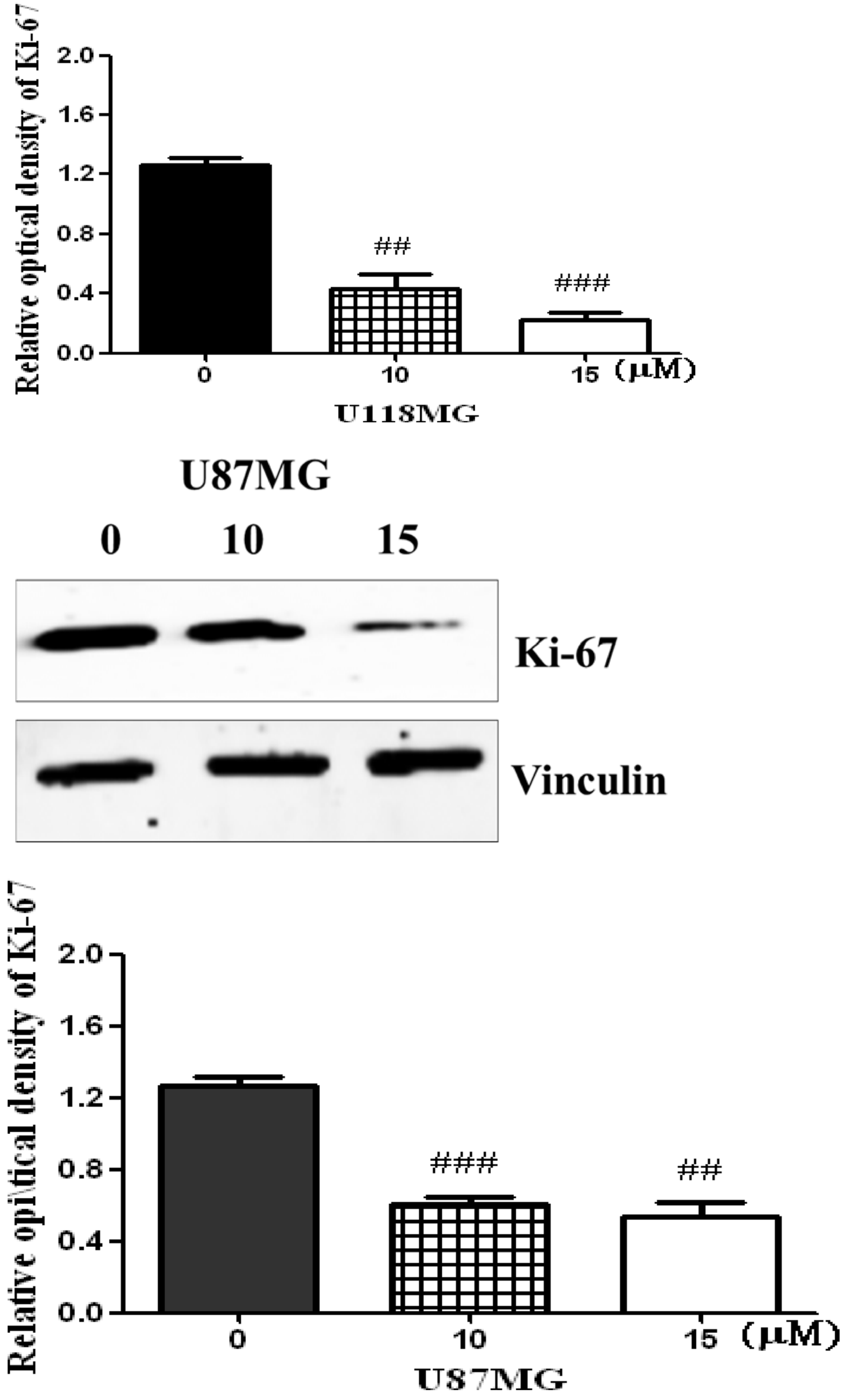

Supplementary Figure 2: (**A**) U118MG and U87MG cells were treated with NSC745887 [0, 2.5, 5, 10, 15 and 20 μM] for 24 h, 48 h and 72 h, respectively. The cells were subsequently subjected to a time-course assay. The cells were detected every 24 h and the cell survival rate was analyzed by MTT assay. U118MG cells were observed to be the most sensitive cell line to NSC745887, as the percentage of apoptosis increased already 24 h after NSC745887 treatment. The U87MG cells responded more slowly, and started to undergo less apoptosis about 24 h after the treatment. (**B**) The observation under microscope also showed that NSC745887-treated cells later 24 h had shrinking morphologies and lower densities, a certain emblem of cell death. (**C**) The typical biomarker of cell proliferation in the two GBM cell lines was determined by using Western blotting. Moreover, Western blotting of the expression of Ki-67, suggesting that NSC745887 decreased cell proliferation. Data are presented as mean ± SD, statistically signification values of $^{\#\#}P < 0.01$, $^{\#\#\#}P < 0.001$ compared with the control group.

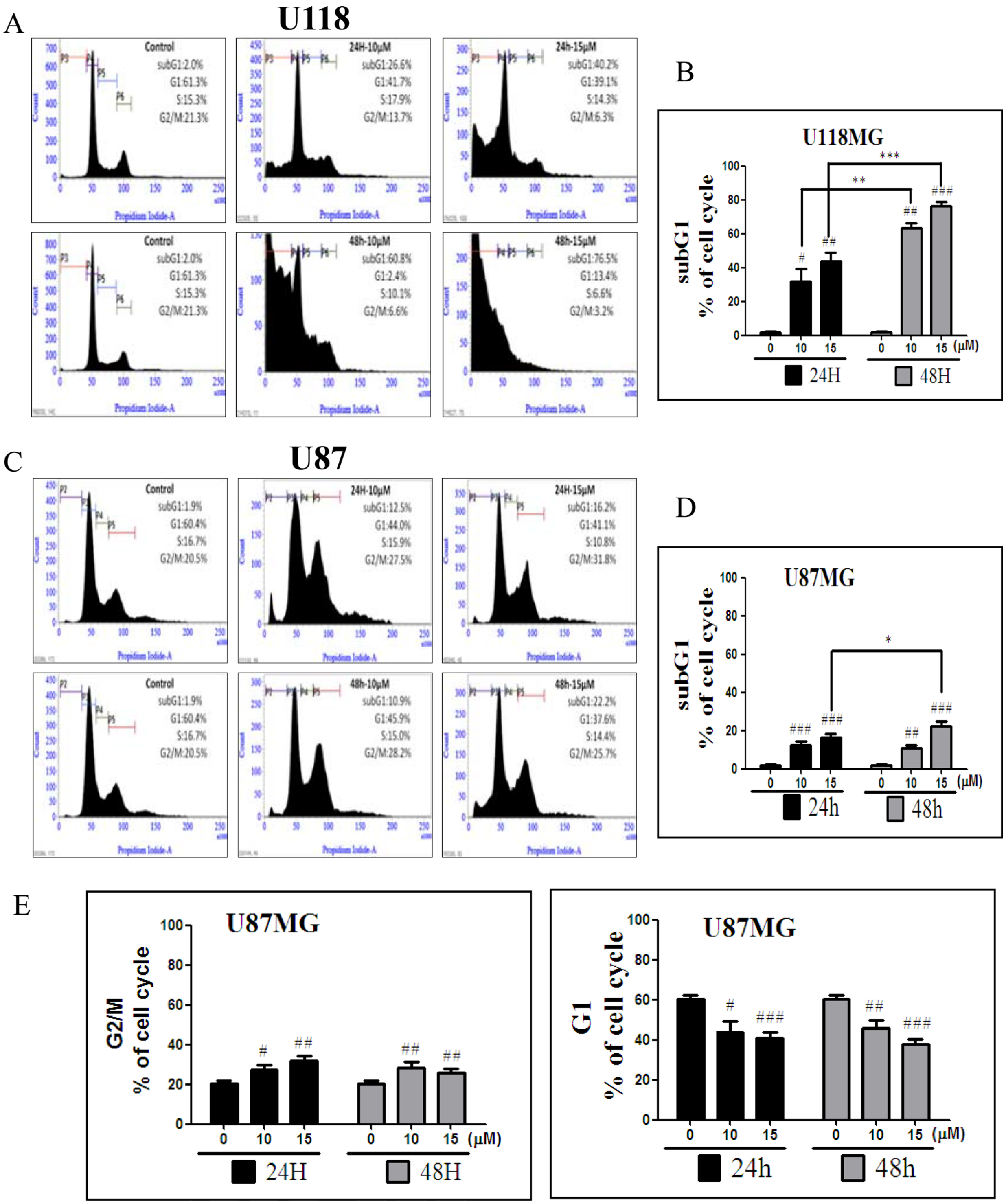
A
U118
Control
subG1:2.0%
G1:61.3%
S:15.3%
G2/M:21.3%
24H-10μM
subG1:26.6%
G1:41.7%
S:17.9%
G2/M:13.7%
24h-15μM
subG1:40.2%
G1:39.1%
S:14.3%
G2/M:6.3%
48h-10μM
subG1:60.8%
G1:2.4%
S:10.1%
G2/M:6.6%
48h-15μM
subG1:76.5%
G1:13.4%
S:6.6%
G2/M:3.2%
Propidium Iodide-A
Count
B
U118MG
subG1
% of cell cycle
24H
48H
(μM)
C
U87
Control
subG1:1.9%
G1:60.4%
S:16.7%
G2/M:20.5%
24H-10μM
subG1:12.5%
G1:44.0%
S:15.9%
G2/M:27.5%
24H-15μM
subG1:16.2%
G1:41.1%
S:10.8%
G2/M:31.8%
48h-10μM
subG1:10.9%
G1:45.9%
S:15.0%
G2/M:28.2%
48h-15μM
subG1:22.2%
G1:37.6%
S:14.4%
G2/M:25.7%
D
U87MG
subG1
% of cell cycle
24h
48h
(μM)
E
U87MG
G2/M
% of cell cycle
24H
48H
U87MG
G1
% of cell cycle
24h
48h
(μM)

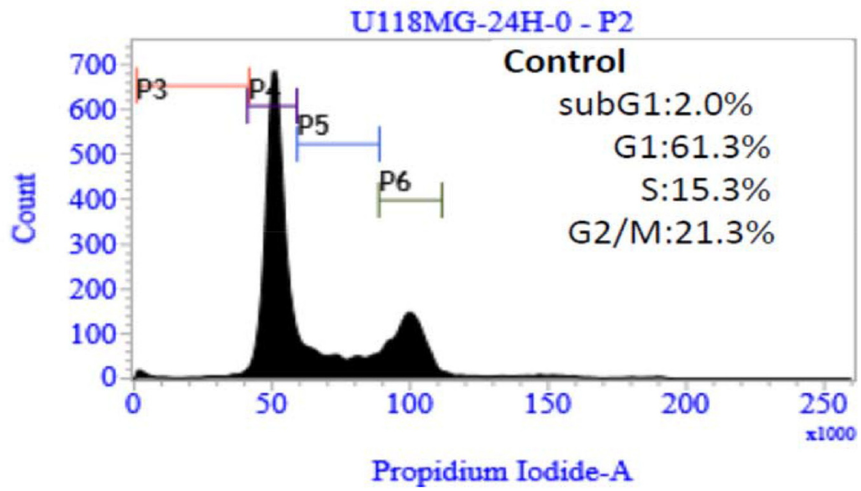
U118MG-24H-0 - P2
Control
subG1:2.0%
G1:61.3%
S:15.3%
G2/M:21.3%
P3
P4
P5
P6
Count
Propidium Iodide-A
x1000

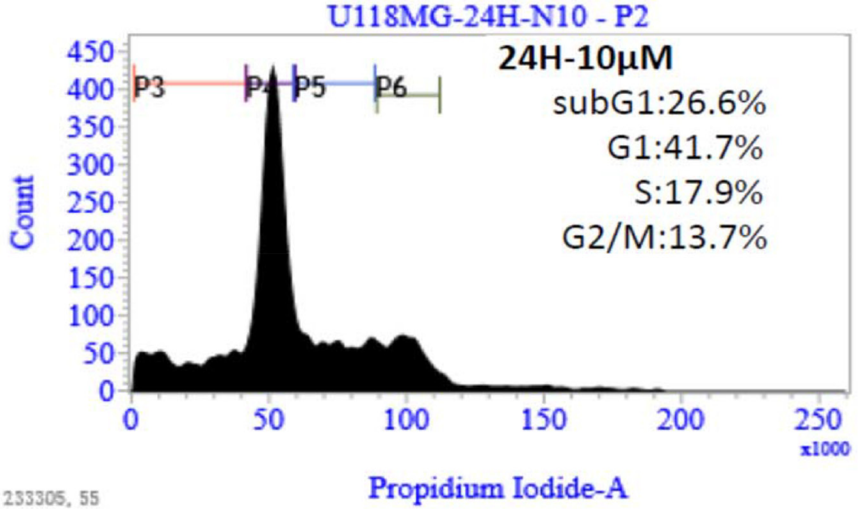
U118MG-24H-N10 - P2
24H-10μM
subG1:26.6%
G1:41.7%
S:17.9%
G2/M:13.7%
P3
P4
P5
P6
Count
Propidium Iodide-A
x1000
233305, 55

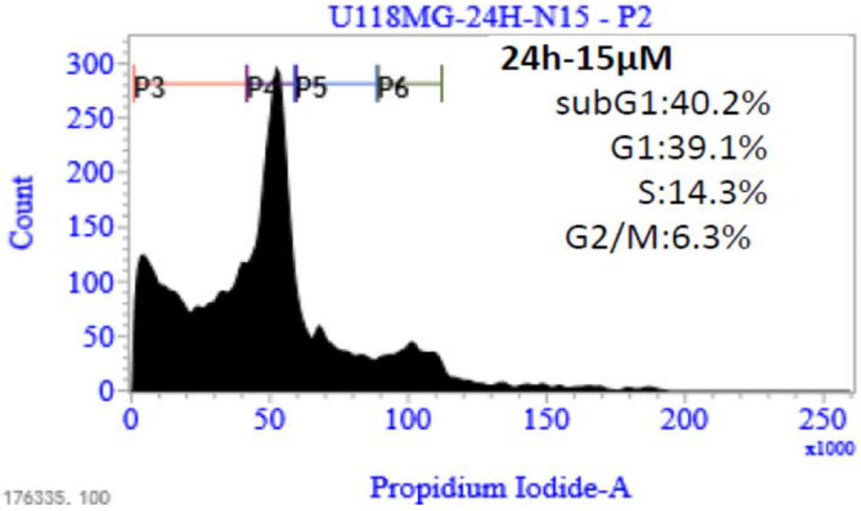
U118MG-24H-N15 - P2
24h-15μM
subG1:40.2%
G1:39.1%
S:14.3%
G2/M:6.3%
P3
P4
P5
P6
Count
Propidium Iodide-A
x1000
176335, 100

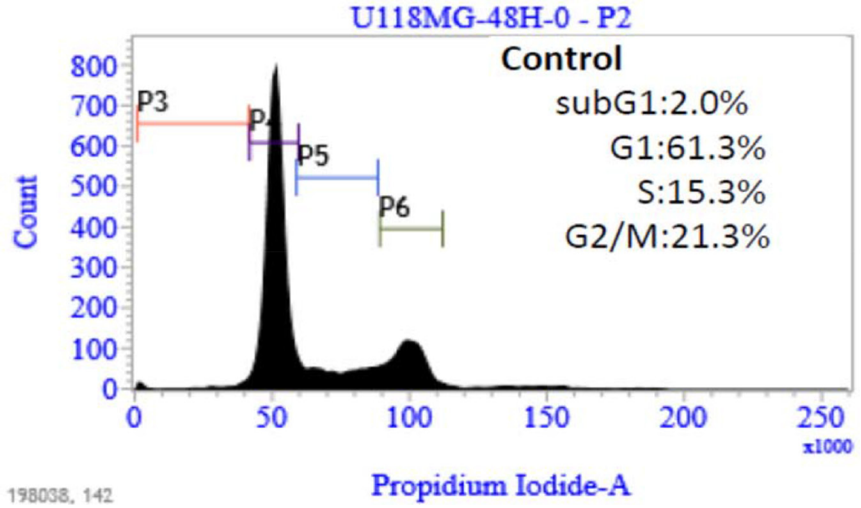
U118MG-48H-0 - P2
Control
subG1:2.0%
G1:61.3%
S:15.3%
G2/M:21.3%
P3
P4
P5
P6
Count
Propidium Iodide-A
x1000
198038, 142

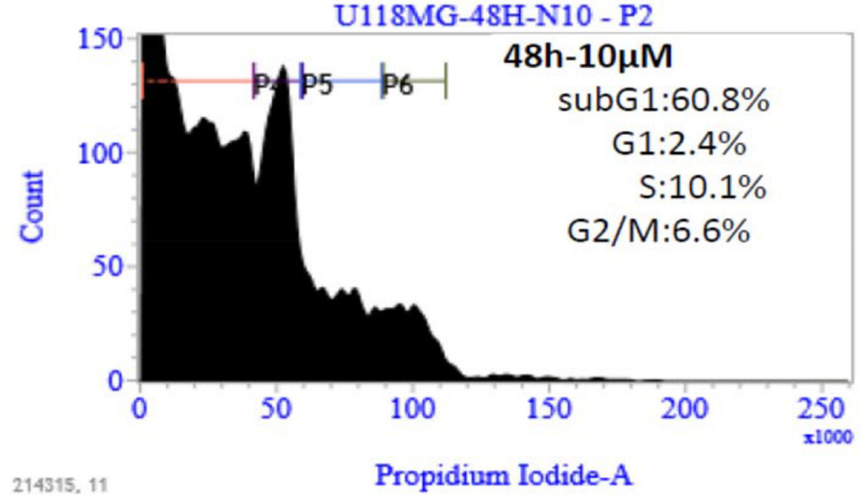
U118MG-48H-N10 - P2
48h-10μM
subG1:60.8%
G1:2.4%
S:10.1%
G2/M:6.6%
P4
P5
P6
Count
Propidium Iodide-A
x1000
214315, 11

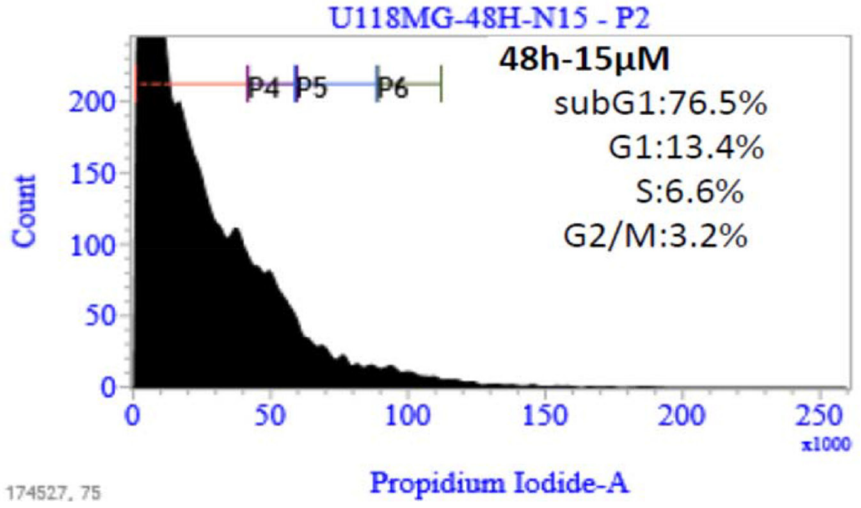
U118MG-48H-N15 - P2
48h-15μM
subG1:76.5%
G1:13.4%
S:6.6%
G2/M:3.2%
P4
P5
P6
Count
Propidium Iodide-A
x1000
174527, 75

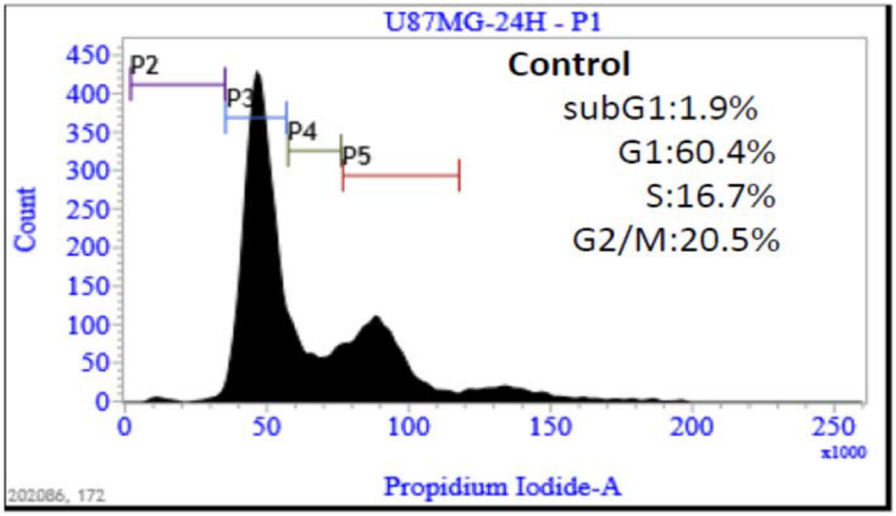
U87MG-24H - P1
Control
subG1:1.9%
G1:60.4%
S:16.7%
G2/M:20.5%
P2
P3
P4
P5
Count
Propidium Iodide-A
x1000
202086, 172

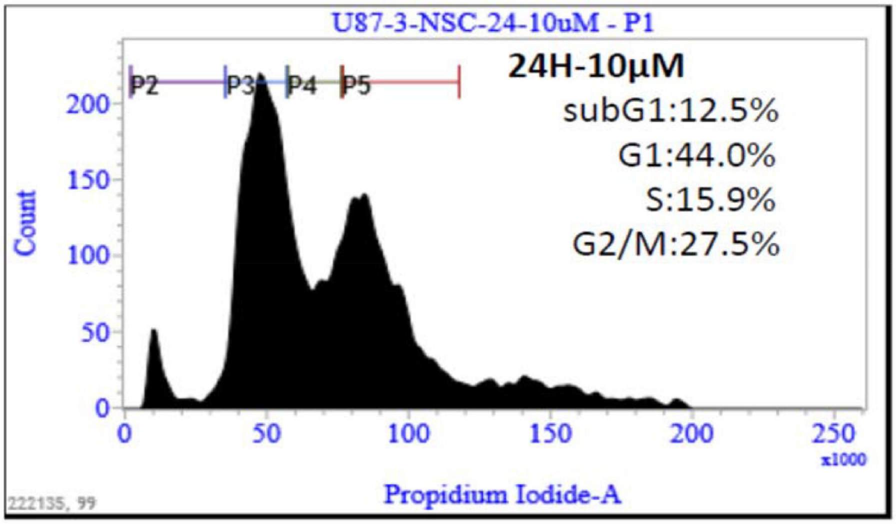
U87-3-NSC-24-10uM - P1
24H-10μM
subG1:12.5%
G1:44.0%
S:15.9%
G2/M:27.5%
P2
P3
P4
P5
Count
Propidium Iodide-A
x1000
222135, 99

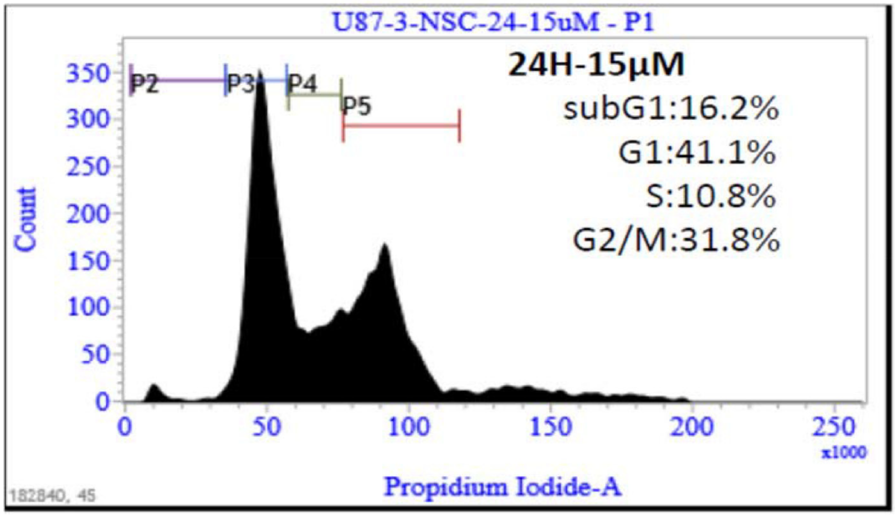
U87-3-NSC-24-15uM - P1
24H-15μM
subG1:16.2%
G1:41.1%
S:10.8%
G2/M:31.8%
P2
P3
P4
P5
Count
Propidium Iodide-A
x1000
182840, 45

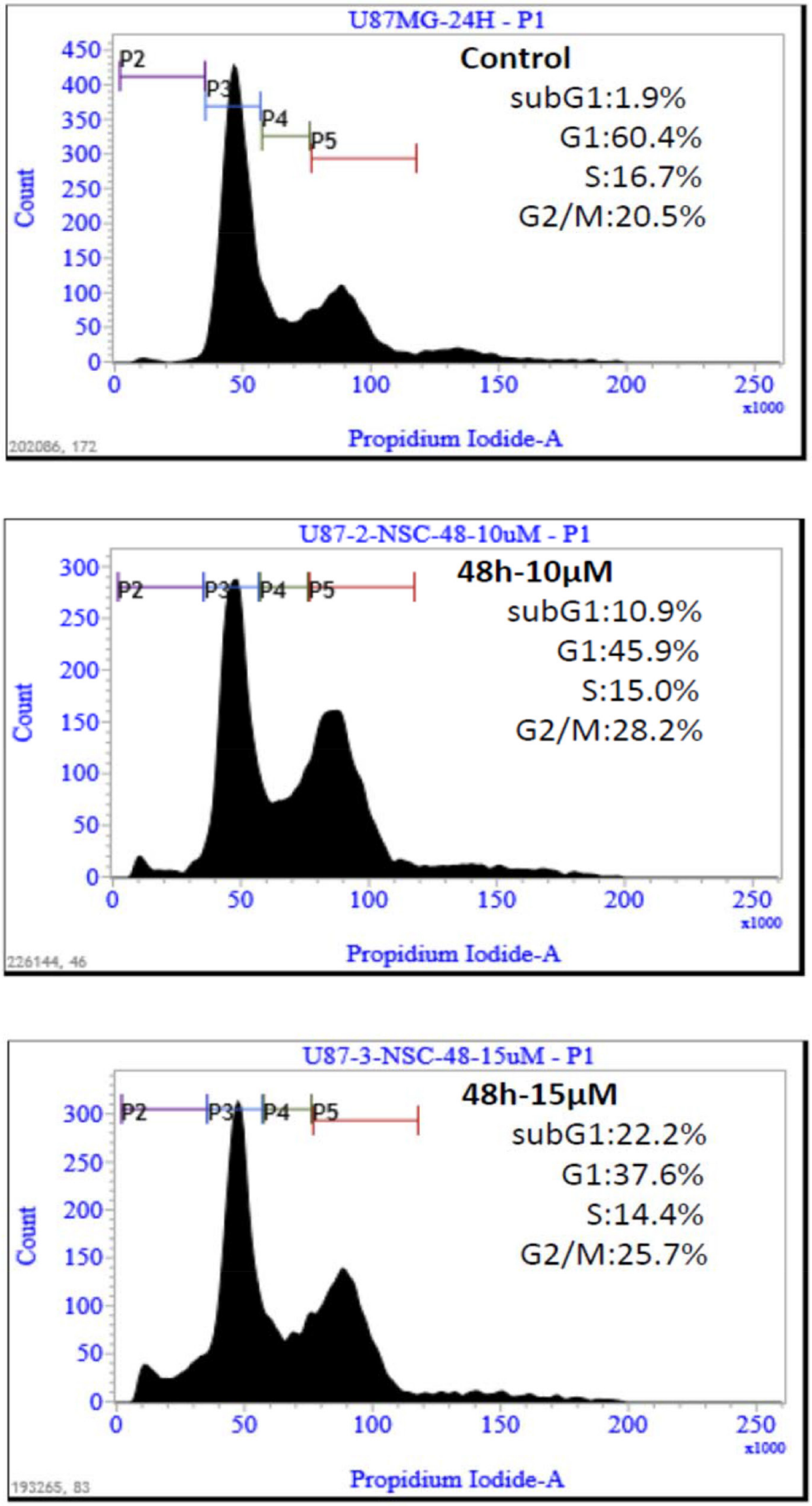

Supplementary Figure 3: Regulation of the cell cycle by NSC745887 treatment of U118MG and U87MG cells. (**A**) Cell-cycle phase distributions were analyzed with increasing doses of NSC745887 (10 and 15 μM) for 24 and 48 h, and representations of the cell-cycle modes of U118MG cells. (**C**) U87MG cells are shown. (**B**, **D**) Quantitative analyses of U118MG and U87MG cell populations in the sub-G1 phase using BD FACSuite analytical software. (**E**) Quantitative analyses of U87MG cell population in the G2/M and G1 phases. Data are presented as the mean ± SD; statistical significance is indicated by $^{\#}p < 0.05$, $^{\#\#}p < 0.01$, $^{\#\#\#}p < 0.001$ compared to the control group. $^{*}p < 0.05$, $^{**}p < 0.01$, $^{***}p < 0.001$, compared to the same concentration at 24 and 48 h.

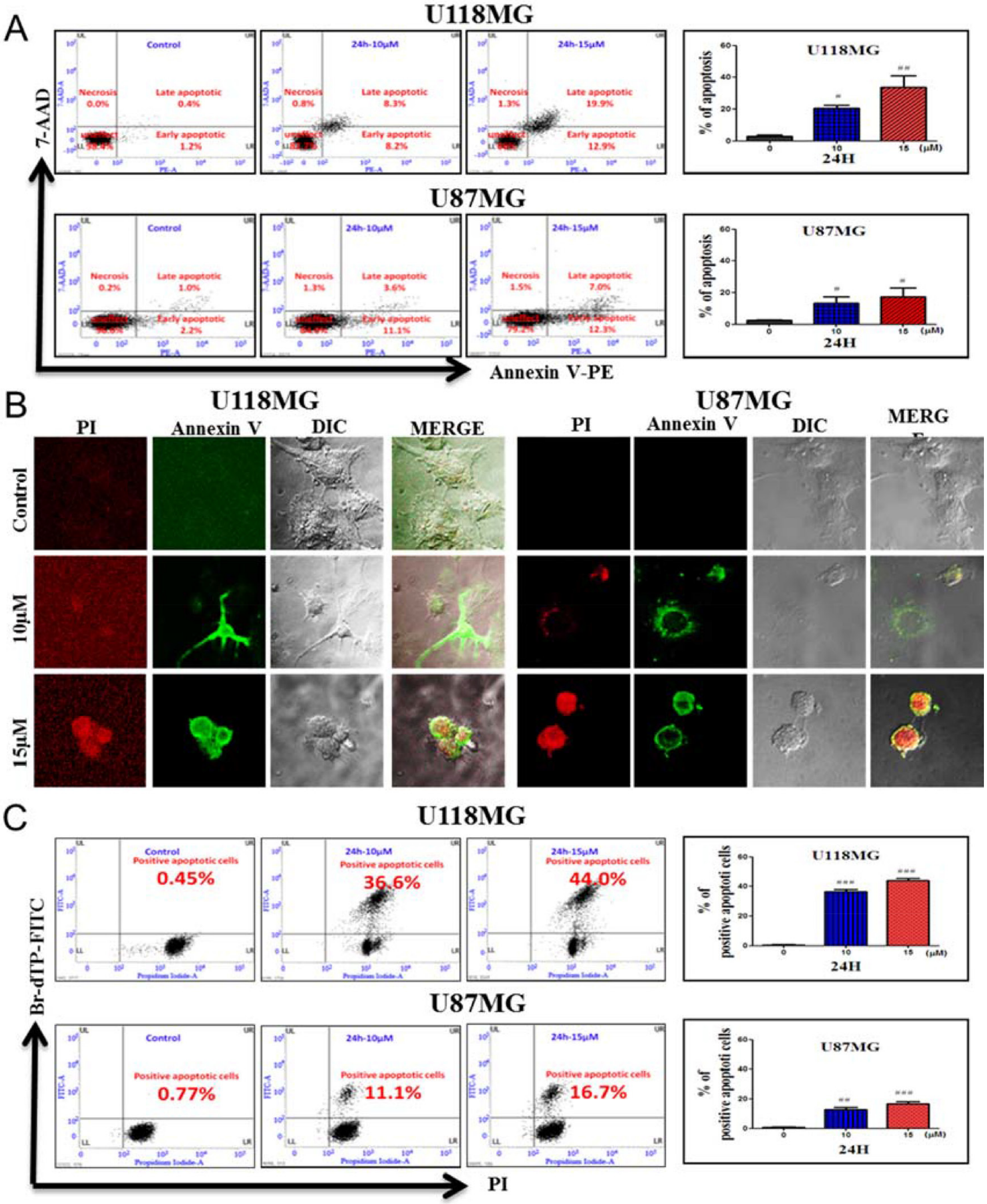
A
U118MG
Control
24h-10μM
24h-15μM
Necrosis
Late apoptotic
Early apoptotic
7-AAD
% of apoptosis
24H
U87MG
Annexin V-PE
B
PI
Annexin V
DIC
MERGE
Control
10μM
15μM
C
Positive apoptotic cells
0.45%
36.6%
44.0%
0.77%
11.1%
16.7%
Br-dTP-FITC
% of positive apoptoti cells
PI

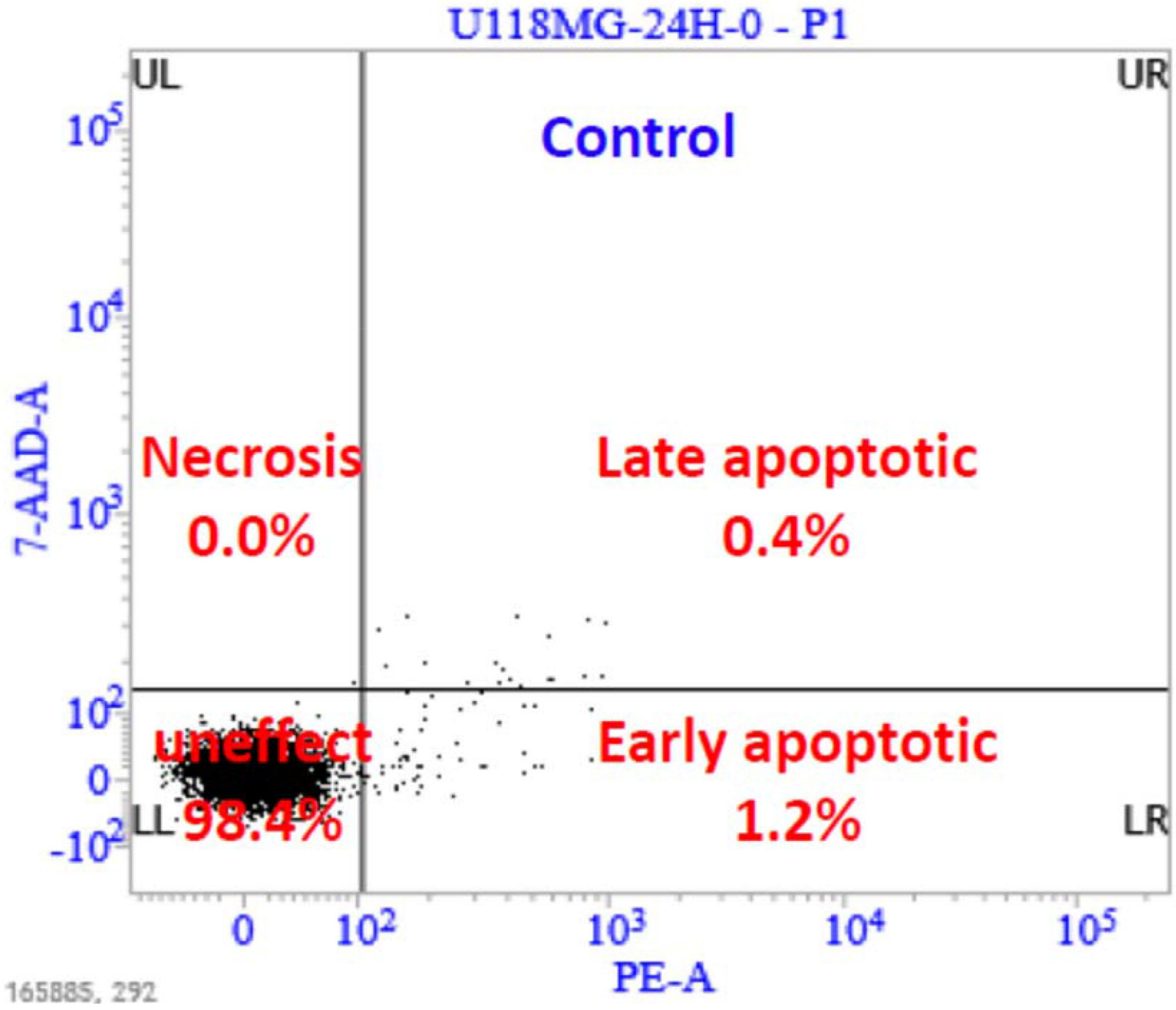
U118MG-24H-0 - P1
UL
UR
Control
Necrosis
0.0%
Late apoptotic
0.4%
uneffect
98.4%
Early apoptotic
1.2%
LL
LR
7-AAD-A
PE-A
165885, 292

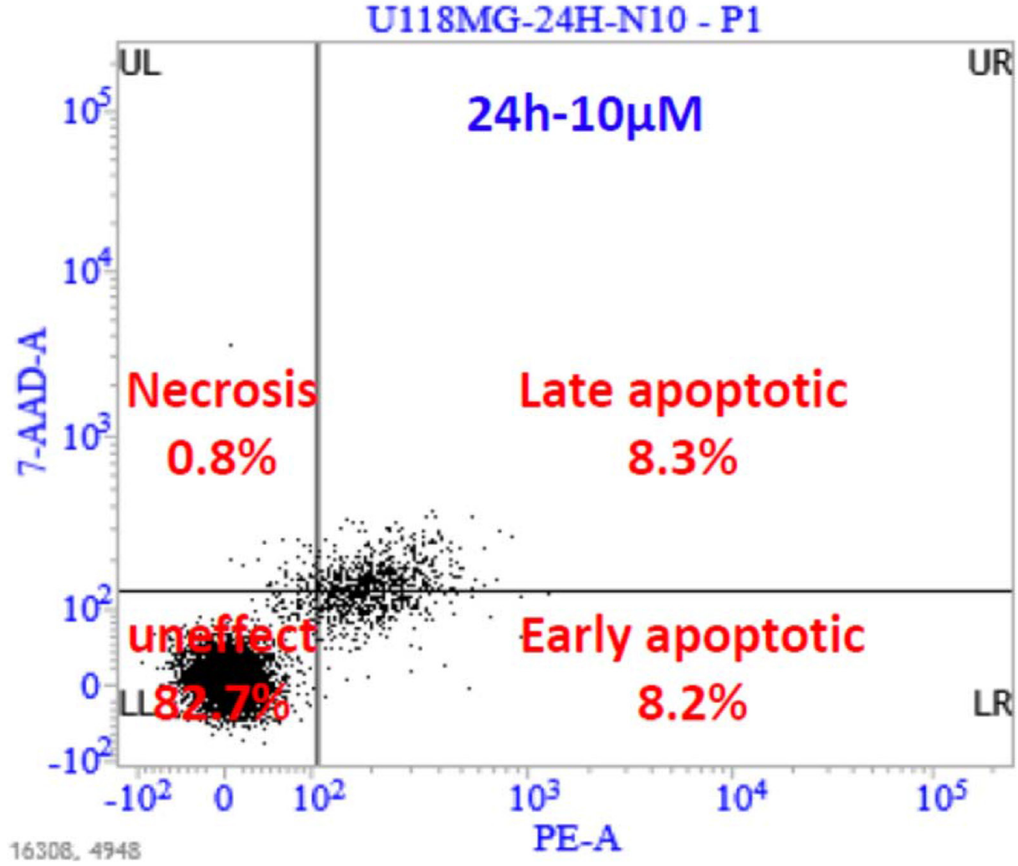
U118MG-24H-N10 - P1
24h-10μM
UL
UR
LL
LR
Necrosis
0.8%
Late apoptotic
8.3%
uneffect
82.7%
Early apoptotic
8.2%
7-AAD-A
PE-A
16308, 4948

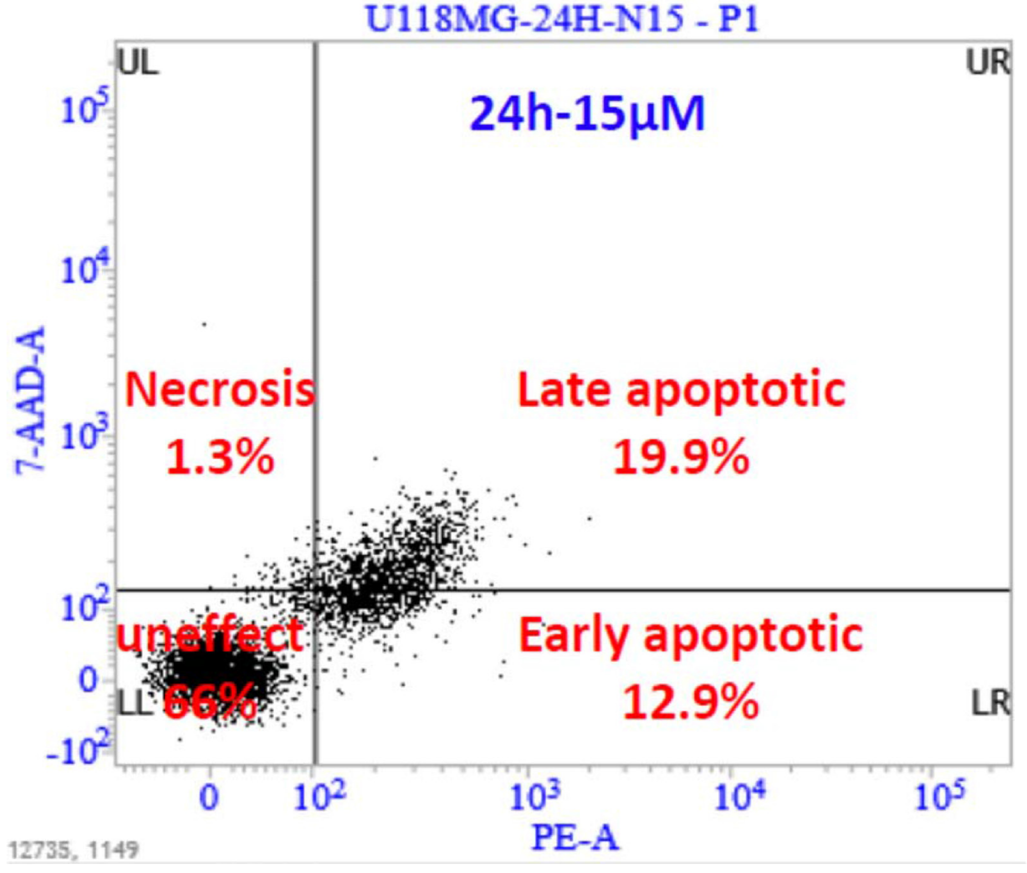
U118MG-24H-N15 - P1
24h-15μM
UL
UR
LL
LR
Necrosis
1.3%
Late apoptotic
19.9%
uneffect
66%
Early apoptotic
12.9%
7-AAD-A
PE-A
12735, 1149

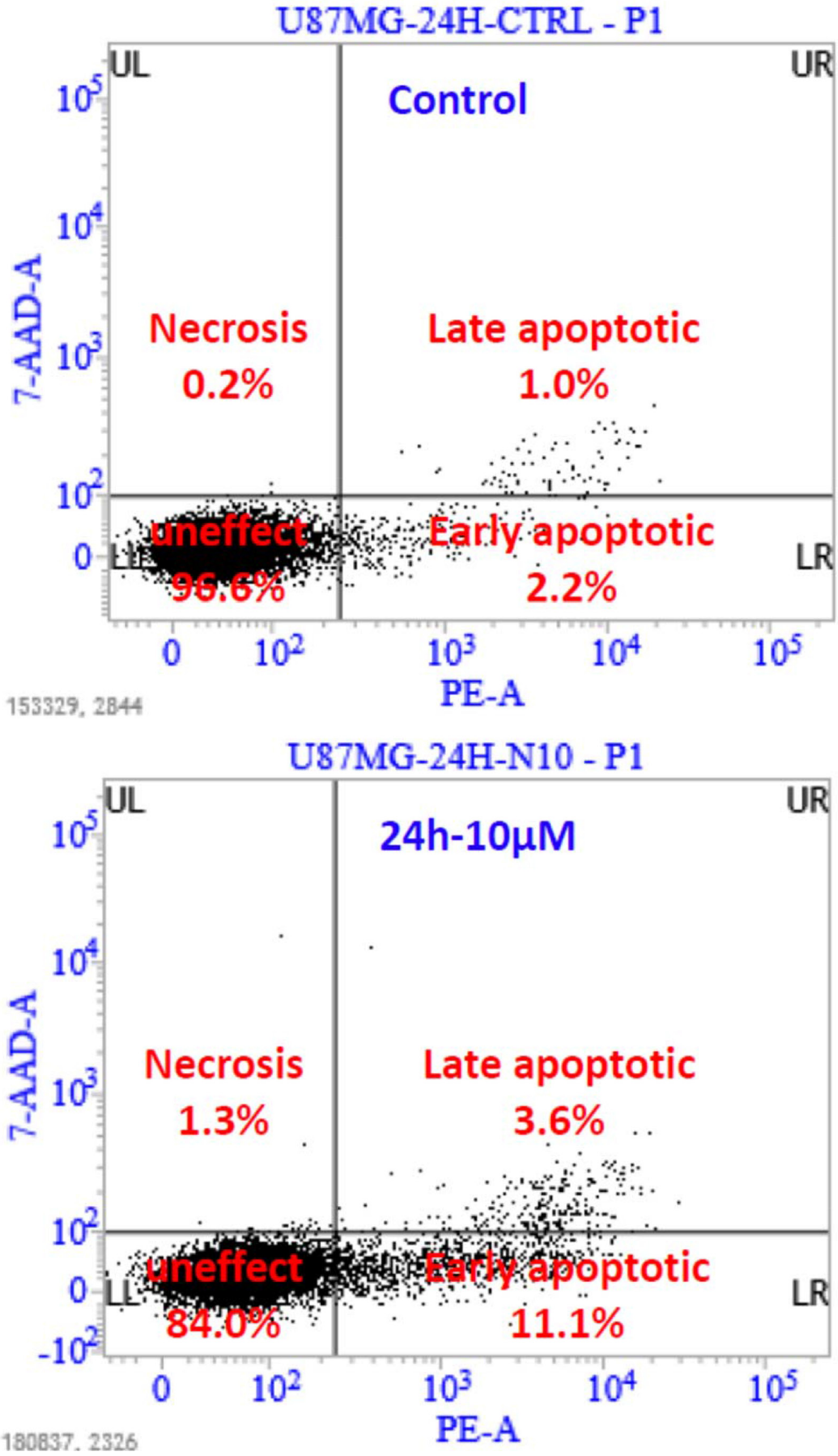
U87MG-24H-CTRL - P1
UL
UR
Control
7-AAD-A
Necrosis
0.2%
Late apoptotic
1.0%
uneffect
96.6%
Early apoptotic
2.2%
LL
LR
PE-A
153329, 2844
U87MG-24H-N10 - P1
UL
UR
24h-10μM
7-AAD-A
Necrosis
1.3%
Late apoptotic
3.6%
uneffect
84.0%
Early apoptotic
11.1%
LL
LR
PE-A
180837, 2326

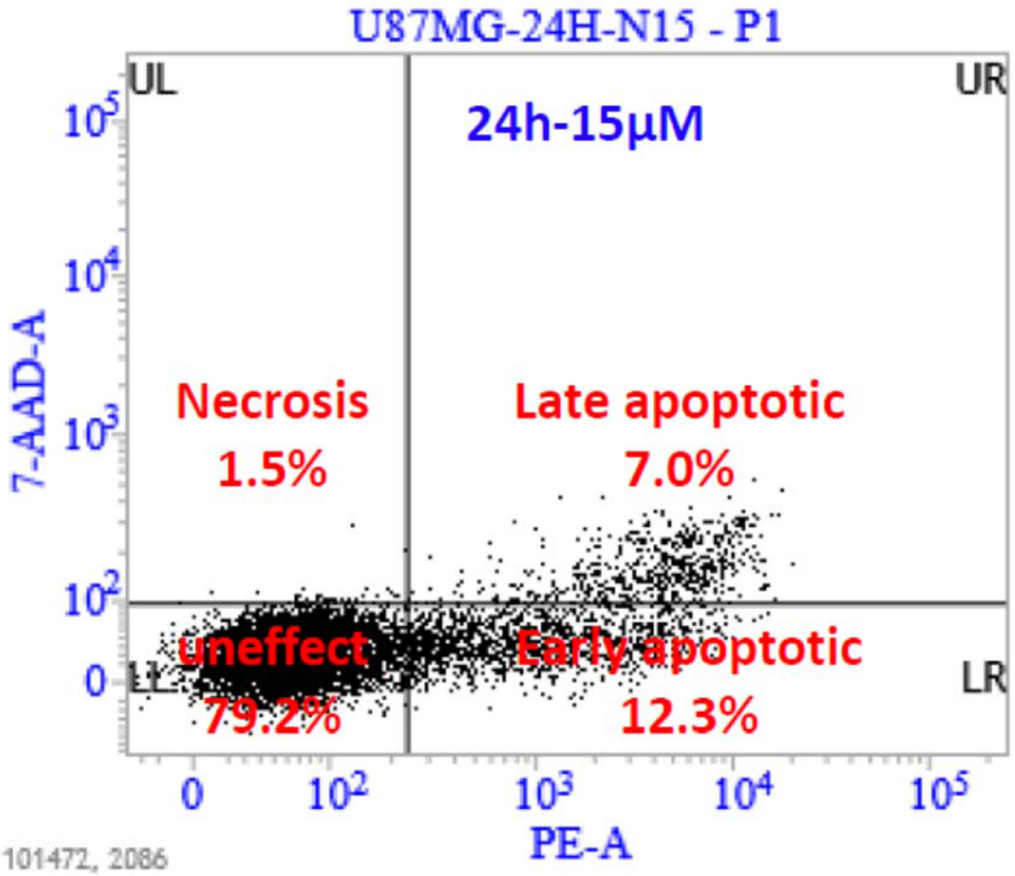
U87MG-24H-N15 - P1
UL
UR
24h-15µM
Necrosis
1.5%
Late apoptotic
7.0%
uneffect
79.2%
Early apoptotic
12.3%
LL
LR
7-AAD-A
PE-A
0
10^2
10^3
10^4
10^5
101472, 2086

U118MG

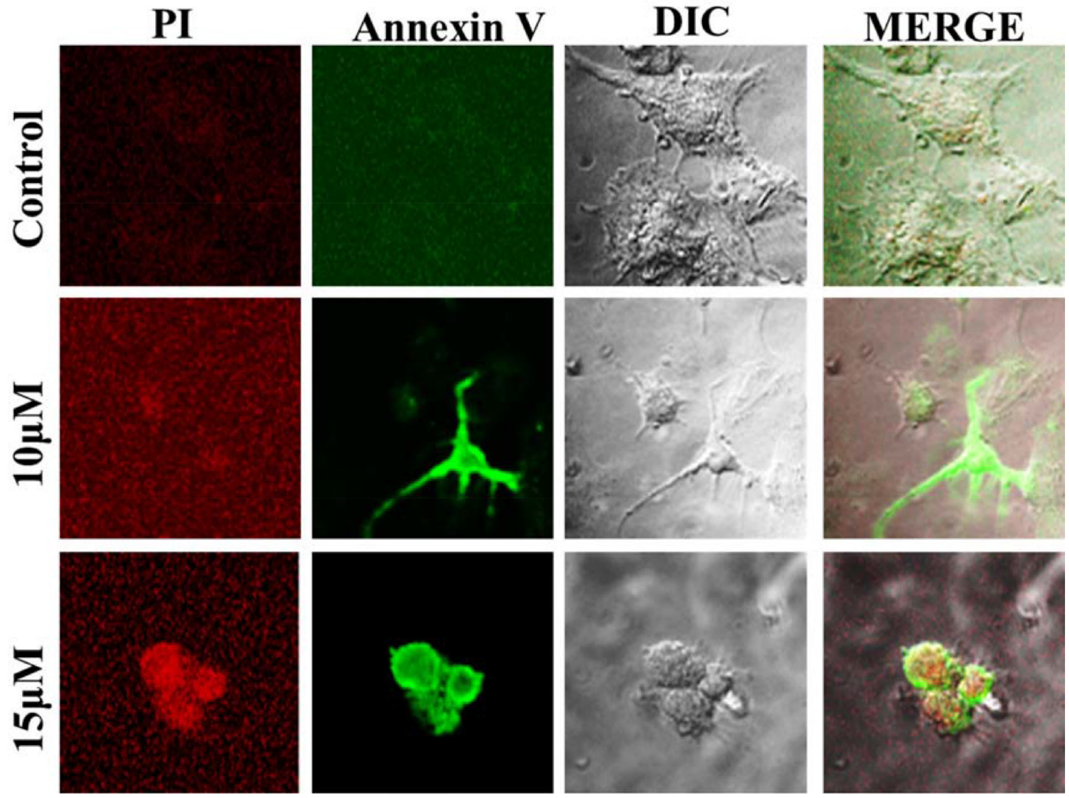
PI
Annexin V
DIC
MERGE
Control
10µM
15µM

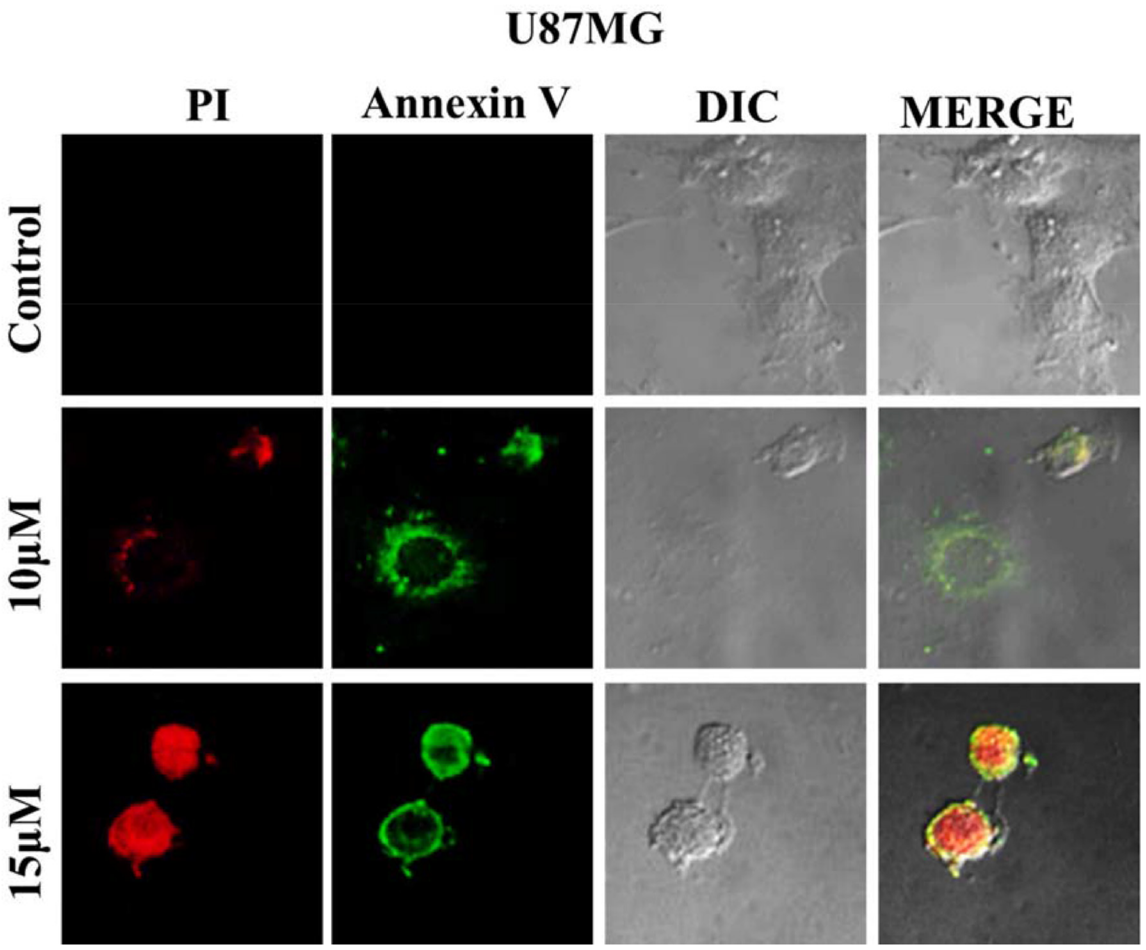
U87MG
PI
Annexin V
DIC
MERGE
Control
10μM
15μM

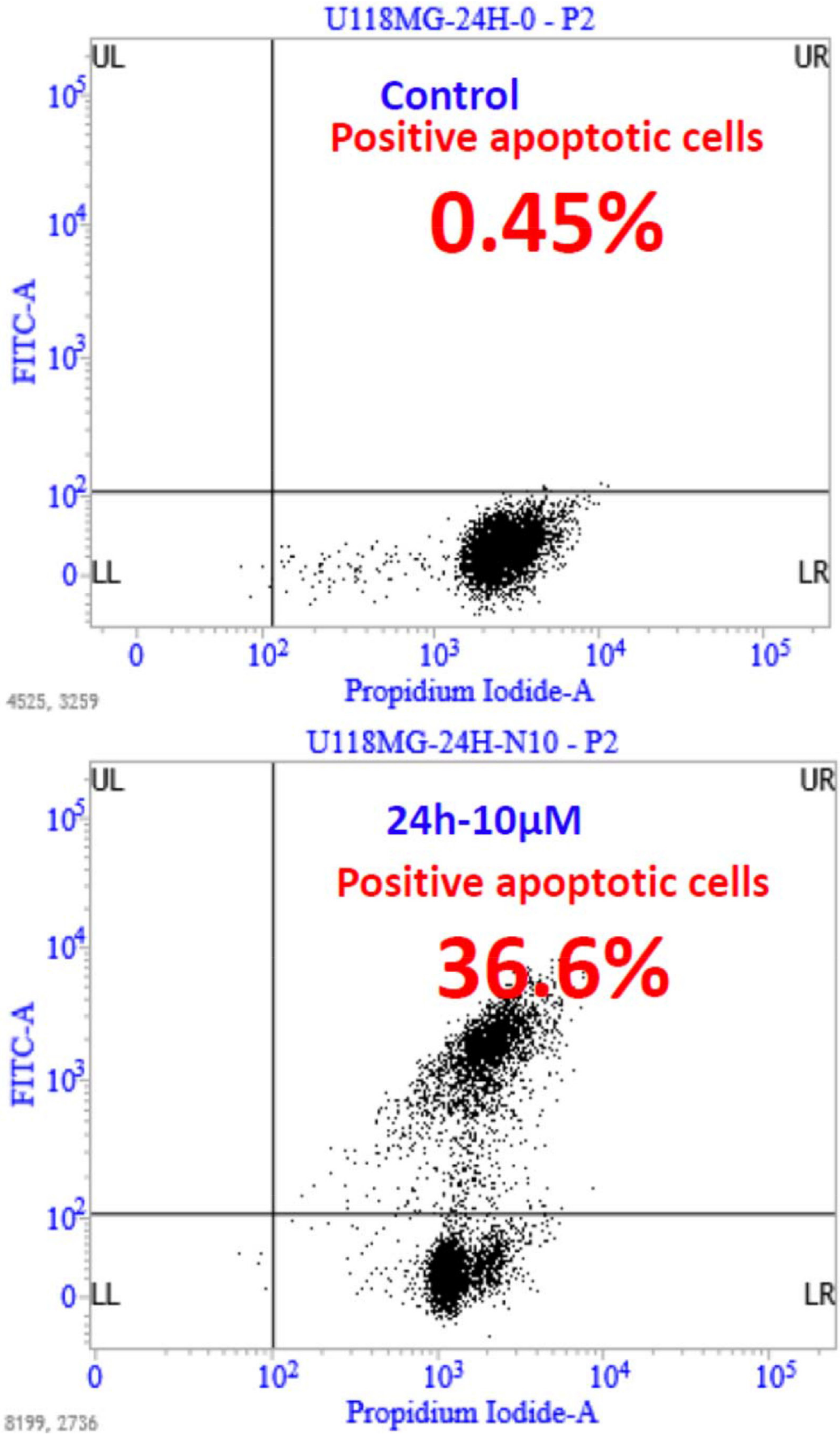
U118MG-24H-0 - P2
UL
UR
Control
Positive apoptotic cells
0.45%
LL
LR
FITC-A
Propidium Iodide-A
4525, 3259
U118MG-24H-N10 - P2
UL
UR
24h-10μM
Positive apoptotic cells
36.6%
LL
LR
FITC-A
Propidium Iodide-A
8199, 2736

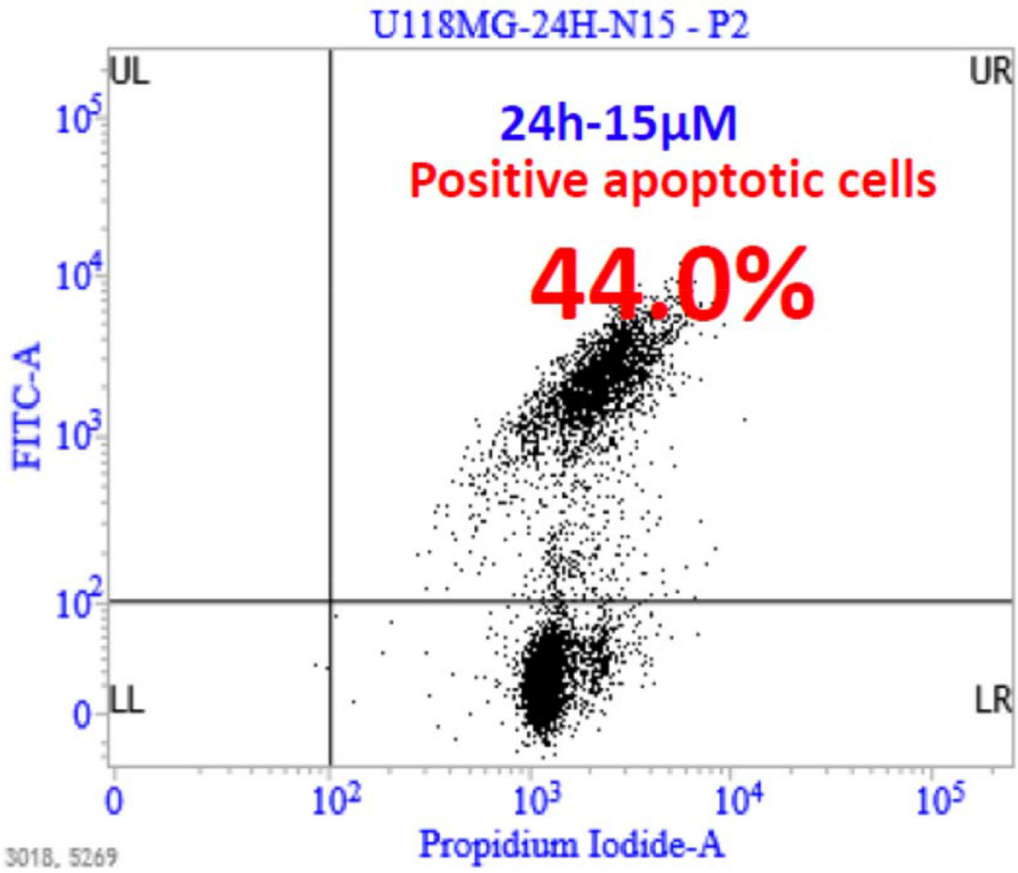
U118MG-24H-N15 - P2
UL
UR
24h-15μM
Positive apoptotic cells
44.0%
LL
LR
FITC-A
Propidium Iodide-A
3018, 5269

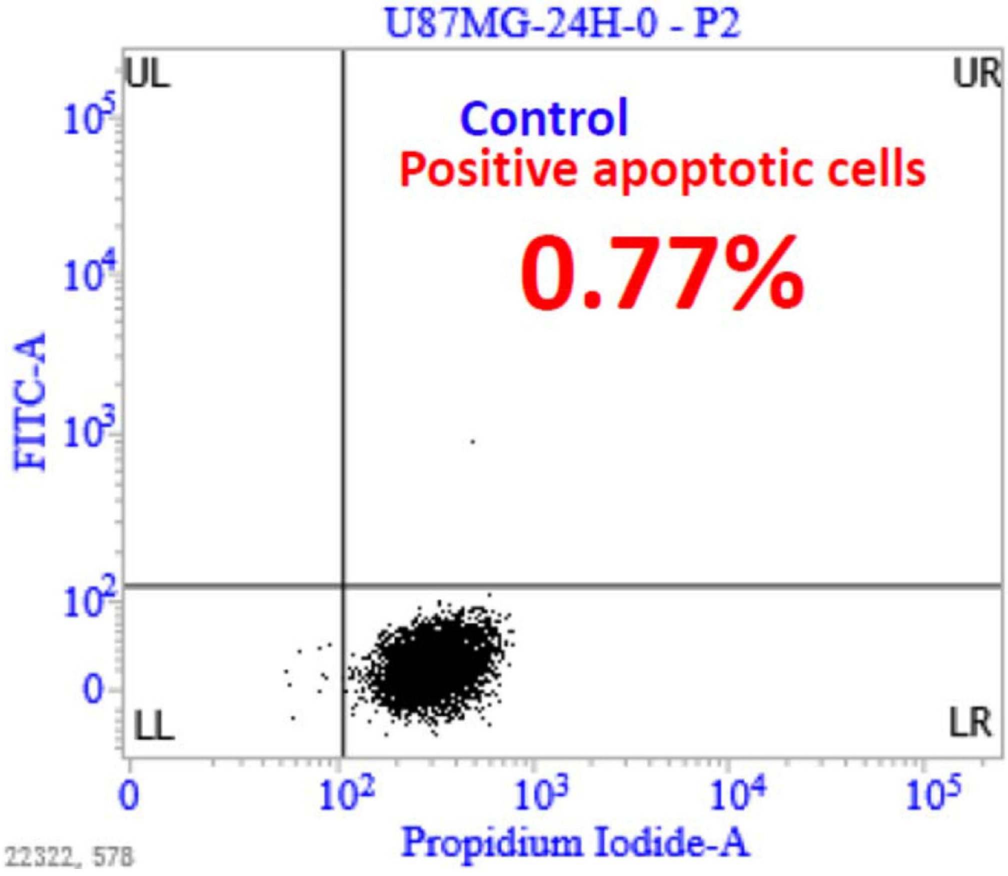
U87MG-24H-0 - P2
UL
UR
Control
Positive apoptotic cells
0.77%
LL
LR
FITC-A
Propidium Iodide-A
22322, 578

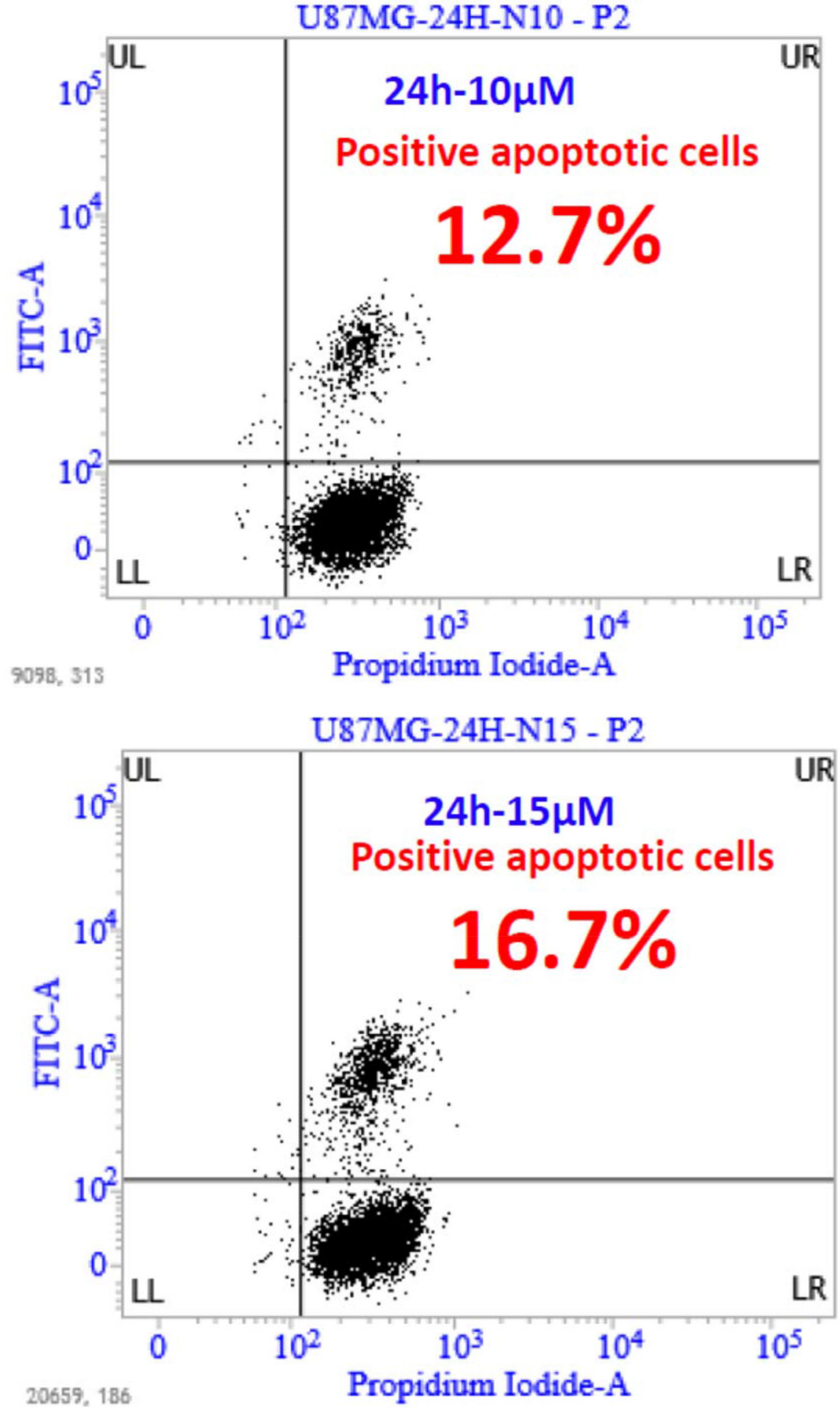

Supplementary Figure 4: Induction of morphological and biochemical features of apoptosis in human glioblastoma cells. Treatments: control (CTL), and incubation with 10 and 15 μM NSC745887 for 24 h. (**A**) Annexin V-PE/7-AAD double staining and flow cytometric analysis of apoptotic populations after treatment. NSC745887 induced a significant population of cells in the A4 area, indicating induction of a biochemical feature of apoptotic death. (**B**) Confocal microscopic imaging to examine morphological features of apoptosis after cells were stained with Annexin V-FITC and propidium iodide. (**C**) Determination of the percentage of apoptosis based on morphological and biochemical features revealed by a TUNEL assay. A significant difference between the control (CTL) and treatment is indicated by $^{*}p < 0.05$ or $^{**}p < 0.01$. Results are representative of three independent experiments.

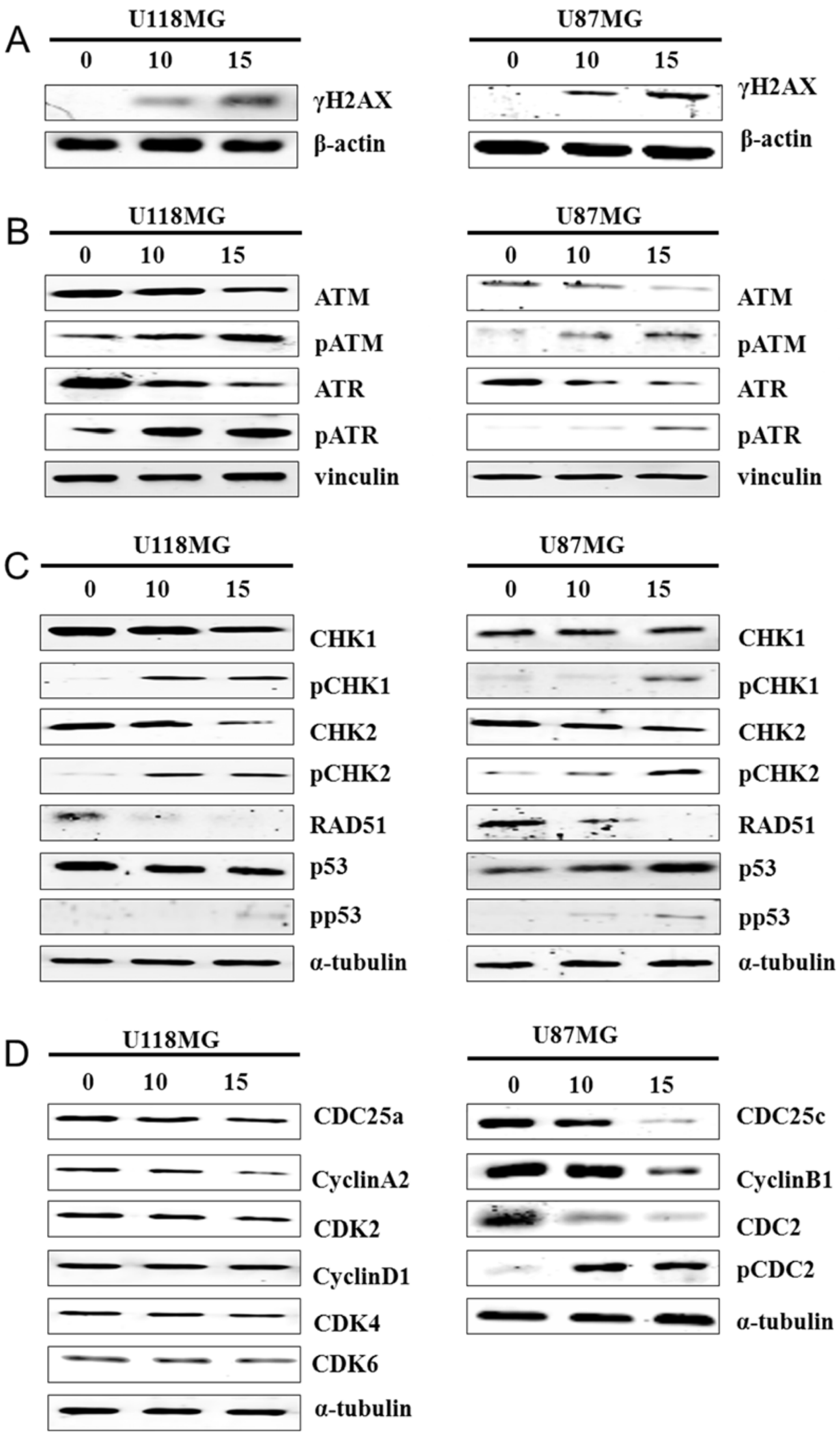

A
U118MG
0 10 15
γH2AX
β-actin
U87MG
0 10 15
γH2AX
β-actin
B
U118MG
0 10 15
ATM
pATM
ATR
pATR
vinculin
U87MG
0 10 15
ATM
pATM
ATR
pATR
vinculin
C
U118MG
0 10 15
CHK1
pCHK1
CHK2
pCHK2
RAD51
p53
pp53
α-tubulin
U87MG
0 10 15
CHK1
pCHK1
CHK2
pCHK2
RAD51
p53
pp53
α-tubulin
D
U118MG
0 10 15
CDC25a
CyclinA2
CDK2
CyclinD1
CDK4
CDK6
α-tubulin
U87MG
0 10 15
CDC25c
CyclinB1
CDC2
pCDC2
α-tubulin

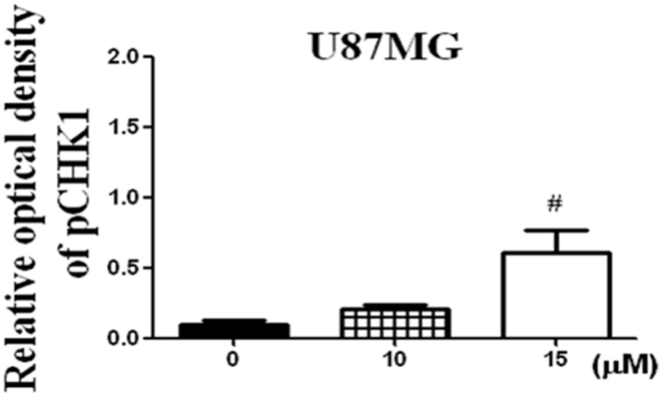
U87MG
Relative optical density of pCHK1
2.0
1.5
1.0
0.5
0.0
#
0
10
15
(μM)

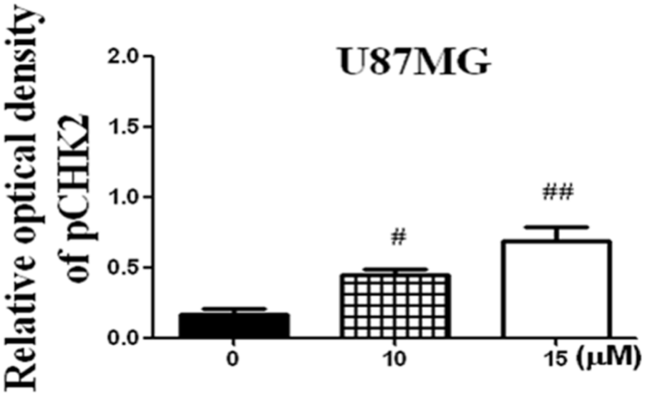
U87MG
Relative optical density of pCHK2
2.0
1.5
1.0
0.5
0.0
#
##
0
10
15
(μM)

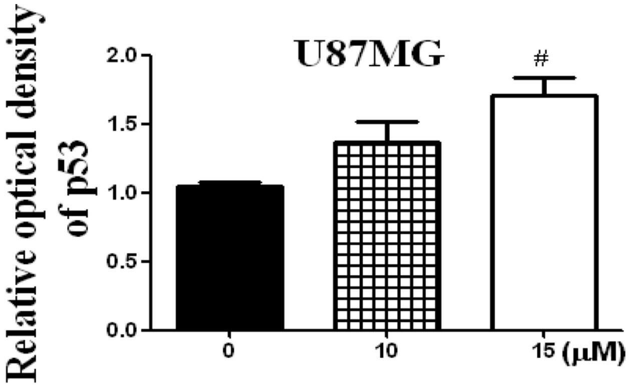
U87MG
Relative optical density of p53
2.0
1.5
1.0
0.5
0.0
#
0
10
15
(μM)

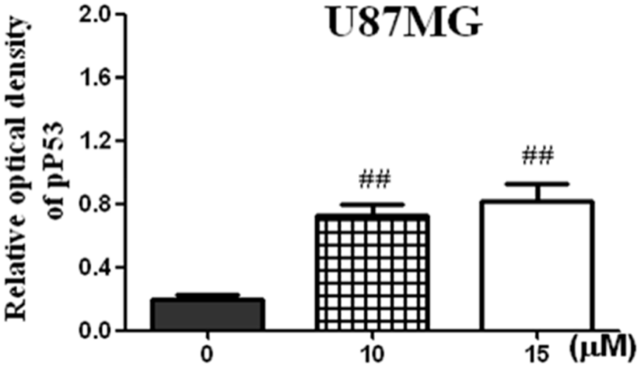
U87MG
Relative optical density of pP53
2.0
1.6
1.2
0.8
0.4
0.0
##
##
0
10
15
(μM)

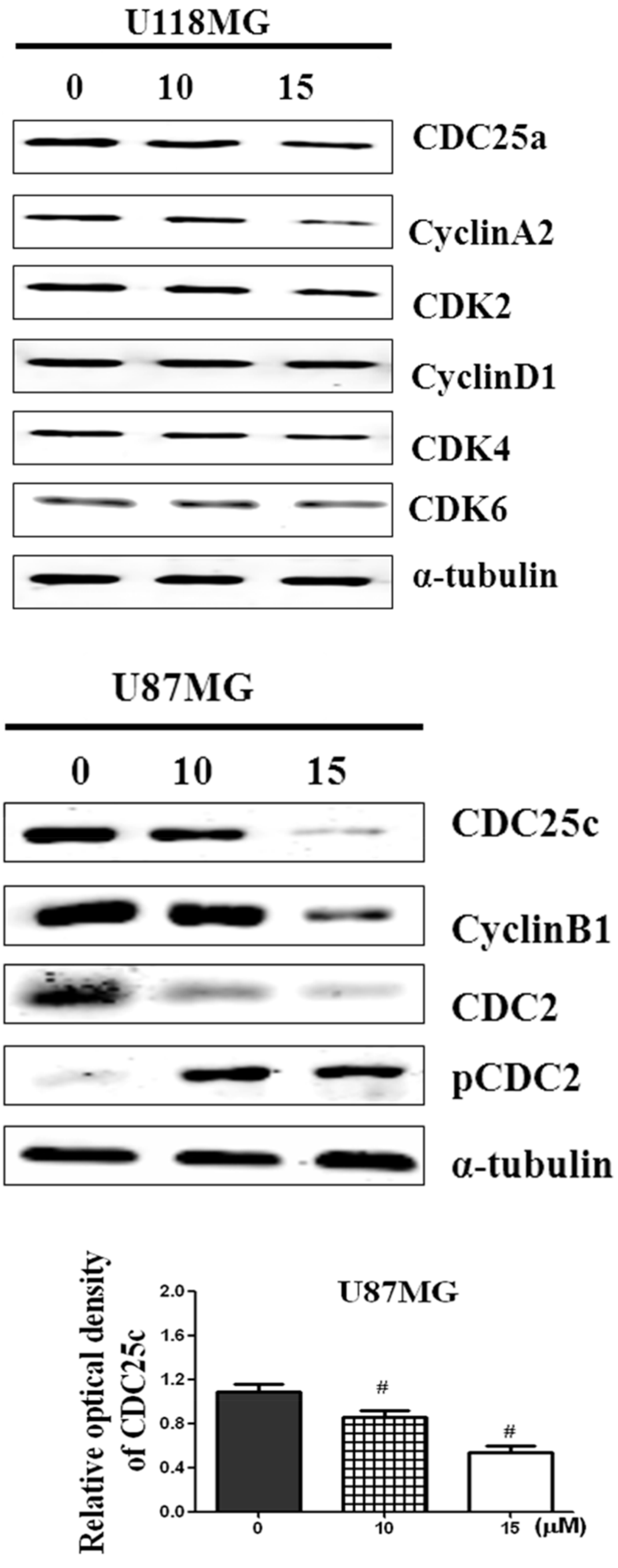

U118MG
0 10 15
CDC25a
CyclinA2
CDK2
CyclinD1
CDK4
CDK6
α-tubulin
U87MG
0 10 15
CDC25c
CyclinB1
CDC2
pCDC2
α-tubulin
U87MG
Relative optical density of CDC25c
2.0
1.6
1.2
0.8
0.4
0.0
#
#
0 10 15 (μM)

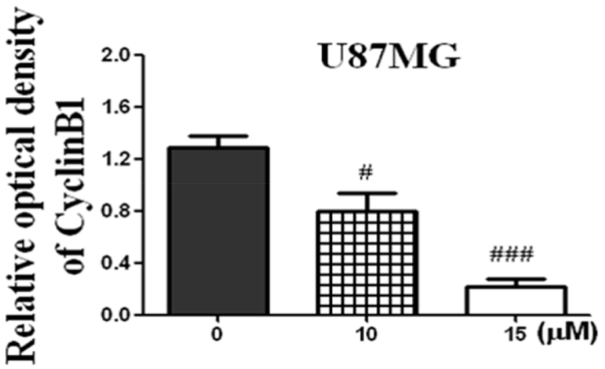
U87MG
Relative optical density of CyclinB1
2.0
1.6
1.2
0.8
0.4
0.0
#
###
0
10
15
(μM)

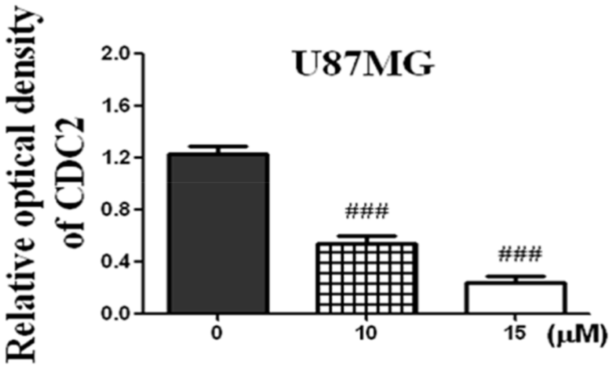
U87MG
Relative optical density of CDC2
2.0
1.6
1.2
0.8
0.4
0.0
###
###
0
10
15
(μM)

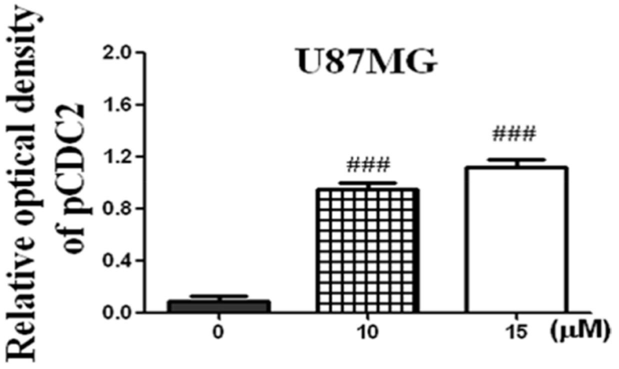
U87MG
Relative optical density of pCDC2
2.0
1.6
1.2
0.8
0.4
0.0
###
###
0
10
15
(μM)

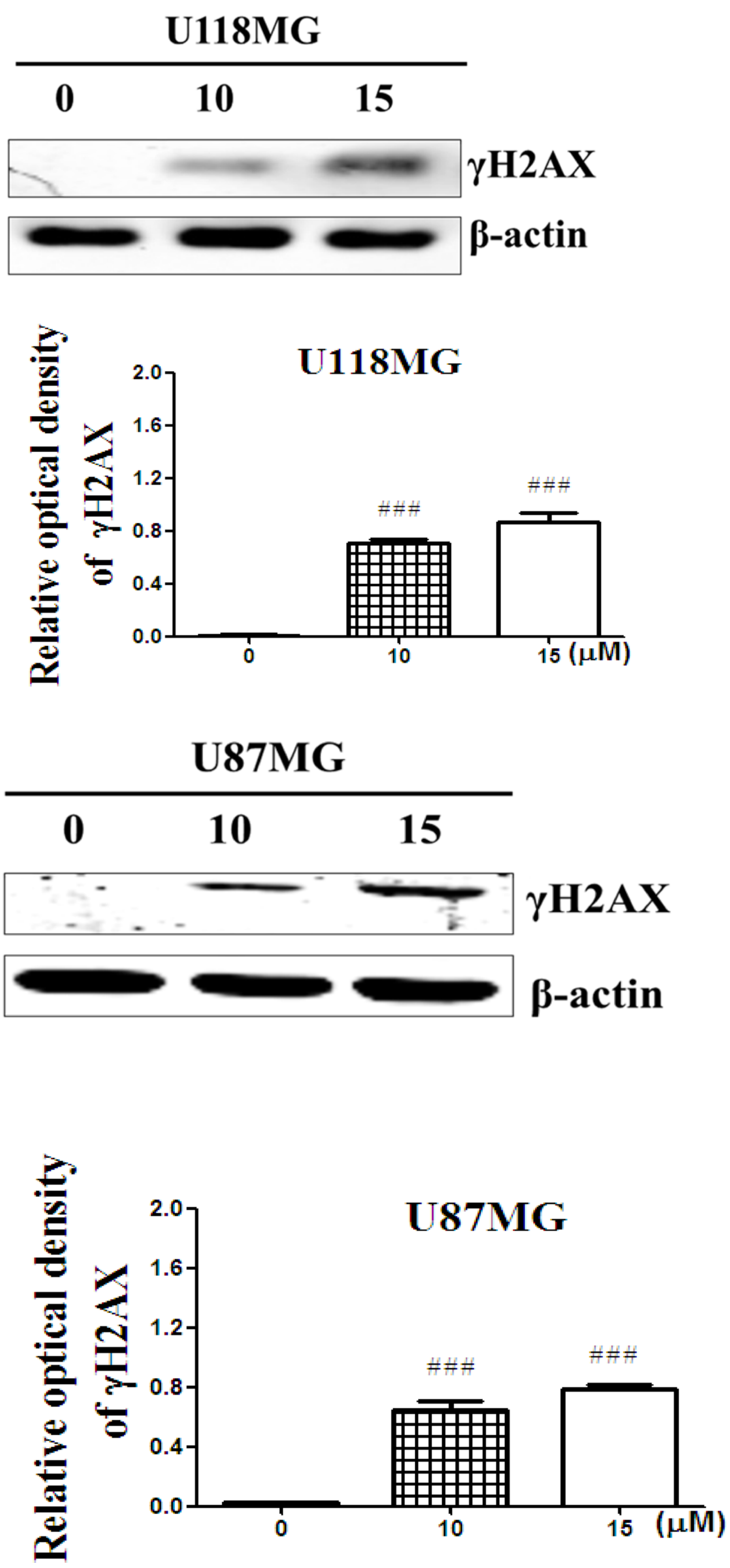
U118MG
0
10
15
γH2AX
β-actin
Relative optical density of γH2AX
U118MG
2.0
1.6
1.2
0.8
0.4
0.0
###
###
0
10
15 (μM)
U87MG
0
10
15
γH2AX
β-actin
Relative optical density of γH2AX
U87MG
2.0
1.6
1.2
0.8
0.4
0.0
###
###
0
10
15 (μM)

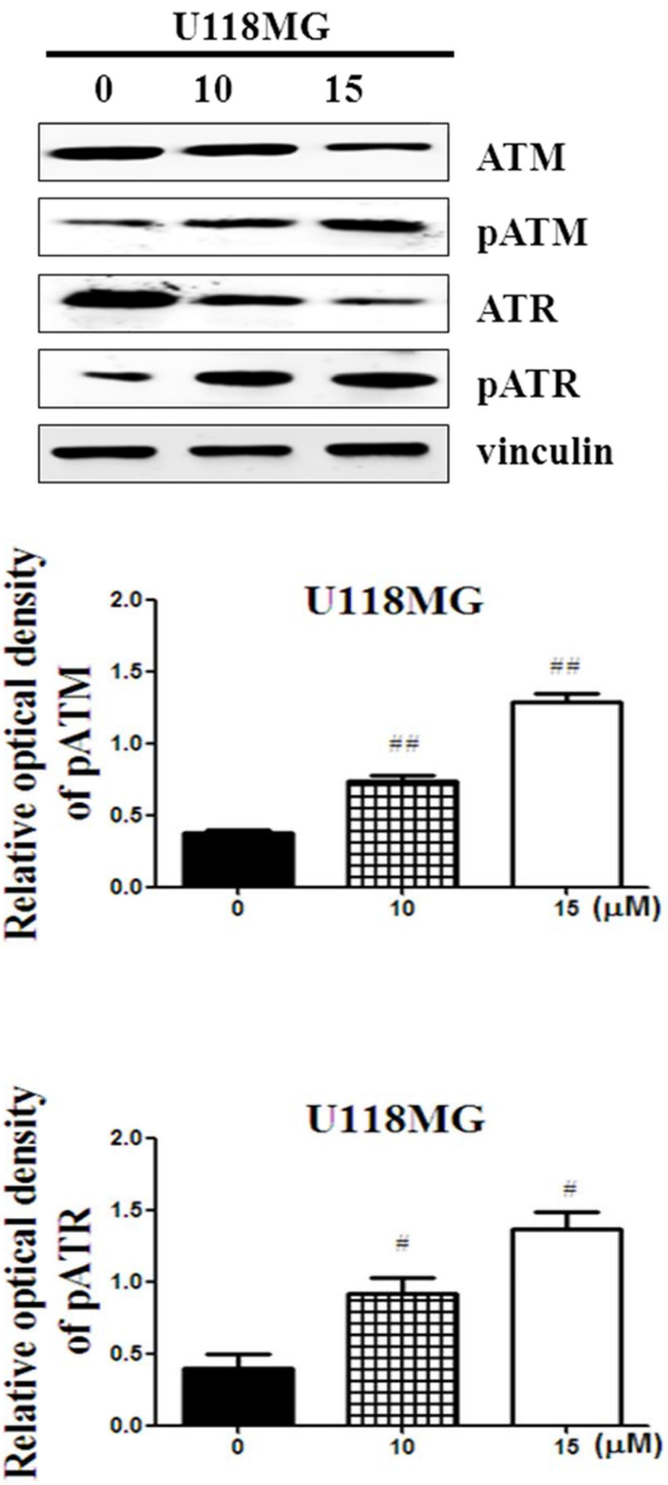
U118MG
0
10
15
ATM
pATM
ATR
pATR
vinculin
U118MG
Relative optical density of pATM
2.0
1.5
1.0
0.5
0.0
##
##
0
10
15 (μM)
U118MG
Relative optical density of pATR
2.0
1.5
1.0
0.5
0.0
#
#
0
10
15 (μM)

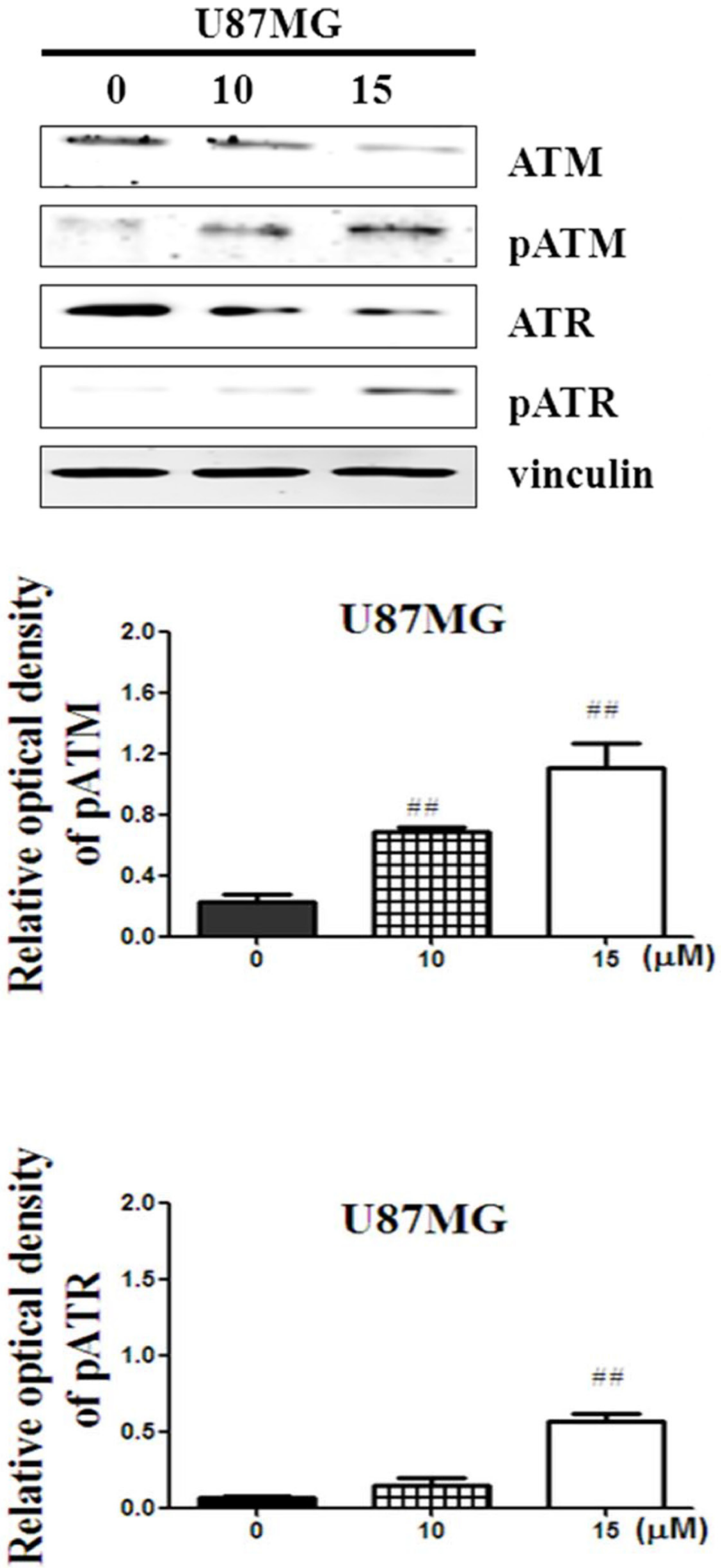

U87MG
0
10
15
ATM
pATM
ATR
pATR
vinculin
U87MG
Relative optical density of pATM
2.0
1.6
1.2
0.8
0.4
0.0
##
##
0
10
15
(μM)
U87MG
Relative optical density of pATR
2.0
1.5
1.0
0.5
0.0
##
0
10
15
(μM)

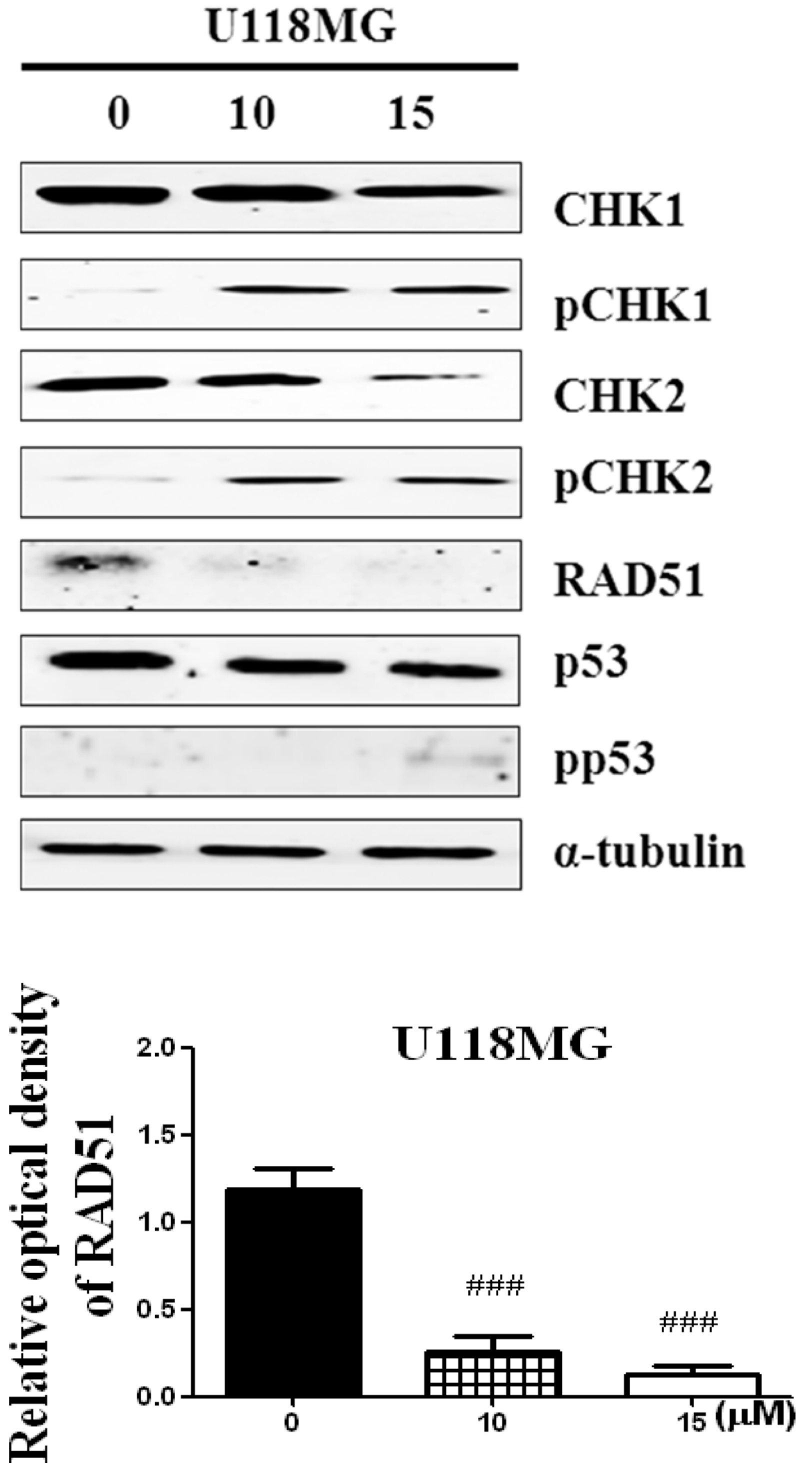
U118MG
0
10
15
CHK1
pCHK1
CHK2
pCHK2
RAD51
p53
pp53
α-tubulin
U118MG
Relative optical density of RAD51
2.0
1.5
1.0
0.5
0.0
###
###
0
10
15 (μM)

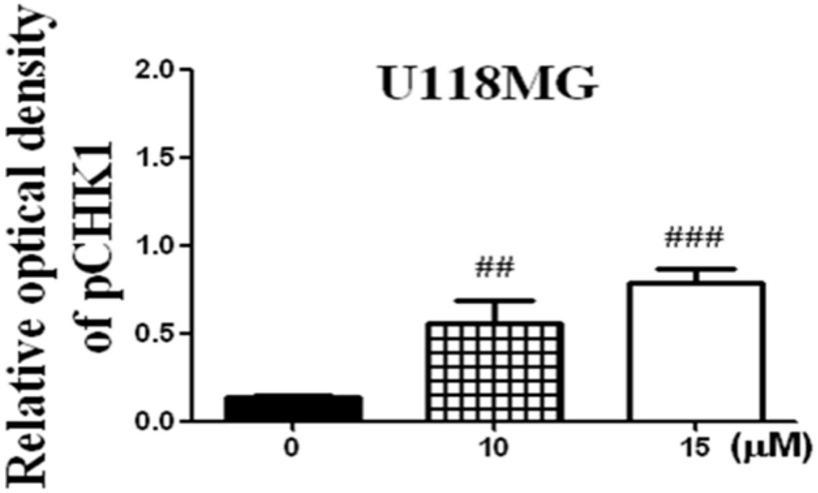

U118MG
Relative optical density
of pCHK1
2.0
1.5
1.0
0.5
0.0
##
###
0
10
15 (μM)

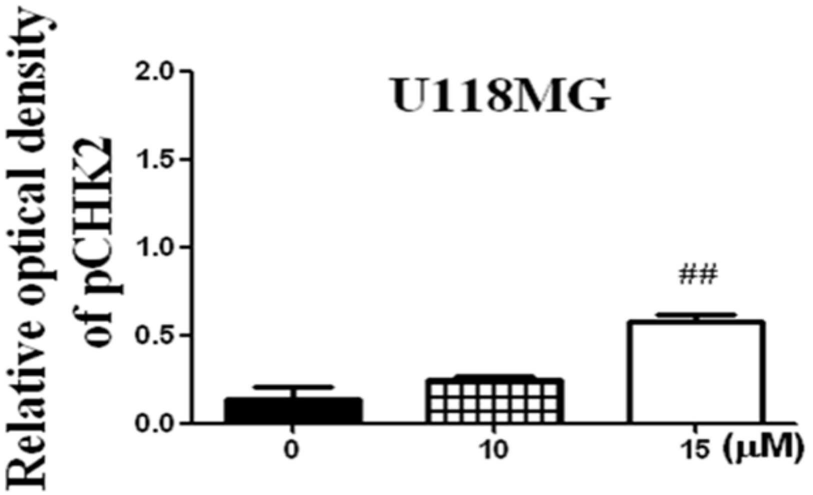

U118MG
Relative optical density
of pCHK2
2.0
1.5
1.0
0.5
0.0
##
0
10
15 (μM)

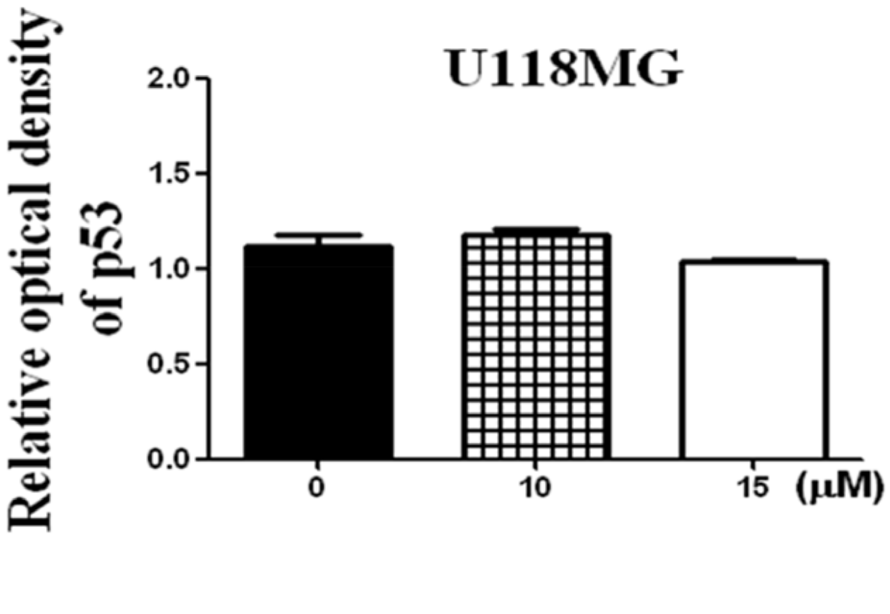
U118MG
Relative optical density of p53
2.0
1.5
1.0
0.5
0.0
0
10
15 (μM)

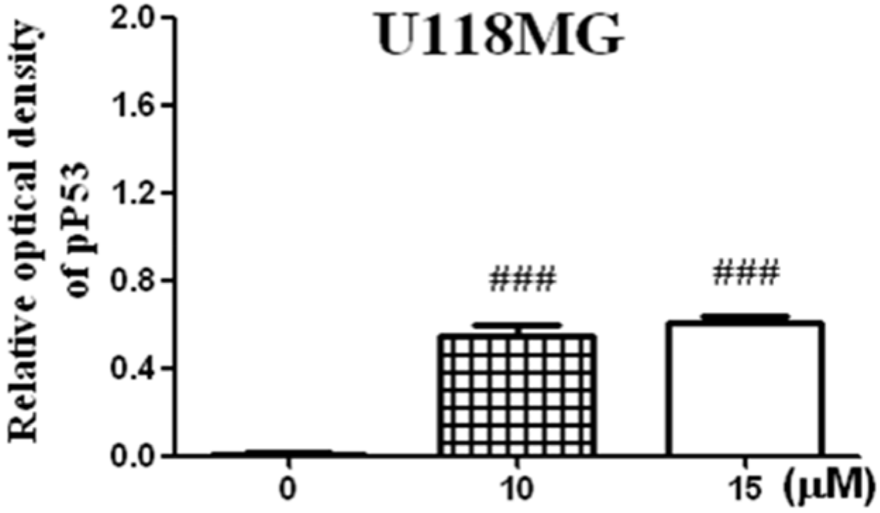
U118MG
Relative optical density of pP53
2.0
1.6
1.2
0.8
0.4
0.0
###
###
0
10
15 (μM)

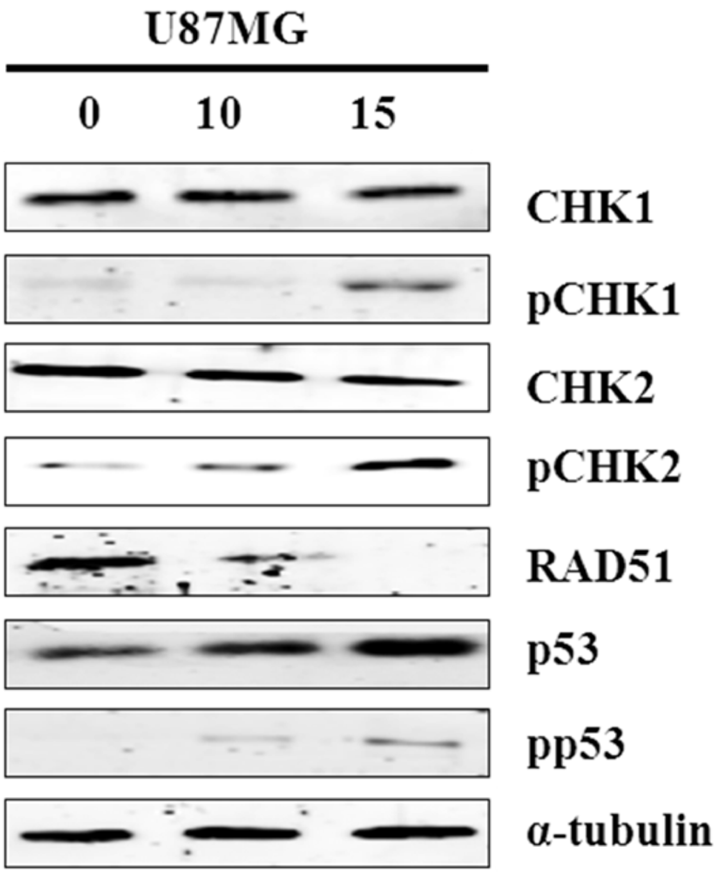

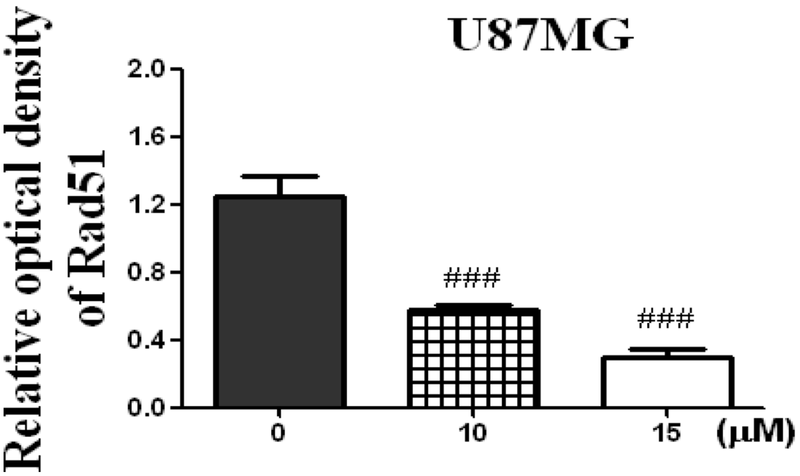

Supplementary Figure 5: NSC745887 activates ATM and ATR signaling. Protein expression levels of (**A**) γH2AX; (**B**) ATM, phosphorylated (p)-ATM, ATR, p-ATR, CHK1, p-CHK1, CHK2, p-CHK2, RAD51, p53, p-p53 (**C**) CHK1, pCHK1, CHK2, pCHK2, RAD51, p53, pp53, and α-tubulin were detected in cells treated with or without NSC745887 (10 or 15 μM) for 24 h using Western blotting. β-Actin, vinculin and α-tubulin were used as loading controls. (**D**) CDC25a, cyclin A2, CDK2, cyclin D1, CDK4/6, CDC25c, cyclin B1, CDC2 and p-CDC2 were detected in cells treated with or without NSC745887 (10 or 15 μM) for 24 h using Western blotting. β-Actin, vinculin and α-tubulin were used as loading controls. . Data are presented as the mean ± SD; statistical significance is indicated by $^{\#}p < 0.05$, $^{\#\#}p < 0.01$, $^{\#\#\#}p < 0.001$ compared to the control.

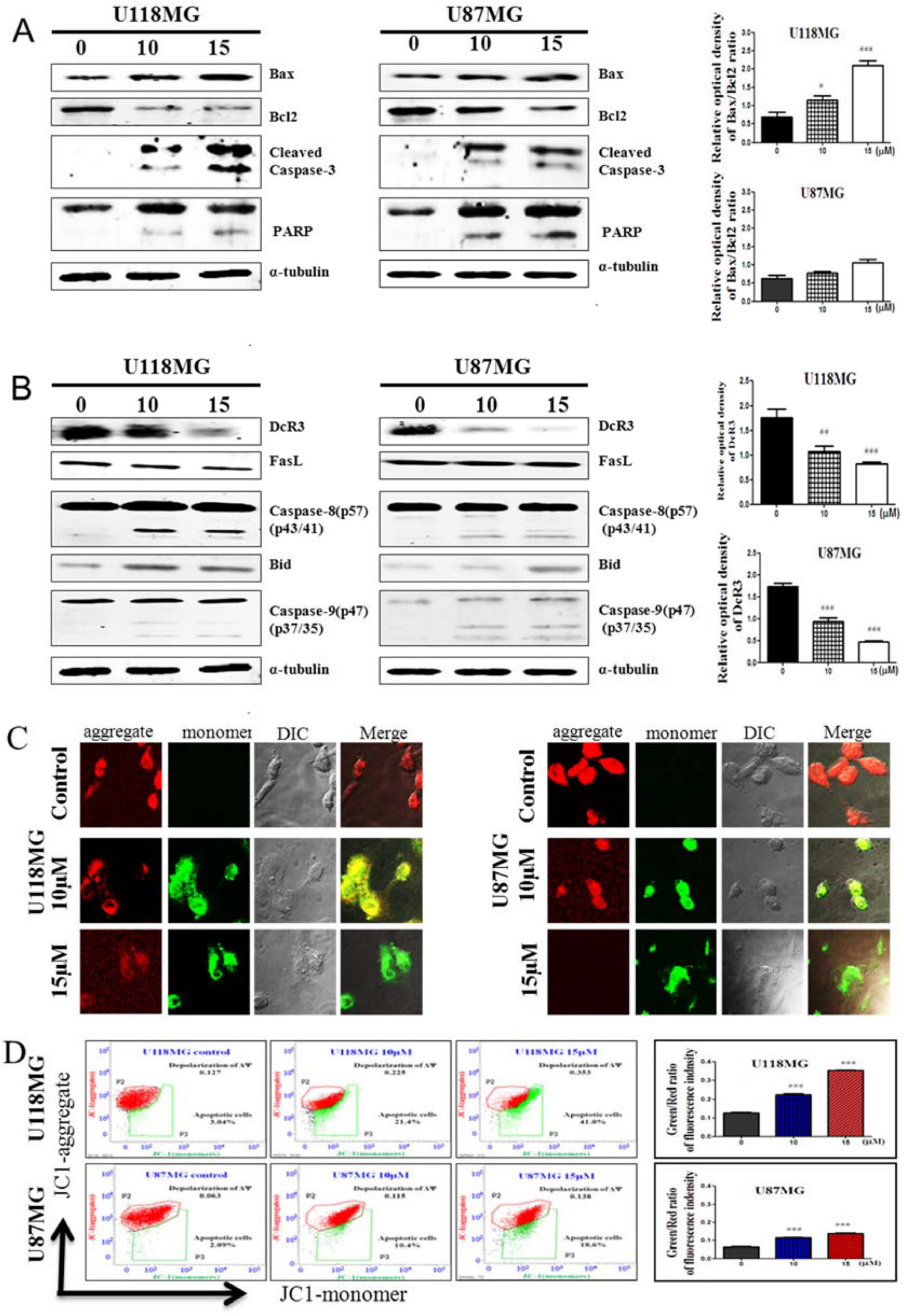
A
U118MG
U87MG
0 10 15
Bax
Bcl2
Cleaved Caspase-3
PARP
α-tubulin
Relative optical density of Bax/Bcl2 ratio
15 (μM)
B
DcR3
FasL
Caspase-8(p57) (p43/41)
Bid
Caspase-9(p47) (p37/35)
Relative optical density of DcR3
C
aggregate
monomer
DIC
Merge
Control
10μM
15μM
D
U118MG control
U118MG 10μM
U118MG 15μM
U87MG control
U87MG 10μM
U87MG 15μM
JC1-aggregate
JC1-monomer
Green/Red ratio of fluorescence indensity

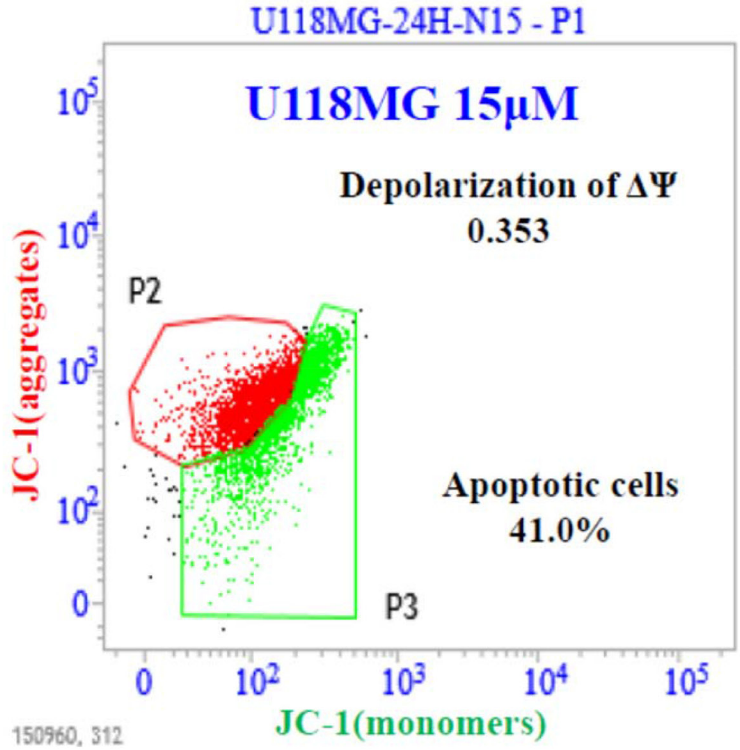
U118MG-24H-N15 - P1
U118MG 15μM
Depolarization of ΔΨ
0.353
Apoptotic cells
41.0%
P2
P3
JC-1(aggregates)
JC-1(monomers)
150960, 312

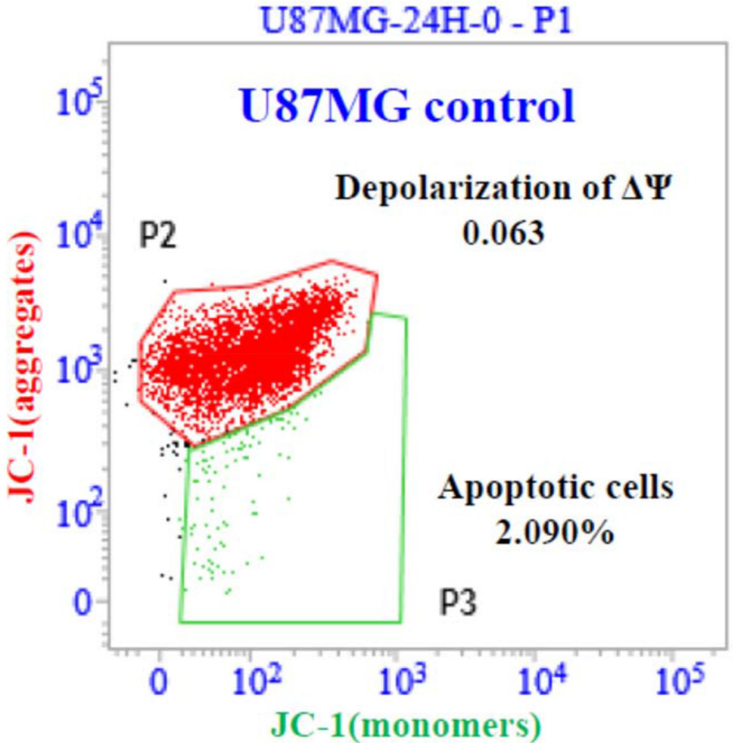
U87MG-24H-0 - P1
U87MG control
Depolarization of ΔΨ
0.063
Apoptotic cells
2.090%
P2
P3
JC-1(aggregates)
JC-1(monomers)

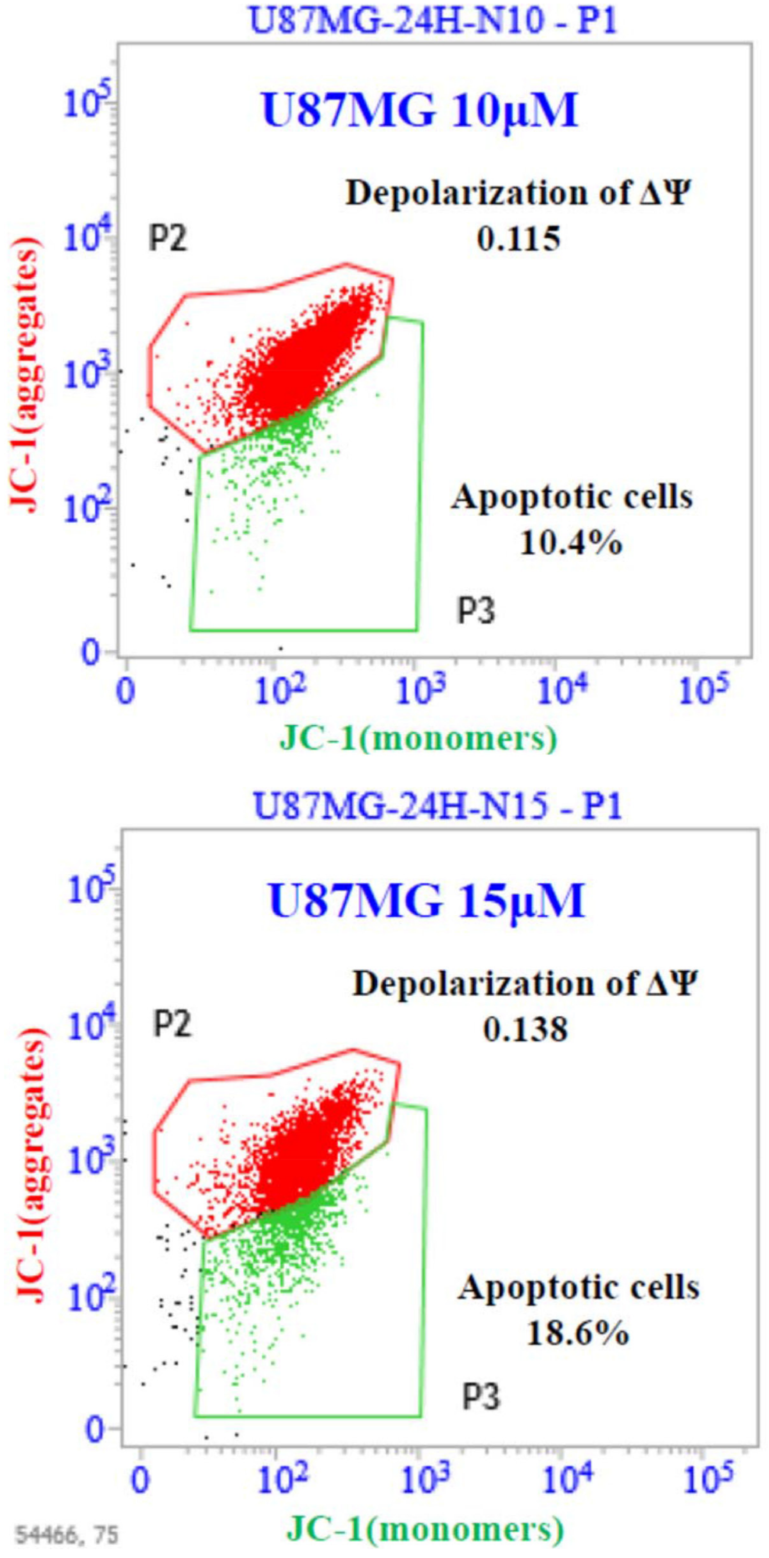
U87MG-24H-N10 - P1
U87MG 10μM
Depolarization of ΔΨ
0.115
P2
Apoptotic cells
10.4%
P3
JC-1(aggregates)
JC-1(monomers)
U87MG-24H-N15 - P1
U87MG 15μM
Depolarization of ΔΨ
0.138
P2
Apoptotic cells
18.6%
P3
JC-1(aggregates)
JC-1(monomers)
54466, 75

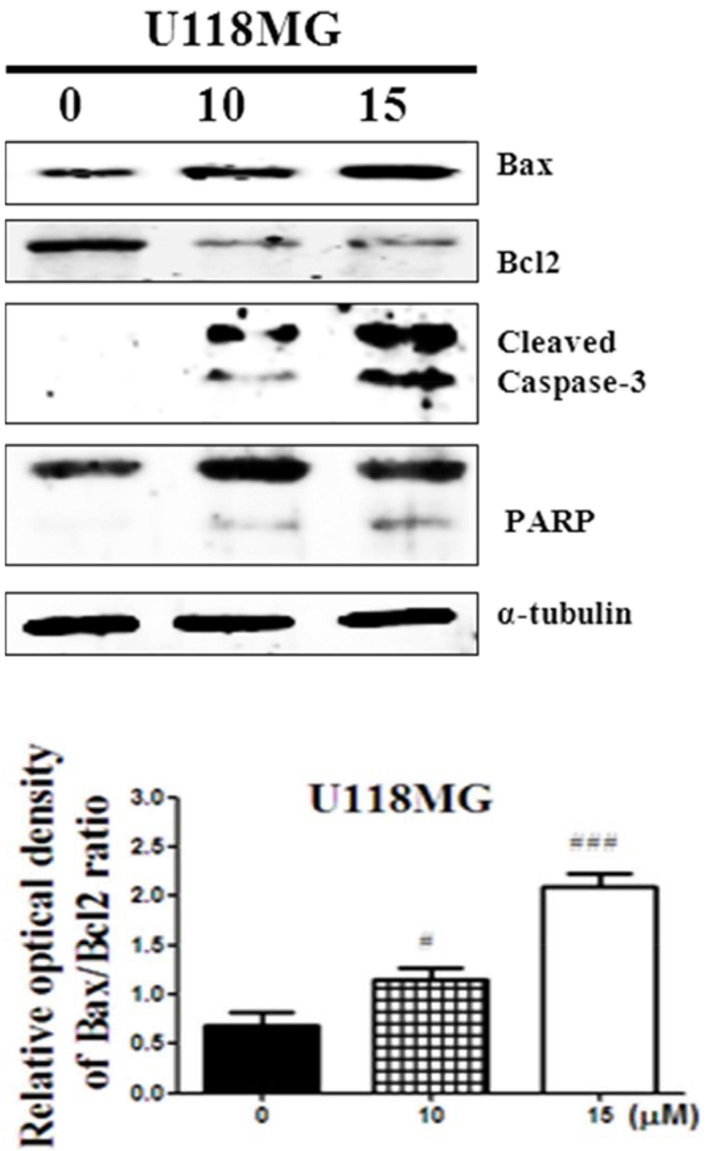
U118MG
0
10
15
Bax
Bcl2
Cleaved
Caspase-3
PARP
α-tubulin
U118MG
Relative optical density
of Bax/Bcl2 ratio
3.0
2.5
2.0
1.5
1.0
0.5
0.0
###
#
0
10
15
(μM)

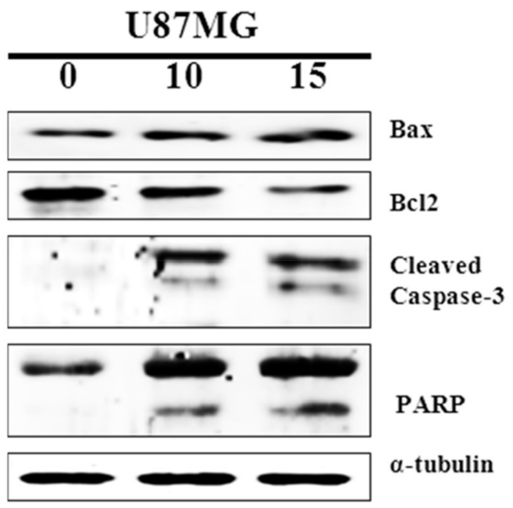

U87MG
0
10
15
Bax
Bcl2
Cleaved
Caspase-3
PARP
α-tubulin

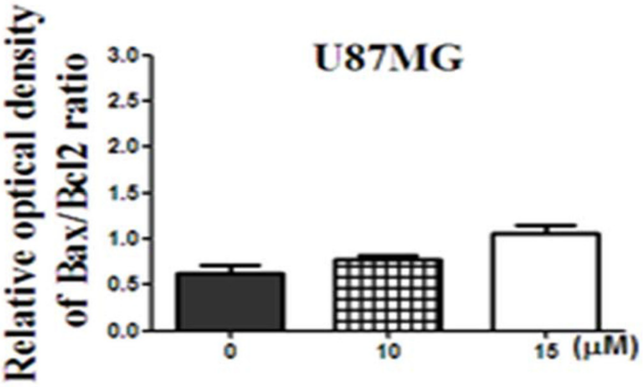

U87MG
Relative optical density
of Bax/Bcl2 ratio
3.0
2.5
2.0
1.5
1.0
0.5
0.0
0
10
15
(μM)

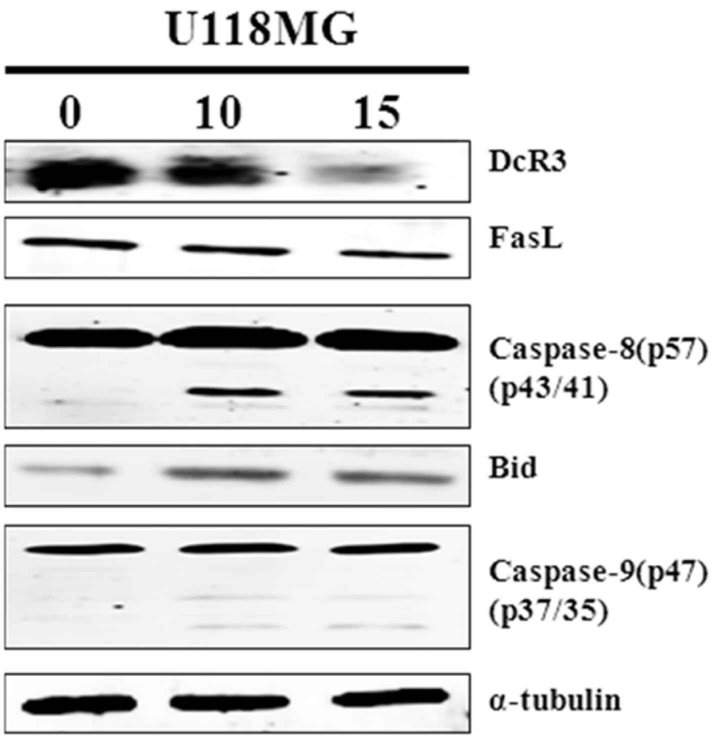

U118MG
0
10
15
DcR3
FasL
Caspase-8(p57)
(p43/41)
Bid
Caspase-9(p47)
(p37/35)
α-tubulin

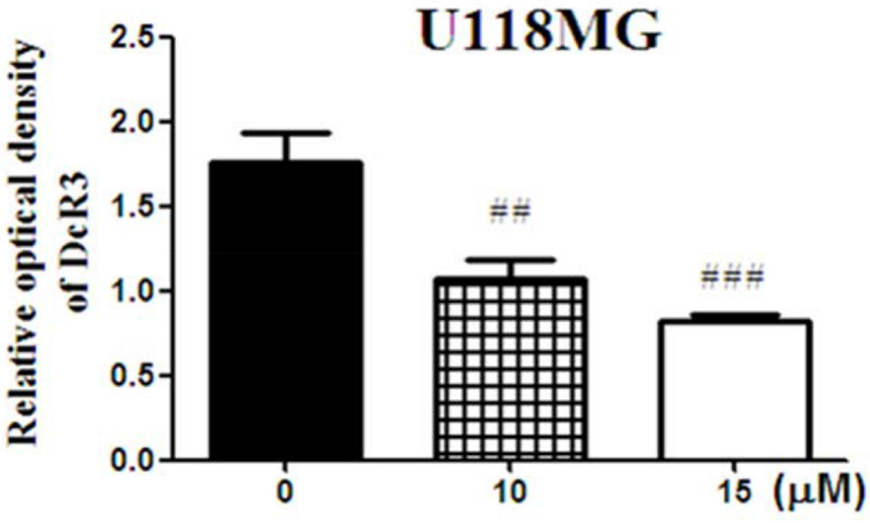
U118MG
Relative optical density of DcR3
2.5
2.0
1.5
1.0
0.5
0.0
##
###
0
10
15 (μM)

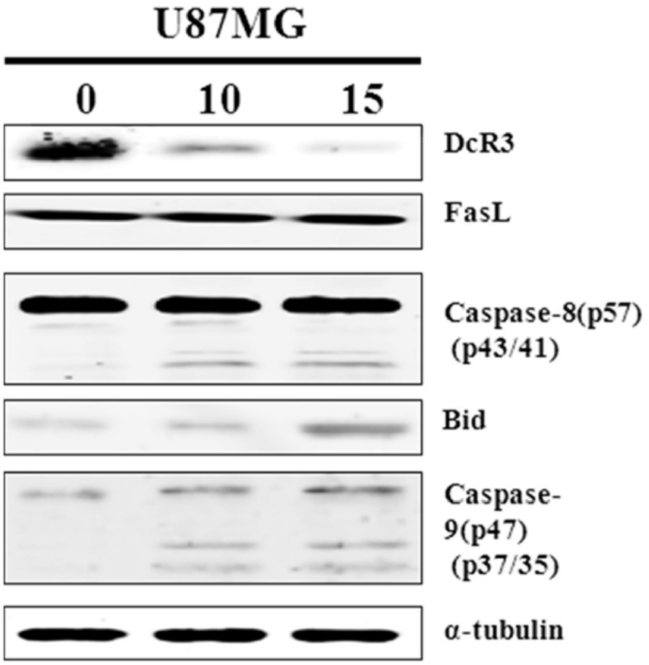
U87MG
0
10
15
DcR3
FasL
Caspase-8(p57)
(p43/41)
Bid
Caspase-9(p47)
(p37/35)
α-tubulin

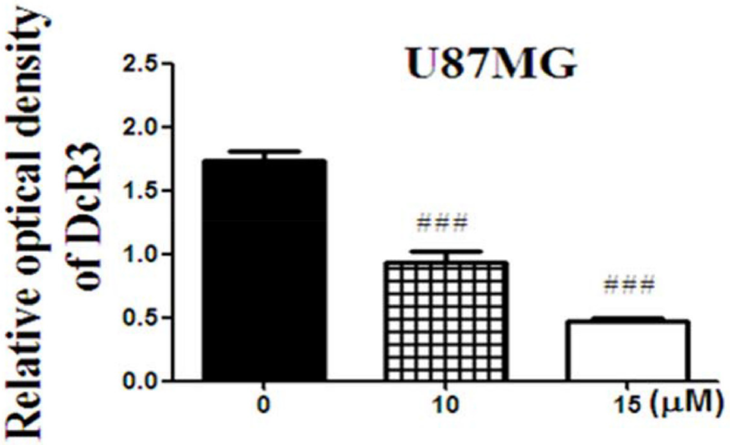
U87MG
Relative optical density of DcR3
2.5
2.0
1.5
1.0
0.5
0.0
###
###
0
10
15 (μM)

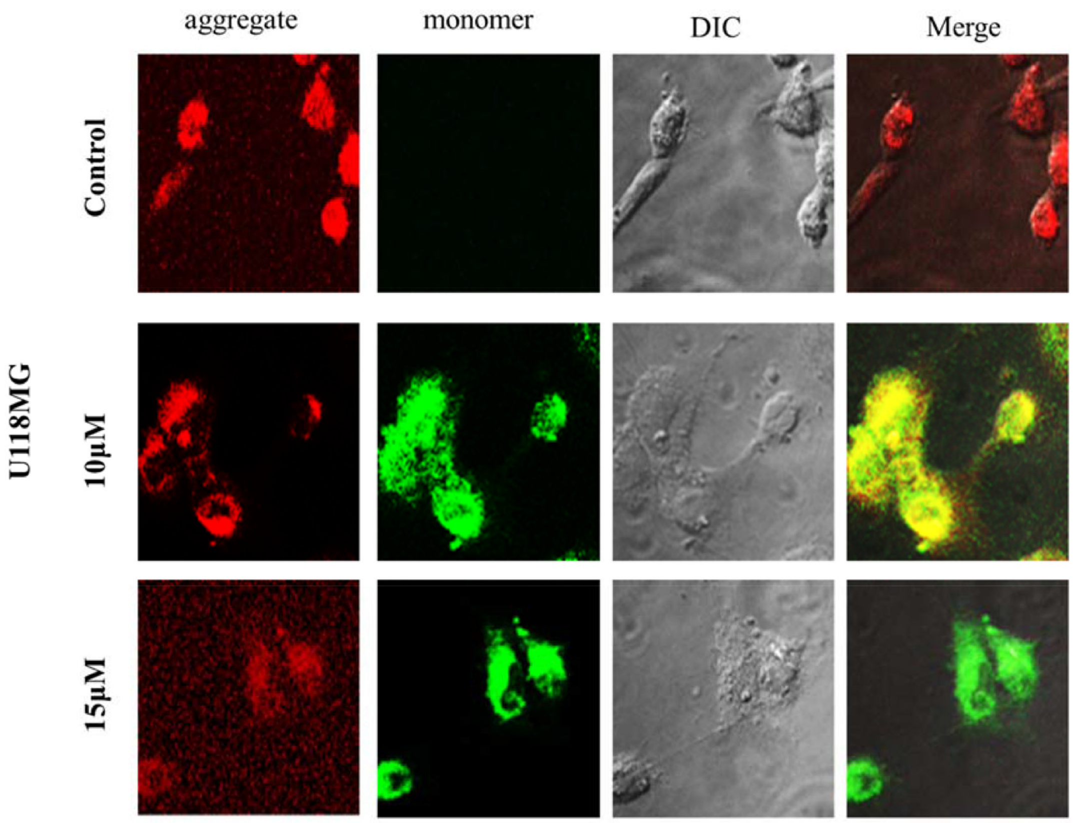
aggregate
monomer
DIC
Merge
U118MG
Control
10μM
15μM

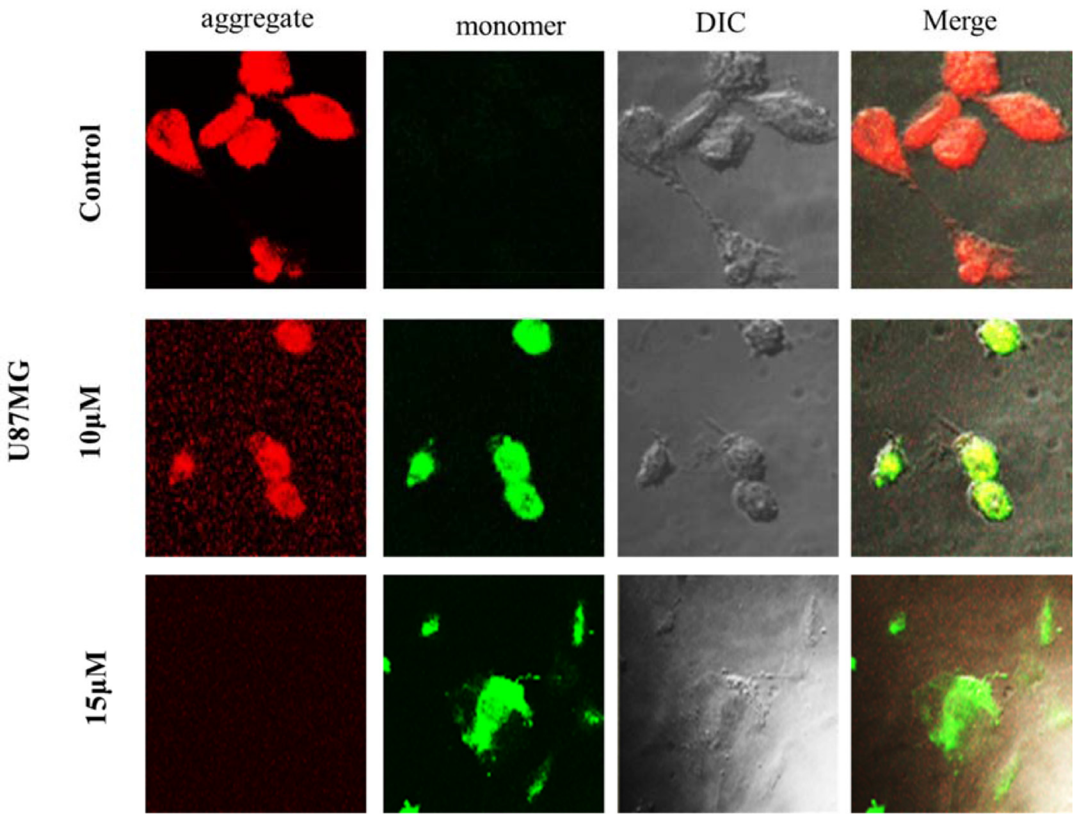
aggregate
monomer
DIC
Merge
U87MG
Control
10μM
15μM

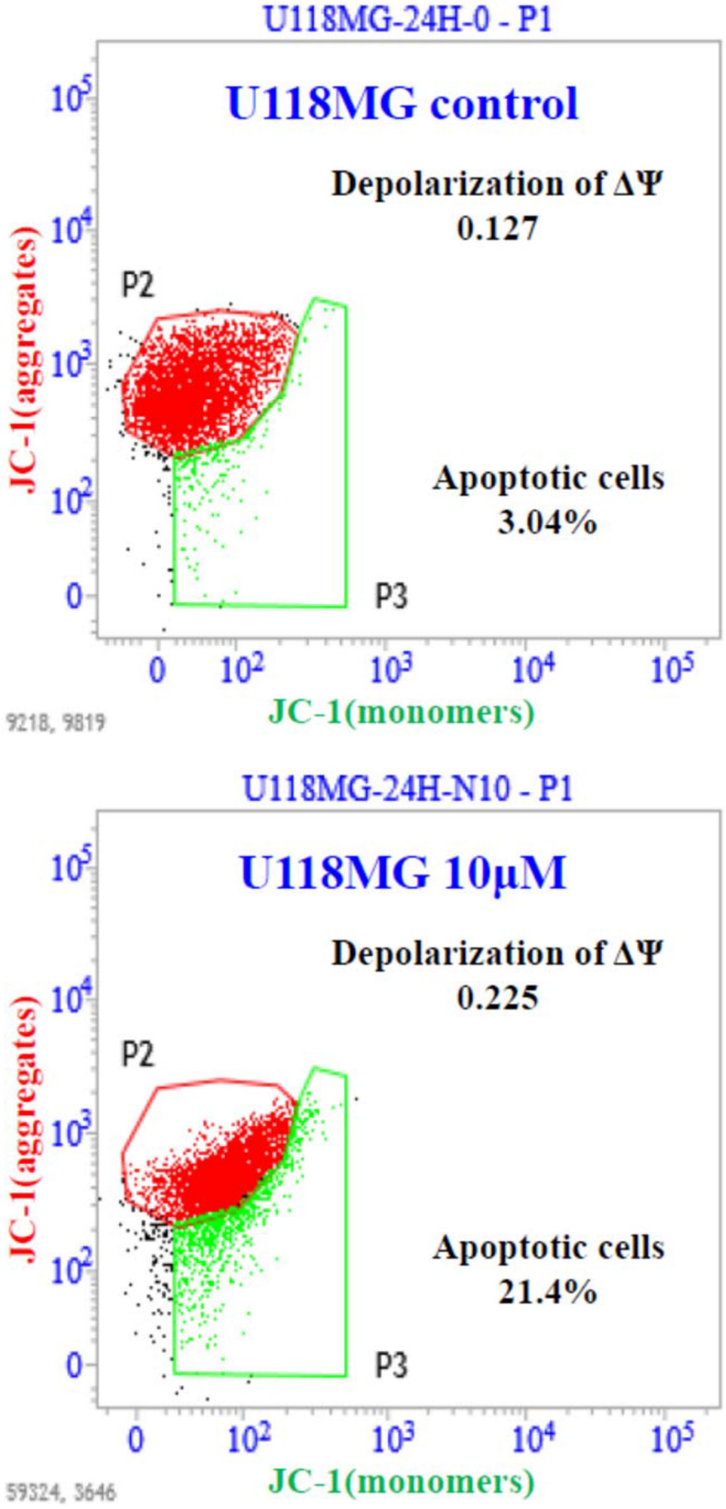

Supplementary Figure 6: NSC-745887 engages the intrinsic and extrinsic apoptotic pathways. Protein expression levels of (**A**) Bax, Bcl2, cleaved caspase-3 and poly (ADP ribose) polymerase (PARP); (**B**) DcR3, FasL, cleaved caspase-8, Bid, and cleaved caspase-9 were detected in cells treated with or without NSC745887 (10 or 15 μM) for 24 h by Western blotting. α-Tubulin was used as a loading control. (**C**) Fluorescence staining of JC-1, which shows the mitochondrial membrane potential change, was analyzed by confocal microscopy to show the cell morphology and then (**D**) was detected by a flow cytometric analysis. Data are presented as the mean ± SD; statistical significance is indicated by $^{\#}p < 0.05$, $^{\#\#}p < 0.01$, $^{\#\#\#}p < 0.001$ compared to the control.

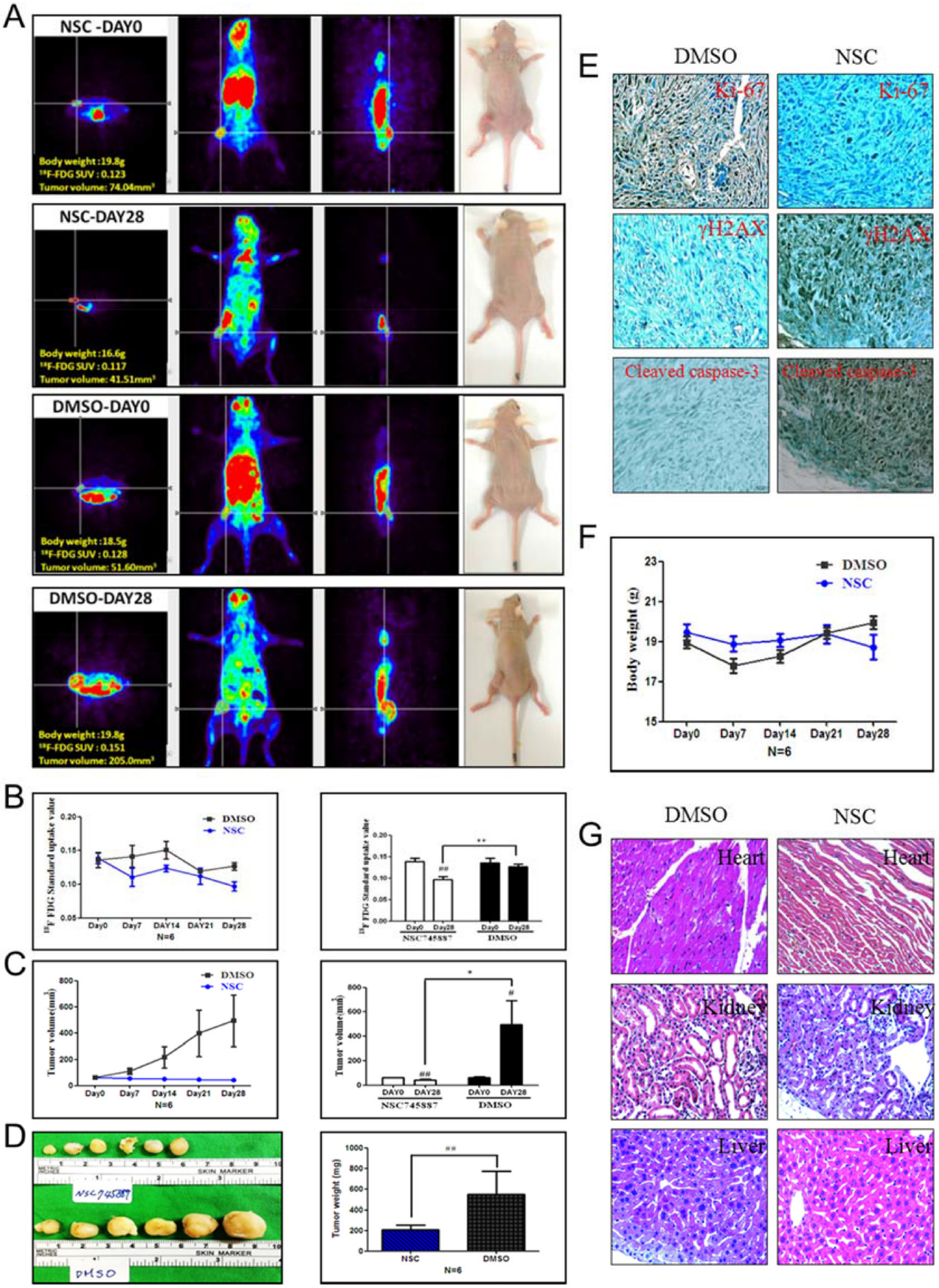
A
NSC -DAY0
Body weight :19.8g
18F-FDG SUV : 0.123
Tumor volume: 74.04mm3
NSC-DAY28
Body weight :16.6g
18F-FDG SUV : 0.117
Tumor volume: 41.51mm3
DMSO-DAY0
Body weight :18.5g
18F-FDG SUV : 0.128
Tumor volume: 51.60mm3
DMSO-DAY28
Body weight :19.8g
18F-FDG SUV : 0.151
Tumor volume: 205.0mm3
B
18F FDG Standard uptake value
DMSO
NSC
Day0 Day7 DAY14 DAY21 Day28
N=6
NSC745887
DMSO
C
Tumor volume(mm3)
DMSO
NSC
Day0 Day7 Day14 Day21 Day28
N=6
DAY0 DAY28
NSC745887
DMSO
D
NSC745887
DMSO
SKIN MARKER
Tumor weight (mg)
NSC
DMSO
N=6
E
DMSO
NSC
Ki-67
γH2AX
Cleaved caspase-3
F
Body weight (g)
DMSO
NSC
Day0 Day7 Day14 Day21 Day28
N=6
G
DMSO
NSC
Heart
Kidney
Liver

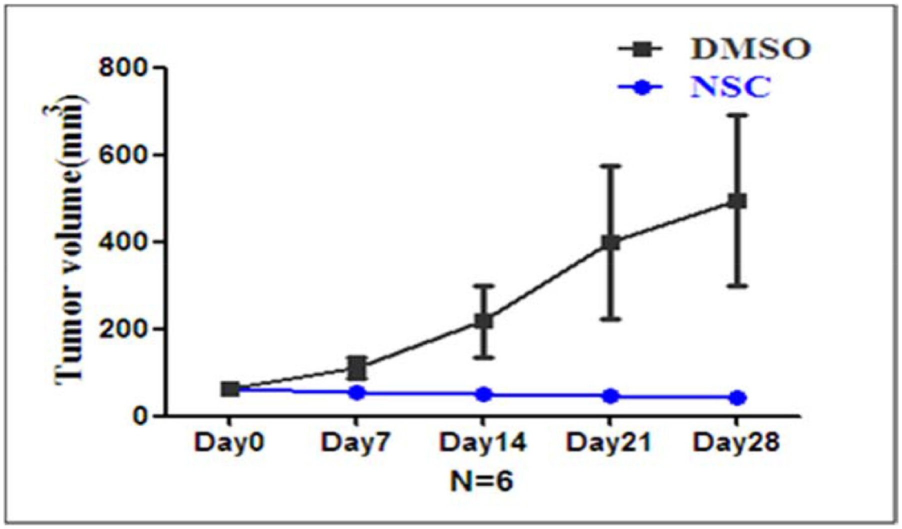

DMSO
NSC
Tumor volume(mm3)
800
600
400
200
0
Day0
Day7
Day14
Day21
Day28
N=6

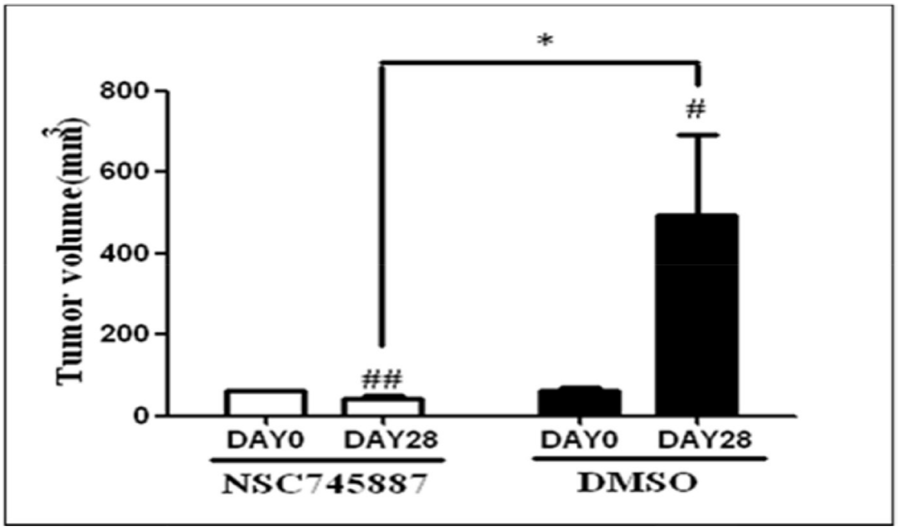

*
#
##
Tumor volume(mm3)
800
600
400
200
0
DAY0
DAY28
DAY0
DAY28
NSC745887
DMSO

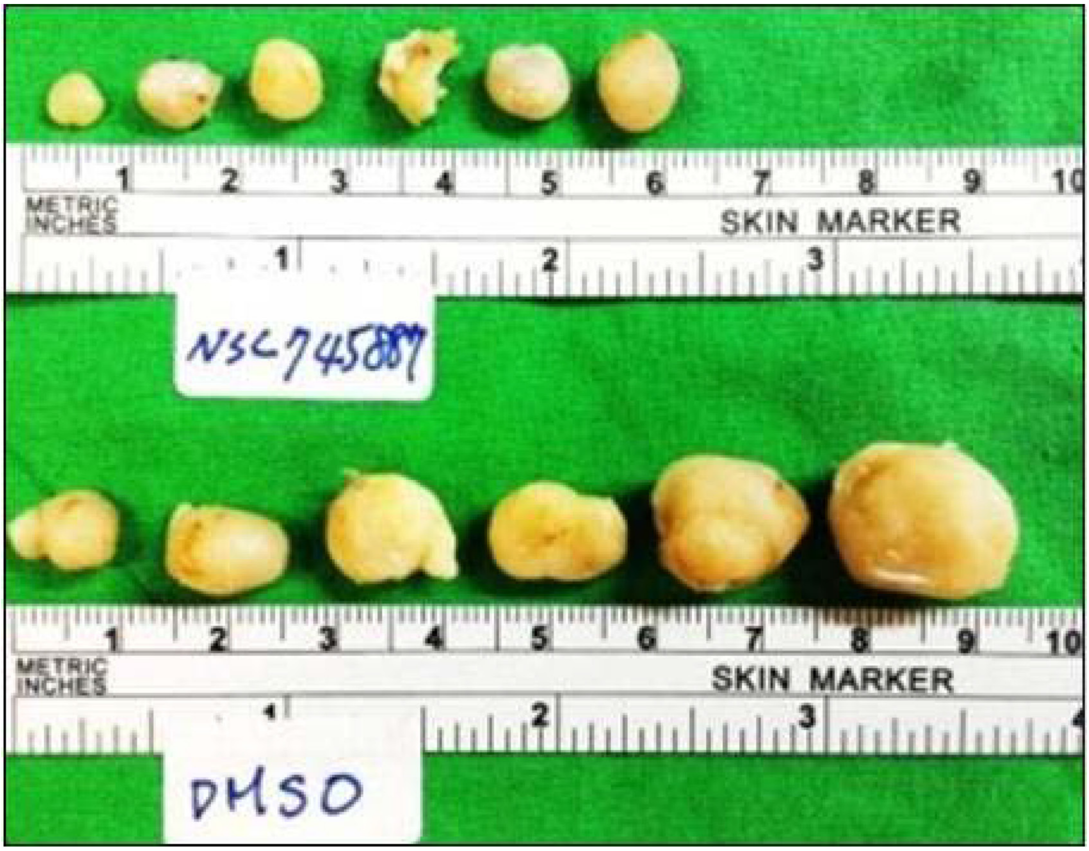

NSC745887
METRIC
INCHES
SKIN MARKER
DMSO

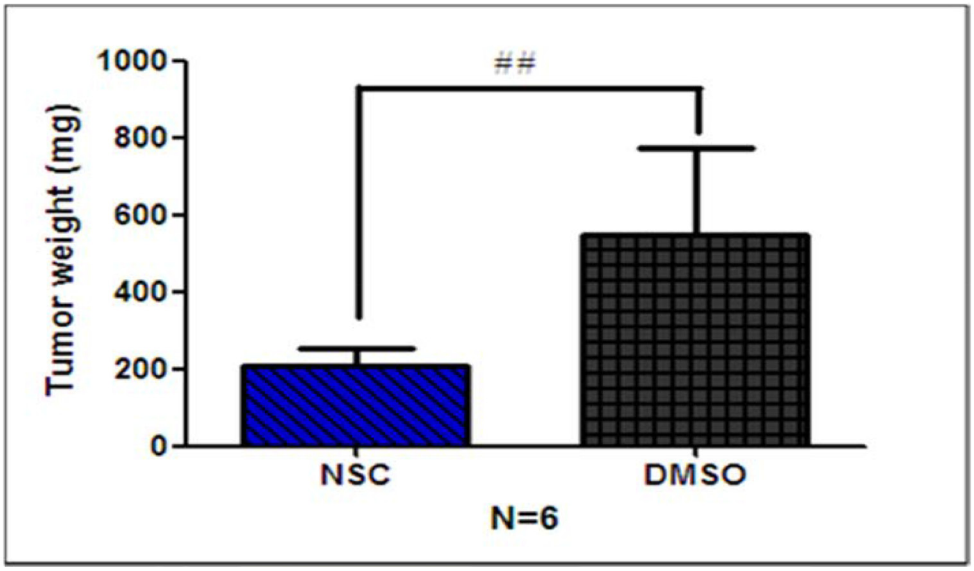

##
Tumor weight (mg)
1000
800
600
400
200
0
NSC
DMSO
N=6

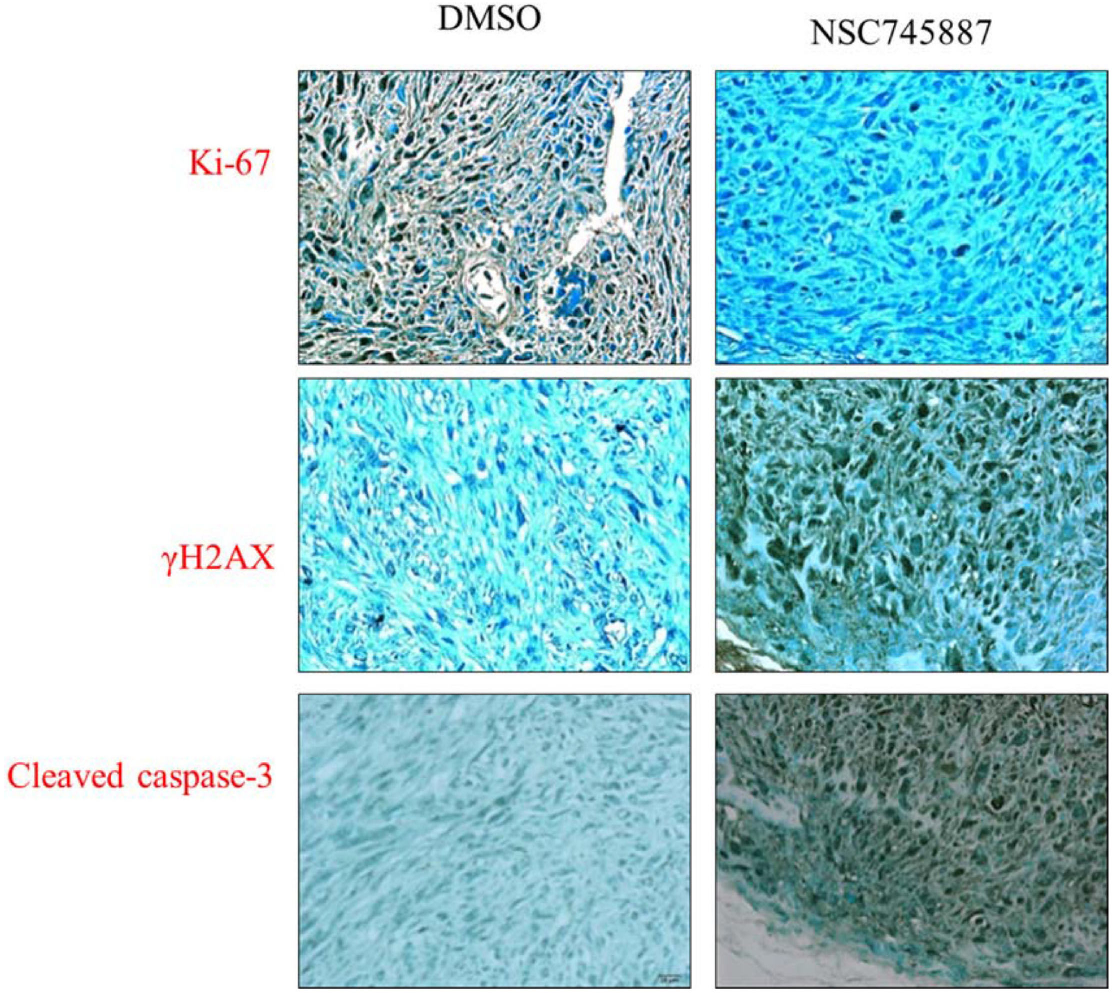
DMSO
NSC745887
Ki-67
γH2AX
Cleaved caspase-3

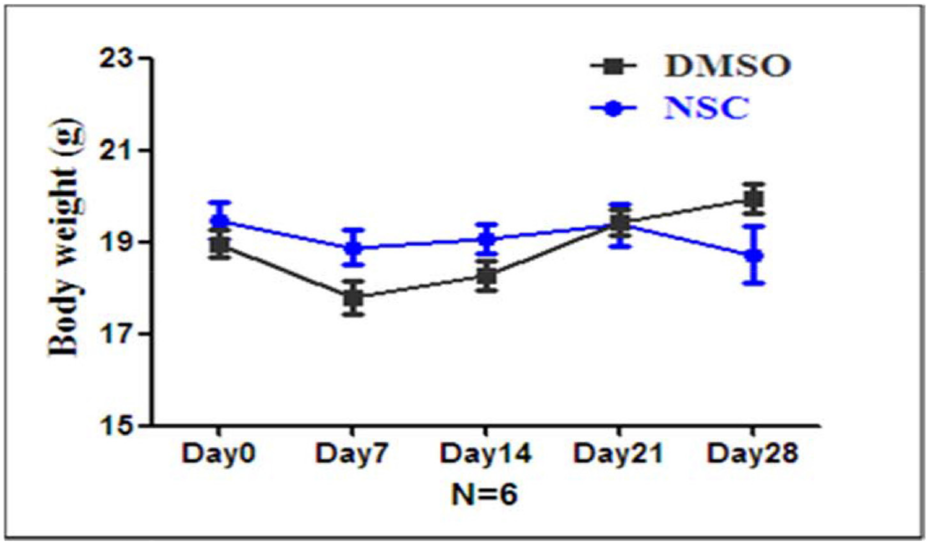
Body weight (g)
23
21
19
17
15
DMSO
NSC
Day0
Day7
Day14
Day21
Day28
N=6

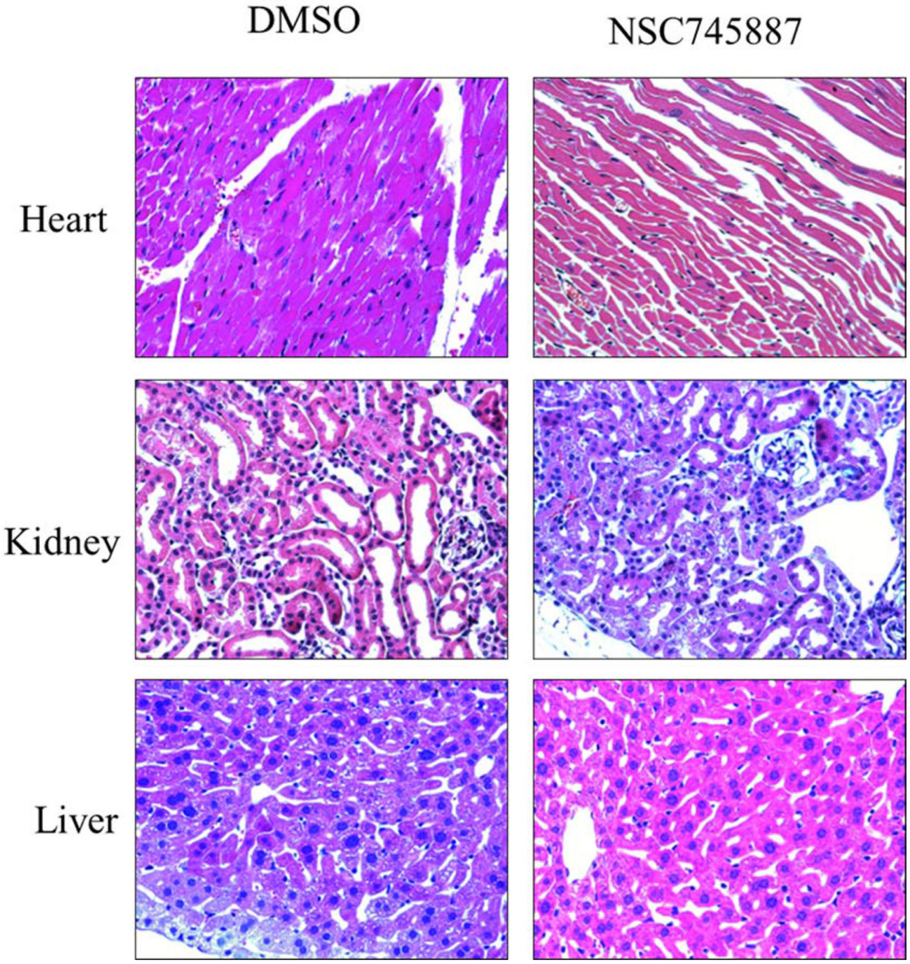
DMSO
NSC745887
Heart
Kidney
Liver

NSC745887-DAY0

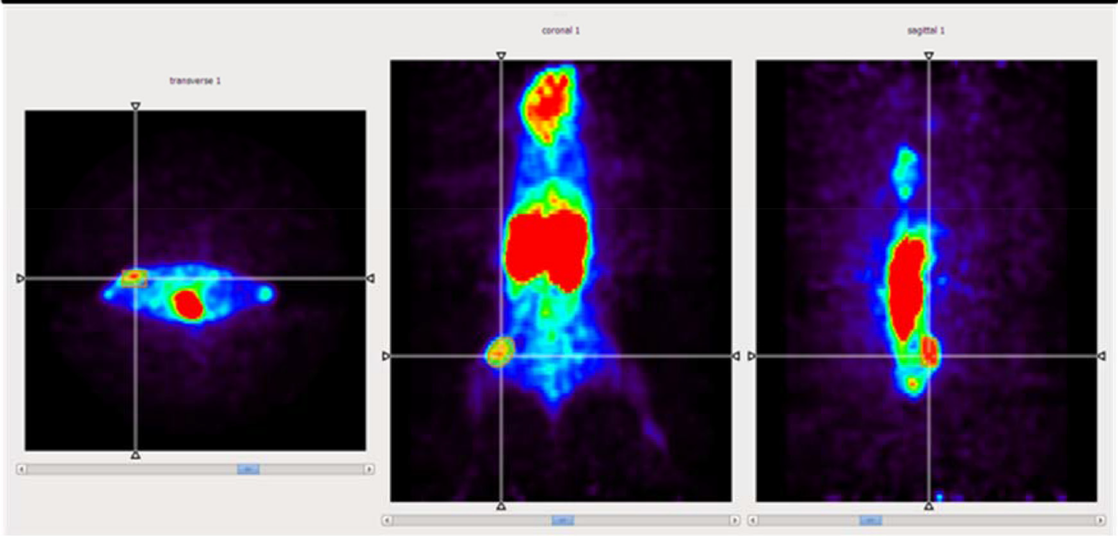

NSC745887-DAY7

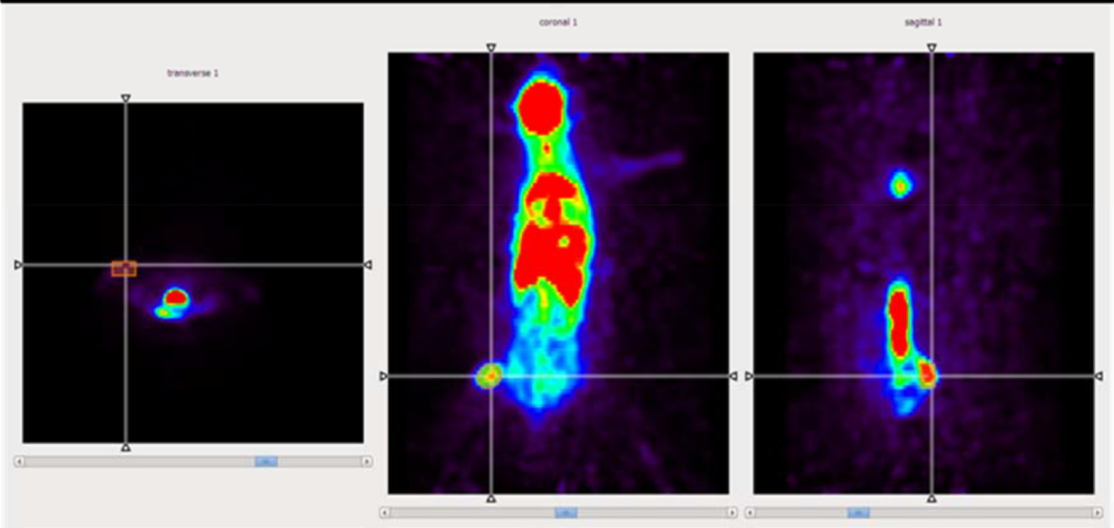

NSC745887-DAY14

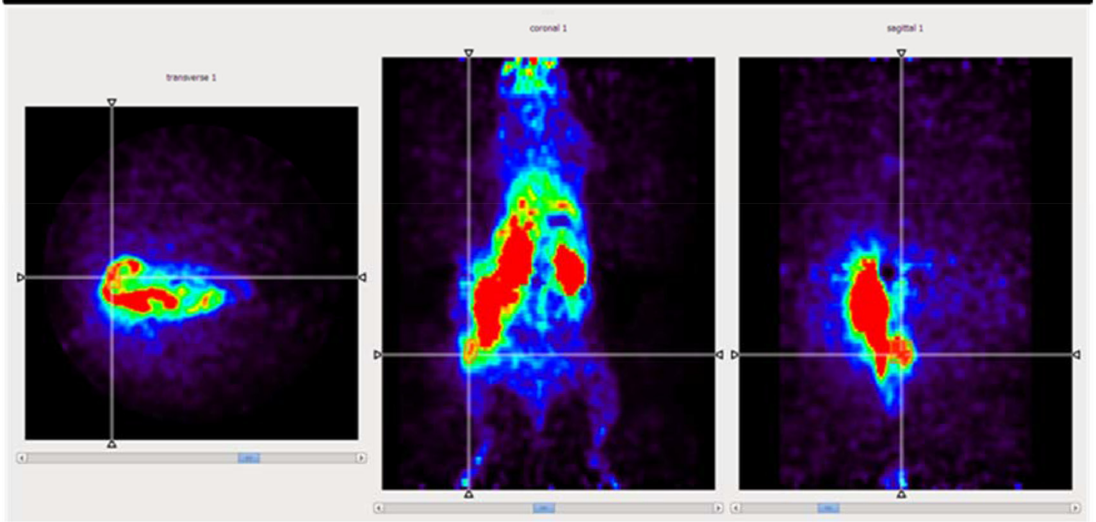

NSC745887-DAY21

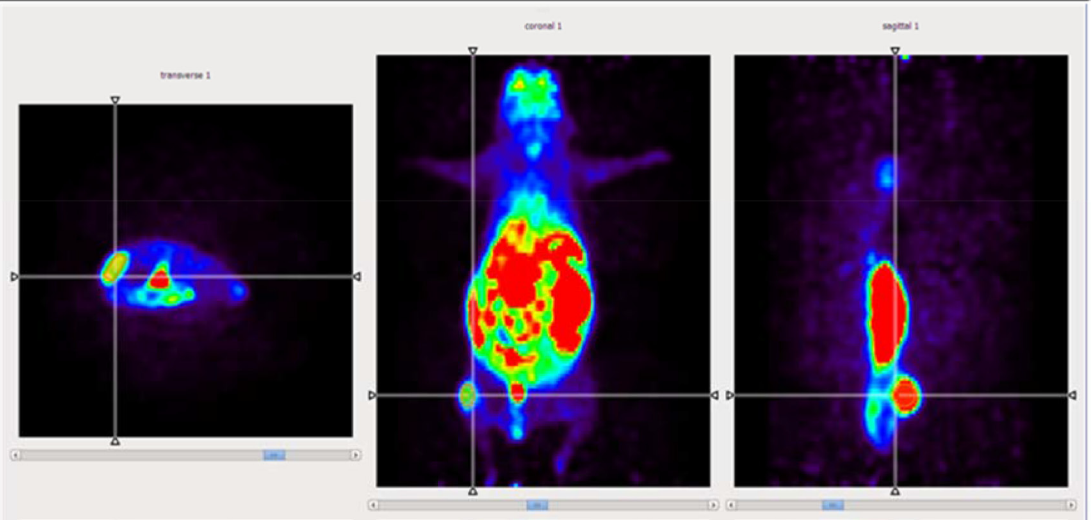

NSC745887-DAY28

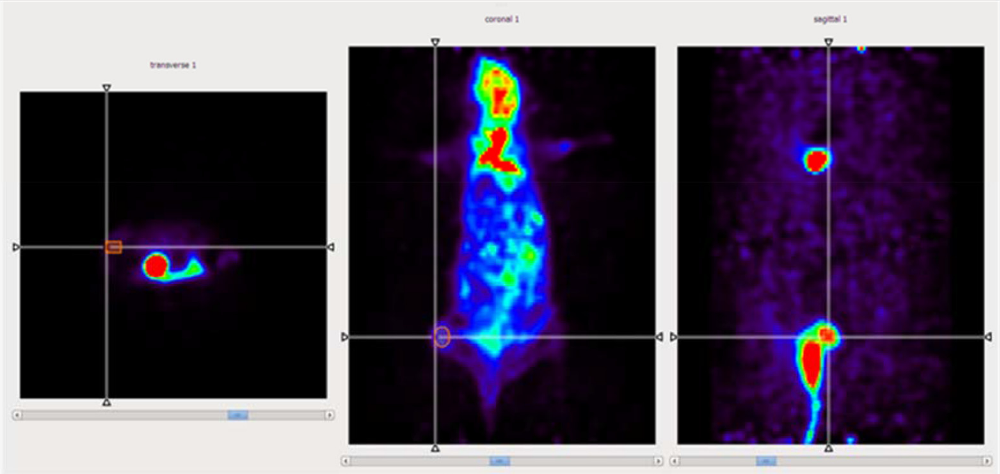

DMSO-DAY0

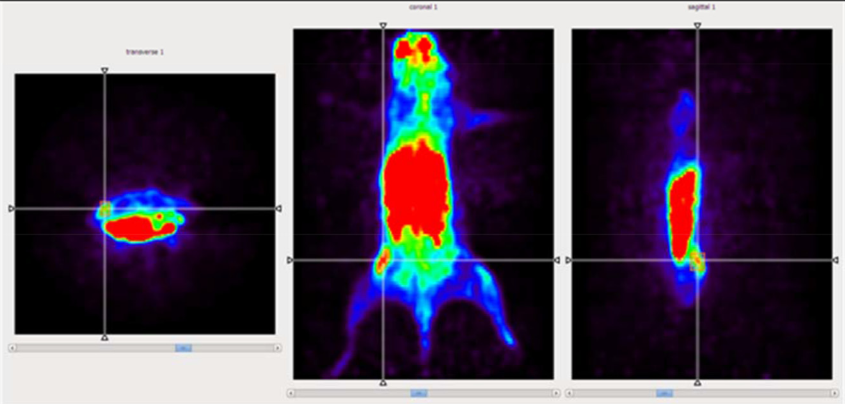

DMSO-DAY7

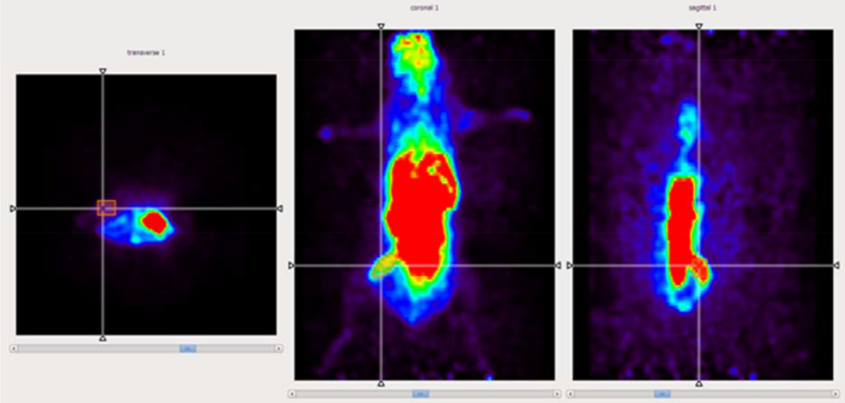

DMSO-DAY14

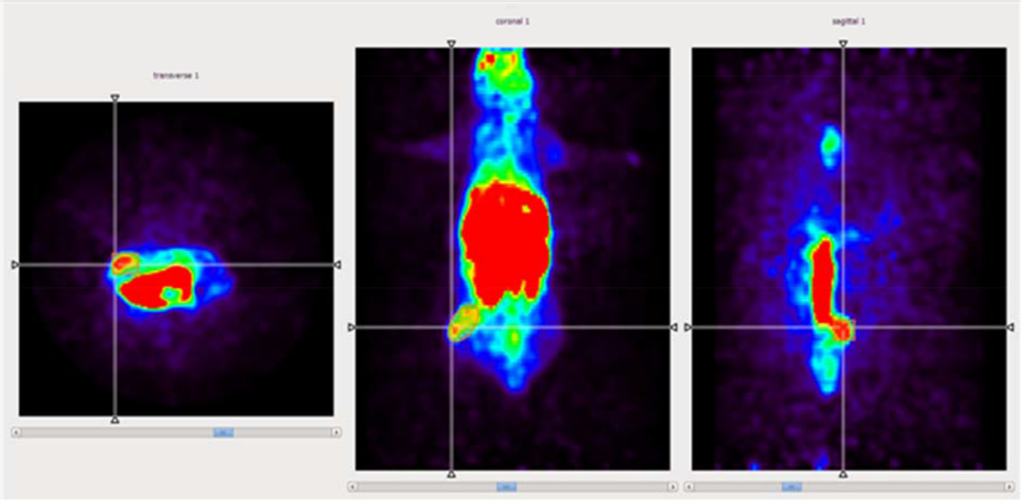

DMSO-DAY21

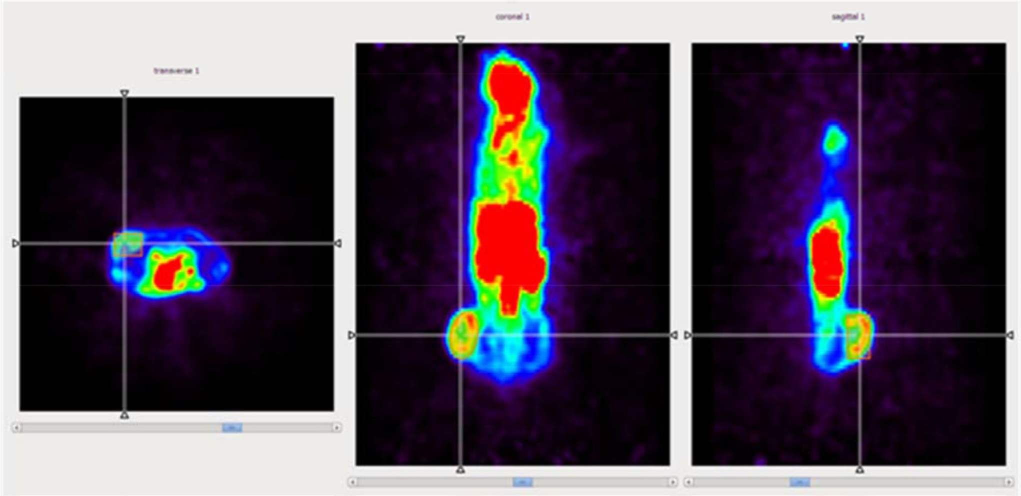

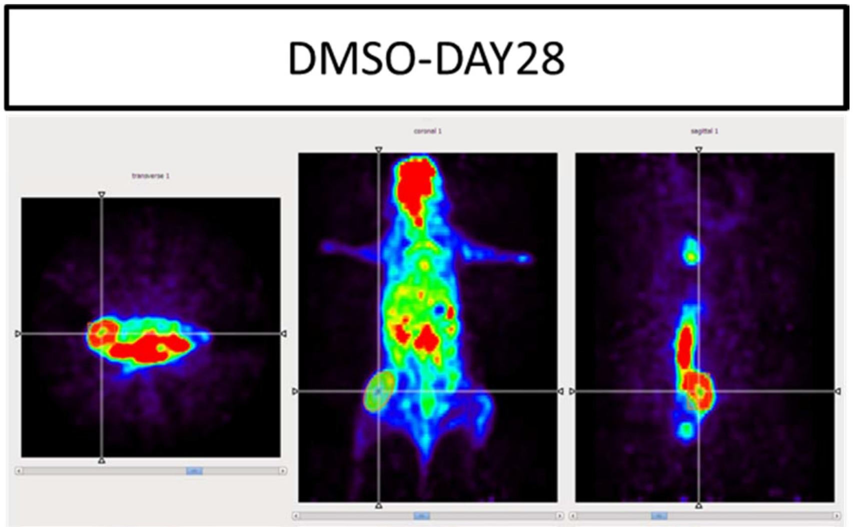

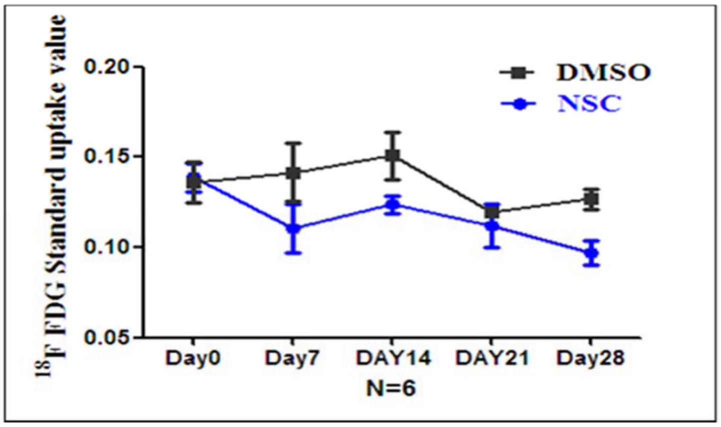

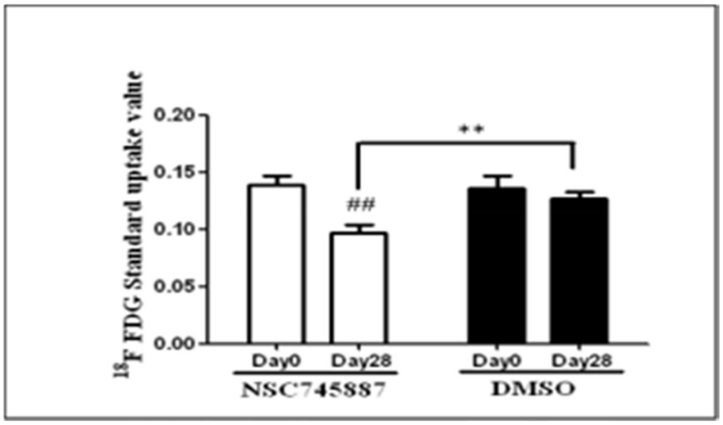

Supplementary Figure 7: NSC745887 promotes growth inhibition in xenografts. *In vivo* PET imaging data were analyzed in a NSC745887-treated group and a DMSO group using an animal-PET system. (**A**) [^{18}F]-FDG PET images from 15 to 35 min in U118MG expressing xenograft-bearing mice after intraperitoneal administration of radiotracers. (**B**) Quantitative analyses of specific [^{18}F]-FDG uptake values and (**C**) tumor volumes. (**D**) The tumor weight was measured at the endpoint. (**E**) Representative images of immunohistochemical staining of xenograft tumors. Protein levels of Ki-67, γH2AX and cleaved caspase-3. (**F**) Body weight were measured during treatment. (**G**) Representative image of H&E staining of the heart, liver, and kidneys in xenograft mice. $^{*}p < 0.05$, $^{**}p < 0.01$ comparing days 0 and 28. $^{\#}p < 0.05$, $^{\#\#}p < 0.01$ comparing the NSC745887 and DMSO groups.

Supplementary Table 1: Growth percentage of compounds NSC745887 in the NCI *in vitro* 60-cell Drug Screen Program. See Supplementary_Table_1

Supplementary Table 2: *In vitro* antitumor activity (GI_{50} in µM), TGI, and toxicity (LC_{50} in µM) data of NSC745887 in the NCI 60-cell Drug Screen Program. See Supplementary_Table_2

Supplementary Table 3: Median growth inhibitory concentration (GI_{50}, in µM) and GI_{50} selectivity rations of selected compounds NSC745887 in the NCI *in vitro* subpanel human cancer cell lines

	NSC745887		
	Subpanel MID/Selectivity ratio		
Leukemia	0.937047297	non-selective	17.57166667
Non-small cell lung cancer	1.048326553	non-selective	15.70644444
Colon cancer	2.51392382	non-selective	6.549714286
CNS cancer	1.929511075	non-selective	8.5335
Melanoma	1.302534454	non-selective	12.64111111
Ovarian cancer	1.180603448	non-selective	13.94666667
Renal cancer	0.567677392	non-selective	29.005
Prostate Cancer	0.894863193	non-selective	18.4
Breast cancer	0.568180178	non-selective	28.97933333
Mean			16.46548276

MID^a = Average sensitivity of all cell line in µM.
MID^b = Average sensitivity of all cell line of a particular subpanel in µM.
Selectivity ratio = MID^a : MID^b.
Ratios between 3 and 6 refer to moderate selectivity, ratios greater than 6 indicate high selectivity toward the corresponding cell line, while compounds not meeting either of these criteria are rated non-selective.
