# Supplementary material for: Identification and preclinical evaluation of the small molecule, NSC745887, for treating glioblastomas via suppressing DcR3-associated signaling pathways
Source: Oncotarget. 2017 Dec 27;9(15):11922–37. doi: 10.18632/oncotarget.23714 (PMC5844718; doi:10.18632/oncotarget.23714)
Supplement: Supplementary file 2 [file oncotarget-09-11922-s002.docx]

**Supplementary Table 1: Growth percentage of compounds NSC745887 in the NCI *in vitro* 60-cell Drug Screen Program**

| Panel/cell lines | | | Compounds/growth percent ***^a^*** |  |  |
| --- | --- | --- | --- | --- | --- |
|  | | **NSC745887** | | | |
| *Leukemia* | |  | | | |
|  | HL-60(TB) | 102.34 | | | |
|  | K-562 | 37.23 | | | |
|  | MOLT-4 | 22.91 | | | |
|  | SR | 82.92 | | | |
| *Non-small cell lung cancer* | | | | | |
|  | A549/ATCC | 47.13 | | | |
|  | EKVX | 82.57 | | | |
|  | NCI-H226 | 86.72 | | | |
|  | NCI-H23 | 65.13 | | | |
|  | NCI-H322M | 96.35 | | | |
|  | NCI-H460 | 31.69 | | | |
|  | NCI-H522 | 24.09 | | | |
| *Colon cancer* | |  | | | |
|  | COLO 205 | 71.56 | | | |
|  | HCC-2998 | -7.15 | | | |
|  | HCT-116 | 52.27 | | | |
|  | HCT-15 | 52.15 | | | |
|  | HT29 | 81.90 | | | |
|  | KM12 | 61.00 | | | |
|  | SW-620 | 39.19 | | | |
| *CNS cancer* | |  | | | |
|  | SF-268 | 67.98 | | | |
|  | SF-295 | 80.81 | | | |
|  | SF-539 | 40.72 | | | |
|  | SNB-19 | 87.30 | | | |
|  | SNB-75 | 104.52 | | | |
|  | U251 | 63.47 | | | |
| *Melanoma* | |  | | | |
|  | LOX IMVI | 44.18 | | | |
|  | MALME-3M | 180.88 | | | |
|  | M14 | 59.81 | | | |
|  | MDA-MB-435 | 102.03 | | | |
|  | SK-MEL-2 | 6.66 | | | |
|  | SK-MEL-28 | 97.49 | | | |
|  | SK-MEL-5 | 66.57 | | | |
|  | UACC-257 | 91.06 | | | |
|  | UACC-62 | 90.47 | | | |
| *Ovarian cancer* | | | | | |
|  | IGROV1 | 3.44 | | | |
|  | OVCAR-3 | 71.65 | | | |
|  | OVCAR-4 | 94.22 | | | |
|  | OVCAR-5 | 106.59 | | | |
|  | OVCAR-8 | 47.54 | | | |
|  | NCI/ADR-RES | 67.96 | | | |
|  | SK-OV-3 | 78.16 | | | |
| *Renal cancer* | |  | | | |
|  | 786-0 | 28.45 | | | |
|  | A489 | 98.83 | | | |
|  | ACHN | 46.52 | | | |
|  | CAKI-1 | 87.92 | | | |
|  | RXF 393 | 104.88 | | | |
|  | SN12C | 64.65 | | | |
|  | TK-10 | 126.89 | | | |
|  | UO-31 | 51.45 | | | |
| *Prostate cancer* | | | | | |
|  | DU-145 | 93.92 | | | |
| *Breast cancer* | |  | | | |
|  | MCF7 | 39.68 | | | |
|  | MDAMB231/ATCC | 81.39 | | | |
|  | HS578-T | 76.75 | | | |
|  | T-47D | 85.19 | | | |
|  | MDA-MB-468 | 75.28 | | | |
| Mean | | 69.36 | | | |
| Delta | | 76.51 | | | |
| Range | | 188.03 | | | |

^a^Data obtained from NCI *in vitro* 60-cell drug screen program at 10^-5^ molar concentration.
