# Supplementary material for: Identification and preclinical evaluation of the small molecule, NSC745887, for treating glioblastomas via suppressing DcR3-associated signaling pathways
Source: Oncotarget. 2017 Dec 27;9(15):11922–37. doi: 10.18632/oncotarget.23714 (PMC5844718; doi:10.18632/oncotarget.23714)
Supplement: Supplementary file 3 [file oncotarget-09-11922-s003.docx]

**Supplementary Table 2: *In vitro* antitumor activity (GI_50_ in μM), TGI, and toxicity (LC_50_ in μM) data of NSC745887 in the NCI 60-cell Drug Screen Program**

| Panel/cell lines | | | **NSC745887** | | |
| --- | --- | --- | --- | --- | --- |
|  | | GI50 (μM) | | TGI (μM) | LC50 (μM) |
| *Leukemia* | |  | | | |
|  | CCRF-CEM | 3.21 | | >100 | >100 |
|  | HL-60(TB) | 7.42 | | 36.5 | >100 |
|  | K-562 | 8.63 | | >100 | >100 |
|  | MOLT-4 | 5.24 | | >100 | >100 |
|  | RPMI-8226 | 2.43 | | 94.7 | >100 |
|  | SR | 78.5 | | >100 | >100 |
| *Non-small cell lung cancer* | | | | | |
|  | A549/ATCC | 0.802 | | - | >100 |
|  | EKVX | 10.9 | | >100 | >100 |
|  | HOP-62 | 5.54 | | >100 | >100 |
|  | HOP-92 | 28.3 | | >100 | >100 |
|  | NCI-H226 | 32.3 | | >100 | >100 |
|  | NCI-H23 | 3.94 | | 73.7 | >100 |
|  | NCI-H322M | 50.1 | | >100 | >100 |
|  | NCI-H460 | 0.646 | | 22.1 | >100 |
|  | NCI-H522 | 8.83 | | >100 | >100 |
| *Colon cancer* | |  | | | |
|  | COLO 205 | 0.637 | | 23.6 | >100 |
|  | HCC-2998 | 0.431 | | 15.2 | 85.7 |
|  | HCT-116 | 10.1 | | >100 | >100 |
|  | HCT-15 | 10.0 | | >100 | >100 |
|  | HT29 | 8.53 | | >100 | >100 |
|  | KM12 | 3.75 | | >100 | >100 |
|  | SW-620 | 12.4 | | >100 | >100 |
| *CNS cancer* | |  | | | |
|  | SF-268 | 19.5 | | >100 | >100 |
|  | SF-295 | 3.53 | | >100 | >100 |
|  | SF-539 | 0.351 | | 24.9 | >100 |
|  | SNB-19 | 8.25 | | >100 | >100 |
|  | SNB-75 | 11.9 | | >100 | >100 |
|  | U251 | 7.67 | | >100 | >100 |
| *Melanoma* | |  | | | |
|  | LOX IMVI | 6.28 | | >100 | >100 |
|  | MALME-3M | 2.39 | | 8.39 | 57.2 |
|  | M14 | 6.32 | | >100 | >100 |
|  | MDA-MB-435 | 13.7 | | 52.3 | >100 |
|  | SK-MEL-2 | 16.6 | | 71.3 | >100 |
|  | SK-MEL-28 | 51.9 | | >100 | >100 |
|  | SK-MEL-5 | 1.12 | | 2.96 | 7.83 |
|  | UACC-257 | 3.36 | | 27.4 | >100 |
|  | UACC-62 | 12.1 | | >100 | >100 |
| *Ovarian cancer* | | | | | |
|  | OVCAR-3 | 15.8 | | 57.4 | >100 |
|  | OVCAR-4 | 6.55 | | 39.1 | >100 |
|  | OVCAR-5 | 41.3 | | >100 | >100 |
|  | OVCAR-8 | 1.90 | | >100 | >100 |
|  | NCI/ADR-RES | 4.13 | | >100 | >100 |
|  | SK-OV-3 | 14.0 | | >100 | >100 |
| *Renal cancer* | |  | | | |
|  | 786-0 | 7.53 | | >100 | >100 |
|  | A489 | 79.6 | | >100 | >100 |
|  | ACHN | 7.18 | | >100 | >100 |
|  | CAKI-1 | 11.4 | | >100 | >100 |
|  | SN12C | 39.2 | | >100 | >100 |
|  | TK-10 | 57.2 | | >100 | >100 |
|  | UO-31 | 0.925 | | 18.0 | >100 |
| *Prostate cancer* | | | | | |
|  | PC-3 | 20.9 | | >100 | >100 |
|  | DU-145 | 15.9 | | 61.1 | >100 |
| *Breast cancer* | |  | | | |
|  | MCF7 | 0.166 | | 17.6 | 79.5 |
|  | MDAMB231/ATCC | 52.6 | | >100 | >100 |
|  | HS 578T | 88.7 | | >100 | >100 |
|  | BT-549 | 14.2 | | 96.3 | >100 |
|  | T-47D | 2.51 | | 76.1 | >100 |
|  | MDA-MB-468 | 15.7 | | 67.4 | >100 |

Data obtained from NCI *in vitro* 60-cell drug screen program at 10^-5^ molar concentration.
